# Photoreactions of Endohedral Metallofullerene with Siliranes: Electronic Properties of Carbosilylated Lu_3_N@*I_h_*-C_80_ †

**DOI:** 10.3390/molecules22050850

**Published:** 2017-05-20

**Authors:** Masahiro Kako, Kazuya Minami, Taiki Kuroiwa, Shinpei Fukazawa, Yuki Arikawa, Michio Yamada, Yutaka Maeda, Qiao-Zhi Li, Shigeru Nagase, Takeshi Akasaka

**Affiliations:** 1 Department of Engineering Science, The University of Electro-Communications, Chofu 182-8585, Japan; minami@chemk.pc.uec.ac.jp (K.M.); kuroiwa@chemk.pc.uec.ac.jp (T.K.); fukazawa@chemk.pc.uec.ac.jp (S.F.); arikawa@chemk.pc.uec.ac.jp (Y.A.); 2 Department of Chemistry, Tokyo Gakugei University, Tokyo 184-8501, Japan; myamada@u-gakugei.ac.jp (M.Y.); ymaeda@u-gakugei.ac.jp (Y.M.); 3 Fukui Institute for Fundamental Chemistry, Kyoto University, Kyoto 606-8103, Japan; lqz1153672945@stu.xjtu.edu.cn; 4 Life Science Center of Tsukuba Advanced Research Alliance, University of Tsukuba, Ibaraki 305-8577, Japan; 5 Foundation for Advancement of International Science, Ibaraki 305-0821, Japan; 6 School of Materials Science and Engineering, Huazhong University of Science and Technology, Wuhan 430074, China

**Keywords:** endohedral metallofullerene, Lu_3_N@*I_h_*-C_80_, carbosilylation, silirane, redox property

## Abstract

Photochemical carbosilylation of Lu_3_N@*I_h_*-C_80_ was performed using siliranes (silacyclopropanes) to afford the corresponding [5,6]- and [6,6]-adducts. Electrochemical studies indicated that the redox potentials of the carbosilylated derivatives were shifted cathodically in comparison with those of the [5,6]-pyrrolidino adducts. The electronic effect of the silirane addends on Lu_3_N@*I_h_*-C_80_ was verified on the basis of density functional theory calculations.

## 1. Introduction

Endohedral metallofullerenes (EMFs) [1,2,3,4,5,6,7,8,9,10,11,12] have attracted much interest because of their fascinating structural and electronic properties. On the basis of the encapsulated metals, the properties and reactivities of EMFs are significantly different from those of empty fullerenes. For the last few decades, trimetallic nitride template endohedral metallofullerenes (TNT EMFs), M_3_N@*I_h_*-C_80_ (M = Sc, Lu, Y, and Gd), have been extensively studied as representatives of cluster fullerenes, for which potential applications have been explored in the fields of molecular electronics, nanomaterials, and biochemistry [1,2,3,4,5,6,7,8,9,10,11,12,13,14]. For example, Lu_3_N@*I_h_*-C_80_ is an attractive compound from a practical viewpoint. The reduction potential of Lu_3_N@*I_h_*-C_80_ is lower than those of the other trimetallic nitride template (TNT) EMFs, M_3_N@*I_h_*-C_80_ (M = Sc, Y, etc.), C_60_, and C_70_. This property of Lu_3_N@*I_h_*-C_80_ is expected to be advantageous with respect to its use as an acceptor in organic photovoltaic (OPV) devices. Previously, OPV devices using some Lu_3_N@*I_h_*-C_80_ derivatives with lower reduction potentials were reported to show higher power conversion efficiency than that of C_60_-based analogous devices [15,16,17].

Meanwhile, the exohedral functionalization of EMFs has been studied extensively as an effective method for modifying the properties of EMFs for many applications [1,2,3,4,5,6,7,8,9,10,11,12]. As a part of our study of the chemical derivatization of EMFs, we reported the addition reactions of Lu_3_N@*I_h_*-C_80_ with disilirane, silylene, and digermirane to afford the corresponding silylated and germylated adducts [18,19,20]. The redox potentials of these derivatives were shifted cathodically relative to that of pristine Lu_3_N@*I_h_*-C_80_, as expected from the electron-donating effect of silyl and germyl groups. These results prompted us to apply alternative silylation methods to Lu_3_N@*I_h_*-C_80_, which will enable fine-tuning of its electronic properties. More recently, we reported the photochemical addition of Sc_3_N@*I_h_*-C_80_ and silirane (silacyclopropane) **1** to afford three isomeric carbosilylated derivatives of Sc_3_N@*I_h_*-C_80_ [21]. Spectroscopic measurements indicated that the addition of **1** occurred at the [5,6] (a pentagon and a hexagon), and the [6,6] (two hexagons) ring junctions. In addition, the oxidation potentials of the carbosilylated adducts were shifted moderately compared to that of pristine Sc_3_N@*I_h_*-C_80_. Herein, we report the carbosilylation of Lu_3_N@*I_h_*-C_80_ via photoreactions using siliranes **1** [21] and **2** [22] to afford the corresponding [5,6]- and [6,6]-adducts. The electronic effect of silirane addition on Lu_3_N@*I_h_*-C_80_ is discussed in comparison with that of the 1,3-dipolar addition of azomethine ylides [23,24].

## 2. Results and Discussion

### 2.1. Preparation and Structural Analysis of the Carbosilylated Derivatives of Lu_3_N@I_h_-C_80_

**Scheme 1:**
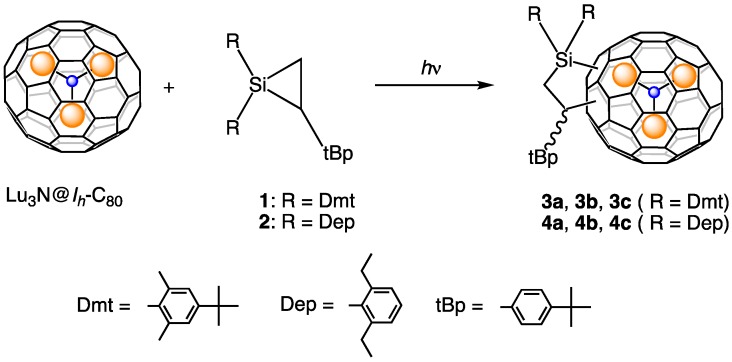
Photoreactions of Lu_3_N@*I_h_*-C_80_ with siliranes **1** and **2**.

A toluene solution of Lu_3_N@*I_h_*-C_80_ and **1** was photo-irradiated using two 500 W halogen lamps (cutoff < 400 nm) (Scheme 1). After photolysis for 60 h, preparative high performance liquid chromatography (HPLC) of the reaction mixture was performed to separate three compounds **3a**, **3b**, and **3c** (Figures S1 and S2, in the Supporting Information). Matrix-assisted laser desorption ionization time-of-flight (MALDI-TOF) mass spectrometry of **3a**, **3b**, and **3c** exhibited molecular ion peaks at *m*/*z* 2009 (M^−^), as expected for 1:1 adducts of Lu_3_N@*I_h_*-C_80_ and **1** (Figure S3). The spectra also show base peaks at *m*/*z* 1499 for the fragment ion Lu_3_N@*I_h_*-C_80_^−^. Similarly, photoreaction of Lu_3_N@*I_h_*-C_80_ and **2** provided three isomeric adducts **4a**, **4b**, and **4c**. The yields of **3a**, **3b**, **4a**, and **4b** were calculated to be 30%, 7%, 26% and 16%, respectively, whereas those of **3c** and **4c** were not determined because they contained inseparable impurities.

**Figure 1:**
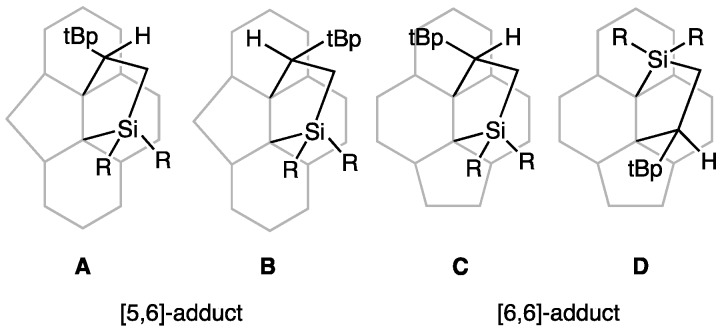
Possible addition patterns of 1,2-adducts derived from Lu_3_N@*I_h_*-C_80_ and siliranes.

**Figure 2:**
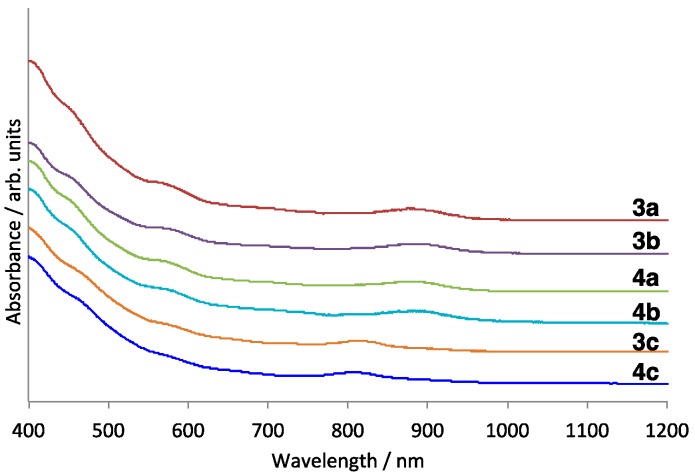
Vis–NIR absorption spectra of **3a**–**c** and **4a**–**c** in CS_2_.

**Figure 3:**
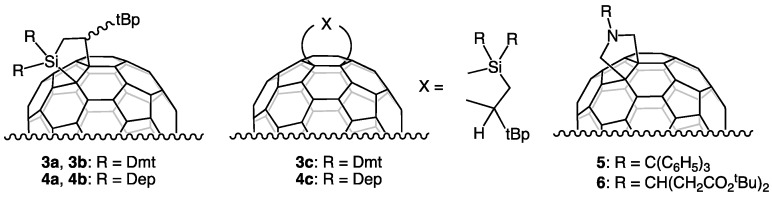
Partial structures of the Lu_3_N@*I*_h_-C_80_ derivatives.

Assuming 1,2-addition of **1** and **2** to Lu_3_N@*I_h_*-C_80_, four structures **A**–**D** are possible for the addition sites (Figure 1). To obtain an insight into their addition patterns, visible-near-infrared (vis-NIR) spectroscopy was conducted for the adducts (Figure 2). Previous studies indicate that the vis-NIR spectra of the [5,6]- and [6,6]-pyrrolidino derivatives of Lu_3_N@*I_h_*-C_80_ exhibit absorption maxima at approximately 870 and 820 nm, respectively [24,25]. The vis-NIR spectra of **3a**, **3b**, **4a**, and **4b** show absorption maxima around 880 nm, which are similar to those of the [5,6]-pyrrolidino derivatives [24,25] (Figure 2). Meanwhile, the vis-NIR spectra of **3c** and **4c** show broad absorption maxima at 811 and 803 nm, which resemble those of the [6,6]-pyrrolidino derivatives [24,25]. Therefore, **3a**, **3b**, **4a**, and **4b** are assignable to the [5,6]-adducts **A** and **B**. On the other hand, **3c** and **4c** are probably the [6,6]-adducts **C** and/or **D** (See also Figure 3).

In the ^1^H-NMR spectrum of **3a**, signals for four methyl groups and three *tert*-butyl groups, as well as aromatic protons were observed (Figure S5). The ring protons of the silacyclopentane addend of **3a** constitute an ABX spin system at 3.66, 2.85, and 1.84 ppm as double doublets. Meanwhile, the ^13^C-NMR spectrum of **3a** exhibited 86 quaternary and six tertiary sp^2^ carbon signals, as well as two sp^3^ carbon signals of the *I_h_*-C_80_ cage (Figure S7). The carbon signals of the methylene and methine of the silacyclopentane ring, the three *tert*-butyl and four methyl groups were also shown in the spectrum. These spectral data are consistent with the structure of **3a** as the [5,6]-adduct with *C*_1_ symmetry. The ^1^H-NMR spectrum of **3b** is very similar to that of **3a** (Figure S6). On the basis of the vis-NIR and ^1^H-NMR spectra, **3a** and **3b** were determined to be a pair of diastereomers **A** and **B**, although the absolute configurations of 4-*tert*-butylphenyl (denoted as tBp) groups remain unknown.

The ^13^C-NMR spectrum of **4a**, the main product obtained from **2**, is similar to that of **3a**, showing 86 sp^2^ carbon atom signals (Figure S10). The carbon signals of the silirane addend and two sp^3^ carbon signals of the *I_h_*-C_80_ cage were also observed. The ^1^H-NMR spectra of **4a** and **4b** also indicate the structures of the silirane addends as for the case of **3a** and **3b** (Figures S8 and S9). As shown in the vis-NIR spectra, it is suggested that **4a** and **4b** are a pair of diastereomers of the [5,6]-adducts. On the other hand, spectroscopic studies of **3c** and **4c** have hitherto been unsuccessful because of their low yields and impurities.

### 2.2. Electrochemical Studies

The electrochemical properties of **3a**, **3b**, **4a**, and **4b** were measured using cyclic voltammetry (CV) and differential pulse voltammetry (DPV). The redox potentials of the related compounds are summarized in Table 1. As shown in Figures S11 and S12, the oxidation of **3a**, **3b**, **4a**, and **4b** resulted in the removal of the silirane addends during the electrochemical analyses, affording pristine Lu_3_N@*I_h_*-C_80_. The first oxidation (*E*^ox^_1_) potentials of **3a**, **3b**, **4a**, and **4b** are lower than that of Lu_3_N@*I_h_*-C_80_ within the range of 360–400 mV. These remarkable cathodic shifts of the oxidation potentials are commonly observed for silylated fullerenes because of the electron-donating properties of the silyl groups [18,19,20,21]. Meanwhile, the values of the first reduction (*E*^red^_1_) potentials of **3a**, **3b**, **4a**, and **4b** are close to that of Lu_3_N@*I_h_*-C_80_. These redox data are compared to those of **5** [23] and **6** [24] as the [5,6]-pyrrolidino adducts of Lu_3_N@*I_h_*-C_80_. The first reduction potentials of **5** and **6** are −1.13 and −1.14 V, respectively. According to the reported voltammograms, the *E*^ox^_1_ potentials of **5** and **6** are both about +0.34 V, respectively, although the exact *E*^ox^_1_ values are not given in the literature [23,24]. As a result, the redox potentials of **3a**, **3b**, **4a**, and **4b** shift cathodically compared to those of **5** and **6**. These effects of silyl functional groups are also observed for carbosilylated derivatives of Sc_3_N@*I_h_*-C_80_ [21].

The *E*^red^_1_ potentials of **3a**, **3b**, **4a**, and **4b** are interesting from the viewpoints of Lu_3_N@*I_h_*-C_80_-based OPV acceptors, for which optimization of the lowest unoccupied molecular orbital (LUMO) levels is an important factor in improving the open circuit voltages of the corresponding solar cells [15,16]. The *E*^red^_1_ potentials of the [5,6]-pyrrolidino adducts **5** [23] and **6** [24] are shifted positively relative to that of Lu_3_N@*I_h_*-C_80_. In contrast, **3a**, **3b**, **4a**, and **4b** maintain the low reduction potential of pristine Lu_3_N@*I_h_*-C_80_. Although the functions of **3a**, **3b**, **4a**, and **4b** as OPV acceptors are unknown, carbosilylation would be an effective method to adjust the electronic properties of fullerenes for electronic functional materials.

### 2.3. Theoretical Calculations

**Figure 4:**
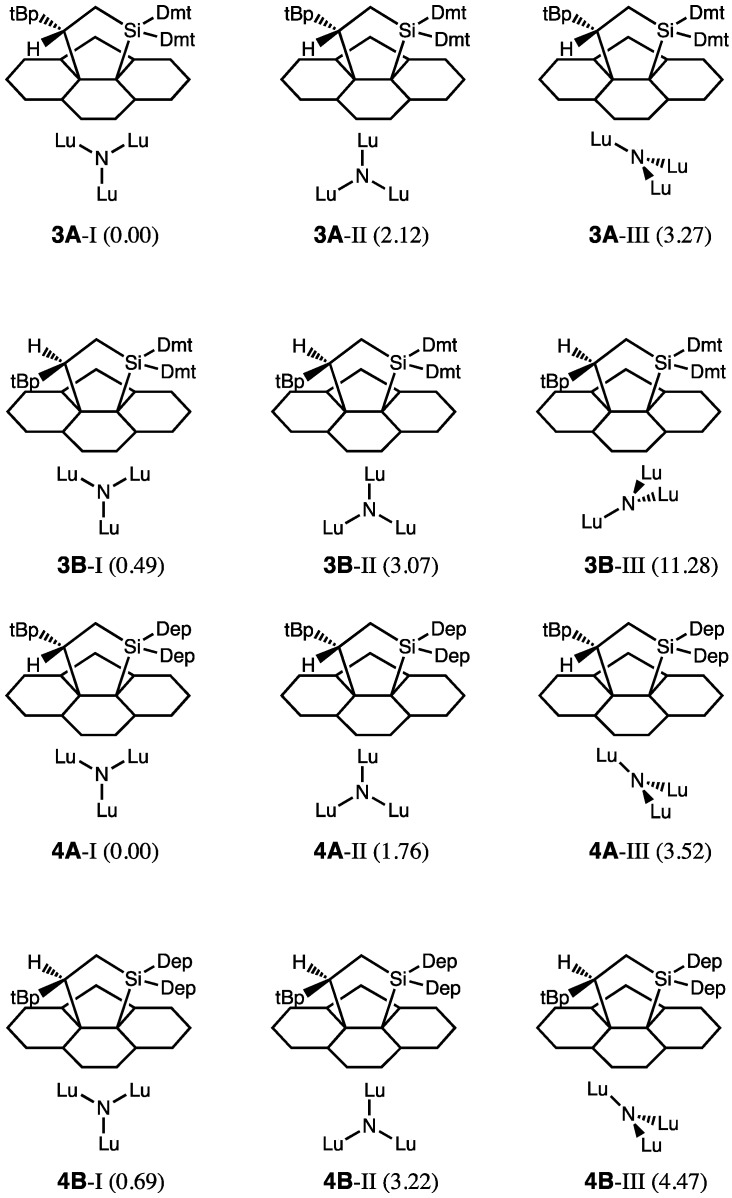
Representation of the configurations of silirane addends and the orientations of the Lu_3_N cluster in the optimized structures. Values in the parentheses are the relative energies in kcal/mol. The values of **3A**-II, **3A**-III, **3B**-I, **3B**-II, and **3B**-III are relative to that of **3A**-I. For **4A**-II, **4A**-III, **4B**-I, **4B**-II, and **4B**-III, the values are relative to that of **4A**-I.

To obtain an insight into the structural and electronic properties of **3a**, **3b**, **4a**, and **4b**, isomers of [5,6]-carbosilylated Lu_3_N@*I_h_*-C_80_ were calculated by the density functional theory using the B3LYP method [26,27,28]. The partial structures of the calculated molecules are shown in Figure 4 with the relative energies. The optimized structures of these molecules are also shown in Figures S13–S16. These structures were calculated with a few different orientations of the Lu_3_N cluster. As for the configurations of the silirane addend, **3A**-I, **3A**-II, **3A**-III, **4A**-I, **4A**-II, and **4A**-III are calculated molecules for the diastereomer **A** in Figure 1, while **3B**-I, **3B**-II, **3B**-III, **4B**-I, **4B**-II, and **4B**-III correspond to **B**. These structures are grouped into four configurations: (**3A**-I, **3A**-II, **3A**-III); (**3B**-I, **3B**-II, **3B**-III); (**4A**-I, **4A**-II, **4A**-III); and (**4B**-I, **4B**-II, **4B**-III). Each of the structural groups has three orientations of the Lu_3_N cluster, as shown in Figure 4. As expected, the relative energies of the adducts vary depending on the orientations of the Lu_3_N cluster and the configurations of the silirane addends. Among these isomers, **3A**-I, **4A**-I, **3B**-I, and **4B**-I, in which the Y-shaped Lu_3_N clusters straddle the addition sites, have relatively lower energies. These calculations indicate that the orientations of the Lu_3_N clusters in **3a**, **3b**, **4a**, and **4b** should be somewhat restricted. As a result, we regard **3A**-I, **3B**-I, **4A**-I, and **4B**-I as the most preferable optimized structures for **3a**, **3b**, **4a**, and **4b**, although the absolute configurations of the tBp groups remain unclear.

In addition, the redox properties of carbosilylated Lu_3_N@*I_h_*-C_80_ are consistent with the calculated energies of the highest occupied molecular orbital (HOMO) and LUMO levels of the optimized structures. As shown in Table 2, the HOMOs of **3A**-I, **3B**-I, **4A**-I, and **4B**-I are higher within the range of 0.45–0.53 eV compared with that of pristine Lu_3_N@*I_h_*-C_80_. In contrast, the LUMO energies of **3A**-I, **3B**-I, **4A**-I, and **4B**-I are almost the same as that of pristine Lu_3_N@*I_h_*-C_80_. These results are fully consistent with the experimental redox properties of **3a**, **3b**, **4a**, and **4b**. Therefore, the carbosilylated structures were verified given the electron-donating properties of the silyl groups.

## 3. Materials and Methods

### 3.1. General

All chemicals were reagent grade, purchased from Wako Pure Chemical Industries Ltd (Osaka, Japan). Lu_3_N@*I_h_*-C_80_ was purchased from Luna Innovations Inc. (Danville, CA, USA). 1,2-dichlorobenzene (ODCB) was distilled from P_2_O_5_ under vacuum before use. Toluene was distilled from benzophenone sodium ketyl under dry N_2_ prior to use. Reagents were used as purchased unless otherwise specified. HPLC was performed on an LC-908 apparatus (Japan Analytical Industry Co. Ltd., Tokyo, Japan) monitored using a UV3702 detector. Analytical HPLC was performed on a PU-1586 pump with a UV-1575 detector (JASCO Corp., Tokyo, Japan). Buckyprep (i.d. 20 mm × 250 mm, 4.6 mm × 250 mm), Buckyprep-M (i.d. 10 mm × 250 mm, 4.6 mm × 250 mm), and 5PBB (i.d. 10 mm × 250 mm, 4.6 mm × 250 mm) columns (Nacalai Tesque Inc., Kyoto, Japan) were used for HPLC separation. Toluene was used as the eluent for HPLC. The ^1^H and ^13^C-NMR measurements were conducted on a JEOL ECA-500 spectrometer (JEOL Ltd., Tokyo, Japan). MALDI-TOF mass experiments were performed (Autoflex Ш Smartbeam, Bruker Daltonics, Billerica, MA, USA) with 1,1,4,4-tetraphenyl-1,3-butadiene (TPB) as the matrix in both positive and negative ion modes. Absorption spectra were measured using a UV spectrophotometer (UV-3150, Shimadzu Corp., Kyoto, Japan). Cyclic voltammograms and differential pulse voltammograms were recorded on an electrochemical analyzer (BAS CV50W, BAS Inc., Tokyo, Japan). The reference electrode was a saturated calomel reference electrode (SCE). The glassy carbon electrode was used as the working electrode, and a platinum wire was used as the counter electrode. All potentials are referenced to the ferrocene/ferrocenium couple (Fc/Fc^+^) as the standard. (*n*-Bu)_4_NPF_6_ (0.1 M) in ODCB was used as the supporting electrolyte solution.

### 3.2. Photoreaction of Lu_3_N@I_h_-C_80_ with ***1***

A Pyrex tube (7 mm i.d.) containing Lu_3_N@*I_h_*-C_80_ (0.7 mg, 4.7 × 10^−4^ mmol), **1** (11.5 mg, 2.3 × 10^−2^ mmol), and toluene (3.0 mL) was prepared and degassed using freeze-pump-thaw cycles under reduced pressure. Subsequently, the solution was irradiated for 60 h with a 500 W halogen lamp using an aqueous sodium nitrite filter solution (cutoff < 400 nm) under an argon atmosphere. The resulting reaction mixtures were separated using preparative HPLC with Buckyprep-M, Buckyprep, and 5PBB columns to isolate **3a**, **3b**, and **3c**.

**3a**: ^1^H-NMR (CD_2_Cl_2_) δ 7.49–7.32 (m, 4H), 7.28 (s, 1H), 7.04 (s, 1H), 7.02 (s, 1H), 6.95 (s, 1H), 3.66 (dd, 1H, *J* = 2.5 Hz, 15.0 Hz), 3.00 (s, 3H), 2.85 (dd, 1H, *J* = 13.5 Hz, 15.0 Hz), 2.73 (s, 3H), 2.43 (s, 3H), 2.11 (s, 3H), 1.84 (dd, 1H, *J* = 2.5 Hz, 13.5 Hz), 1.40 (s, 9H), 1.31 (s, 9H), 1.23 (s, 9H); ^13^C-NMR (CDCl_3_:CS_2_ = 1:1) δ 156.88 (1C), 156.79 (1C), 155.84 (1C), 155.47 (1C), 153.73 (1C), 153.24 (1C), 153.11 (1C), 152.31 (1C), 151.08 (1C), 150.93 (1C), 148.85 (1C), 148.65 (1C), 148.34 (1C), 147.88 (1C), 147.56 (1C), 147.22 (2C), 146,66 (1C), 146.38 (1C), 144.88 (1C), 144.64 (1C), 144.34 (1C), 144.29 (1C), 144.20 (1C), 144.01 (1C), 143.57 (1C), 142.97 (1C), 142.58 (1C), 142.35 (1C), 142.32 (1C), 142.26 (1C), 142.13 (2C), 142.06(1C), 141.90 (1C), 141.78 (1C), 141.64 (2C), 141.38 (1C), 141.03 (1C), 140.73 (2C), 140.59 (2C), 140.54 (1C), 140.28 (1C), 139.69 (1C), 139.53 (1C), 139.13 (1C), 138.95 (1C), 138.71 (1C), 138.68 (1C), 138.43 (1C), 138.20 (1C), 137.78 (1C), 137.71 (1C), 137.20 (1C), 137.11 (1C), 137.00 (1C), 136.82 (1C), 136.68 (1C), 136.37 (2C), 136.23 (1C), 136.15 (2C), 135.30 (1C), 135.24 (1C), 135.08 (1C), 135.04 (1C), 134.78 (1C), 134.57 (1C), 134.25 (1C), 134.07 (1C), 133.33 (1C), 132.27 (1C), 131.24 (1C), 130.39 (1C), 129.75 (1C), 129.70 (1C), 129.61 (1C), 126.85 (1C), 126.33 (1C), 126.07 (1C), 125.19 (1C), 124.98 (4C), 118.55 (1C), 109.94 (1C), 108.55 (1C), 103.31 (1C), 63.46 (1C), 59.36 (1C), 54.07 (1C), 34.62 (1C), 34.57 (1C), 34.23 (1C), 31.62 (3C), 31.46 (3C), 31.24 (3C), 30.13 (1C), 26.74 (1C), 26.05 (1C), 23.87 (1C), 20.70 (1C); vis-NIR (CS_2_) λ_max_ 874 nm; MALDI-TOF MS *m/z* 2009 (*M*^−^), 1499 (Lu_3_N@*I_h_*-C_80_^−^).

**3b**: ^1^H-NMR (CD_2_Cl_2_:CS_2_ = 1:1) δ 7.40 (d, 2H, *J* = 7.5 Hz), 7.29 (d, 2H, *J* = 7.5 Hz), 7.25 (s, 1H), 6.95 (s, 1H), 6.82 (s, 1H), 6.75 (s, 1H), 3.66 (dd, 1H, *J* = 2.5 Hz, 15.0 Hz), 3.38 (s, 3H), 2.59 (dd, 1H, *J* = 13.5 Hz, 15.0 Hz), 2.58 (s, 3H), 2.50 (s, 3H), 2.29 (s, 3H), 1.74 (dd, 1H, *J* = 2.5 Hz, 13.5 Hz), 1.36 (s, 9H), 1.30 (s, 9H), 1.17 (s, 9H); vis-NIR (CS_2_) λ_max_ 879 nm; MALDI-TOF MS *m*/*z* 2009 (*M*^−^), 1499 (Lu_3_N@*I_h_*-C_80_^−^). 

**3c**: vis-NIR (CS_2_) λ_max_ 811 nm; MALDI-TOF MS *m*/*z* 2009 (*M*^−^), 1499 (Lu_3_N@*I_h_*-C_80_^−^).

### 3.3. Photoreaction of Lu_3_N@I_h_-C_80_ with ***2***

A Pyrex tube (20 mm i.d.) containing Lu_3_N@*I_h_*-C_80_ (1.1 mg, 7.3 × 10^−4^ mmol), **2** (49.4 mg, 1.1 × 10^−1^ mmol), and toluene (20 mL) was prepared and degassed using freeze-pump-thaw cycles under reduced pressure. These solutions were irradiated for 20 h under the same conditions. The reaction mixture was separated using preparative HPLC with Buckyprep-M and Buckyprep columns to isolate **4a**, **4b**, and **4c**.

**4a**: ^1^H-NMR (CDCl_3_:CS_2_ = 1:1) δ 7.50 (t, 1H, *J* = 7.5 Hz), 7.47–7.42 (m, 2H), 7.40 (d, 1H, *J* = 7.5 Hz), 7.38–7.34 (m, 2H), 7.23 (t, 1H, *J* = 7.5 Hz), 7.12 (d, 1H, *J* = 7.5 Hz), 7.05 (d, 1H, *J* = 7.5 Hz), 6.99 (d, 1H, *J* = 7.5 Hz), 3.69 (dd, 1H, *J* = 2.5 Hz, 15.5 Hz), 3.51 (dq, 1H, *J* = 7.5 Hz, 15.0 Hz), 3.44 (dq, 1H, *J* = 7.5 Hz, 15.0 Hz), 3.14 (dq, 1H, *J* = 7.5 Hz, 15.0 Hz), 3.03 (dq, 1H, *J* = 7.5 Hz, 15.0 Hz), 2.89 (dd, 1H, *J* = 13.5 Hz, 15.5 Hz), 2.84 (dq, 1H, *J* = 7.5 Hz, 15.0 Hz), 2.76 (dq, 1H, *J* = 7.5 Hz, 15.0 Hz), 2.53 (dq, 1H, *J* = 7.5 Hz, 15.0 Hz), 2.52 (dq, 1H, *J* = 7.5 Hz, 15.0 Hz), 1.84 (dd, 1H, *J* = 2.5 Hz, 13.5 Hz), 1.66 (t, 3H, *J* = 7.5 Hz), 1.34 (s, 9H), 1.22 (t, 3H, *J* = 7.5 Hz), 0.64 (t, 3H, *J* = 7.5 Hz), 0.31 (t, 3H, *J* = 7.5 Hz); ^13^C-NMR (CDCl_3_) *δ* 157.35 (1C), 156.99 (1C), 156.81 (1C), 155.88 (1C), 155.56 (1C), 155.53 (1C), 154.44 (1C), 154.39 (1C), 153.31 (1C), 153.14 (1C), 151.45 (1C), 151.29 (1C), 150.51 (1C), 149.14 (1C), 148.84 (1C), 148.67 (1C), 148.17 (1C), 147.93 (1C), 147.48 (1C), 147.35 (1C), 147.14 (1C), 146.85 (1C), 146.66 (1C), 146.38 (1C), 144.81 (1C), 144.76 (1C), 144.63 (1C), 144.42 (1C), 144.32 (1C), 144.23 (1C), 143.90 (1C), 142.90 (1C), 142.64 (1C), 142.31 (1C), 142.19 (1C), 142.12 (1C), 141.99 (1C), 141.82 (1C), 141.42 (1C), 140.94 (1C), 140.85 (1C), 140.64 (1C), 140.34 (1C), 139.57 (1C), 139.43 (1C), 139.34 (1C), 138.79 (1C), 138.73 (1C), 138.49 (1C), 138.02 (1C), 137.63 (1C), 137.06 (1C), 137.00 (1C), 136.72 (1C), 136.41 (1C), 136.27 (1C), 136.15 (1C), 136.02 (1C), 135.31 (1C), 135.23 (1C), 135.17 (1C), 135.09 (1C), 135.05 (1C), 134.84 (1C), 134.59 (1C), 134.13 (1C), 131.56 (1C), 130.99 (1C), 130.64 (1C), 130.26 (1C), 129.84 (1C), 129.45 (1C), 129.14 (1C), 128.90 (1C), 128.34 (1C), 127.81 (1C), 127.29 (1C), 127.17 (1C), 126.03 (1C), 125.34 (1C), 124.90 (2C), 118.00 (1C), 110.10 (1C), 109.34 (1C), 103.64 (1C), 63.34 (1C), 58.45 (1C), 55.24 (1C), 34.68 (1C), 34.15 (1C), 33.56 (1C), 31.55 (1C), 30.27 (1C), 29.53 (1C), 21.62 (1C), 15.19 (1C), 15.09 (1C), 15.01 (1C), 14.83 (1C); vis-NIR (CS_2_) λ_max_ 879 nm; MALDI-TOF MS *m*/*z* 1952 (*M*^−^), 1499 (Lu_3_N@*I*_h_-C_80_^−^).

**4b**: ^1^H-NMR (CDCl_3_:CS_2_ = 1:1) δ 7.32 (d, 2H, *J* = 8.0 Hz), 7.37 (d, 2H, *J* = 4.5 Hz), 7.31 (d, 2H, *J* = 8.0 Hz), 7.10 (t, 1H, *J* = 7.5 Hz), 7.02 (t, 1H, *J* = 4.5 Hz), 6.92 (d, 1H, *J* = 7.5 Hz), 6.86 (d, 1H, *J* = 7.5 Hz), 3.79 (dd, 1H, *J* = 2.5 Hz, 15.5 Hz), 3.74 (q, 2H, *J* = 7.5 Hz), 3.29 (dq, 1H, *J* = 7.5 Hz, 15.0 Hz), 3.02 (dq, 1H, *J* = 7.5 Hz, 15.0 Hz), 2.99 (dq, 1H, *J* = 7.5 Hz, 15.0 Hz), 2.84 (dq, 1H, *J* = 7.5 Hz, 15.0 Hz), 2.72 (dq, 1H, *J* = 7.5 Hz, 15.0 Hz), 2.69 (dq, 1H, *J* = 7.5 Hz, 15.0 Hz), 2.64 (dd, 1H, *J* = 13.5 Hz, 15.5 Hz), 1.84 (t, 3H, *J* = 7.5 Hz), 1.75 (dd, 1H, *J* = 2.5 Hz, 13.5 Hz), 1.26 (s, 9H), 1.21 (t, 3H, *J* = 7.5 Hz), 0.56 (t, 3H, *J* = 7.5 Hz), 0.55 (t, 3H, *J* = 7.5 Hz); vis-NIR (CS_2_) λ_max_ 882 nm; MALDI-TOF MS *m/z* 1952 (*M*^−^), 1499 (Lu_3_N@*I_h_*-C_80_^−^).

**4c**: vis-NIR (CS_2_) λ_max_ 803 nm; MALDI-TOF MS *m/z* 1952 (*M*^−^), 1499 (Lu_3_N@*I_h_*-C_80_^−^).

### 3.4. Computational Method

All calculations were conducted using the Gaussian09 [29] program. The optimized geometries were calculated at the B3LYP [26,27,28] level of theory using basis sets of 6-31G(d) [30] for C, H, N, Si atoms, and SDD [31] for Lu atoms.

## 4. Conclusions

In summary, the photochemical addition of siliranes **1** and **2** to Lu_3_N@*I_h_*-C_80_ afforded the corresponding [5,6]- and [6,6]-adducts. The [5,6]-adducts **3a**, **3b**, **4a**, and **4b** were characterized on the basis of spectroscopic and electrochemical studies and theoretical calculations. The electron-donating effect of carbosilylation on Lu_3_N@*I_h_*-C_80_ was confirmed by the redox properties of **3a**, **3b**, **4a**, and **4b**, which showed remarkably low first oxidation potentials. The carbosilylation also resulted in cathodic shifts of the first reduction potentials compared to those of the [5,6]-pyrrolidino adducts. Such functional groups with various electronic effects will contribute to the utilization of EMFs for future applications. Further studies of novel functionalizing methods based on organosilanes are now underway.

## Figures and Tables

**Table 1:** Redox potentials (V) ^a^ of **3a**, **3b**, **4a**, **4b**, and related compounds.

compound	*E* ^ox^_1_	*E* ^red^_1_	*E* ^red^_2_
Lu_3_N@*I_h_*-C_80_^b^	+0.61	–1.39	–1.83
**3a**	+0.23 ^c^	–1.38	–1.73
**3b**	+0.25 ^c^	–1.35	–1.71
**4a**	+0.22 ^c^	–1.37	–1.75
**4b**	+0.21 ^c^	–1.41	–1.77
**5** ^d^		–1.13	–1.64
**6** ^e^		–1.14	

^a^ Values obtained by DPV are in volts relative to the ferrocene/ferrocenium couple. ^b^ Data from ref. [18]. ^c^ Irreversible. ^d^ Data from ref. [23]. ^e^ Data from ref. [24].

**Table 2:** Calculated HOMO/LUMO levels (eV) of Lu_3_N@*I_h_*-C_80_, **3A**-I, **3B**-I, **4A**-I, and **4B**-I.

compound	HOMO	LUMO
Lu_3_N@*I_h_*-C_80_	–5.47	–2.90
**3A**-I	–4.98	–2.90
**3B**-I	–4.99	–2.91
**4A**-I	–5.01	–2.92
**4B**-I	–5.02	–2.95

## Supplementary Materials

Supplementary materials are available online.
