# Supplementary material for: Photoreactions of Endohedral Metallofullerene with Siliranes: Electronic Properties of Carbosilylated Lu3N@Ih-C80
Source: Molecules. 2017 May 20;22(5):850. doi: 10.3390/molecules22050850 (PMC6154294; doi:10.3390/molecules22050850)
Supplement: Supplementary file 1 [file molecules-22-00850-s001.pdf]

Supporting Information

# Photoreactions of Endohedral Metallofullerene with Siliranes: Electronic Properties of Carbosilylated $Lu_3N@I_h$-$C_{80}$

**Masahiro Kako[1,*], Kazuya Minami[1], Taiki Kuroiwa[1], Shinpei Fukazawa[1], Yuki Arikawa[1], Michio Yamada[2], Yutaka Maeda[2], Qiao-Zhi Li[3], Shigeru Nagase[3,*] and Takeshi Akasaka[2,4,5,6,*]**

[1] Department of Engineering Science, The University of Electro-Communications, Chofu 182-8585, Japan

[2] Department of Chemistry, Tokyo Gakugei University, Tokyo 184-8501, Japan

[3] Fukui Institute for Fundamental Chemistry, Kyoto University, Kyoto 606-8103, Japan

[4] Life Science Center of Tsukuba Advanced Research Alliance, University of Tsukuba, Ibaraki 305-8577, Japan

[5] Foundation for Advancement of International Science, Ibaraki 305-0821, Japan

[6] School of Materials Science and Engineering, Huazhong University of Science and Technology, Wuhan 430074, China

## Table of Contents

**Figure S1.** HPLC profiles of the separation of **3a**, **3b**, and **3c**. SI2
**Figure S2.** HPLC profiles of the separation of **4a**, **4b**, and **4c**. SI2
**Figure S3.** Negative-mode MALDI-TOF mass spectrum of **3a**, **3b**, and **3c**. SI3
**Figure S4.** Negative-mode MALDI-TOF mass spectrum of **4a**, **4b**, and **4c**. SI4
**Figure S5.** 500 MHz $^1$H NMR of **3a**. SI5
**Figure S6.** 500 MHz $^1$H NMR of **3b**. SI5
**Figure S7.** 125 MHz $^{13}$C NMR spectra of **3a**. SI6
**Figure S8.** 500 MHz $^1$H NMR of **4a**. SI7
**Figure S9.** 500 MHz $^1$H NMR of **4b**. SI7
**Figure S10.** 125 MHz $^{13}$C NMR spectra of **4a**. SI8
**Figure S11.** CV and DPV of **3a** and **3b**. SI9
**Figure S12.** CV and DPV of **4a** and **4b**. SI10
**Figure S13.** Optimized structures of **3A**-I, **3A**-II, and **3A**-III. SI11
**Figure S14.** Optimized structures of **3B**-I, **3B**-II, and **3B**-III. SI12
**Figure S15.** Optimized structures of **4A**-I, **4A**-II, and **4A**-III. SI13
**Figure S16.** Optimized structures of **4B**-I, **4B**-II, and **4B**-III. SI14
**Table S1.** Cartesian coordinates of optimized structures. SI15

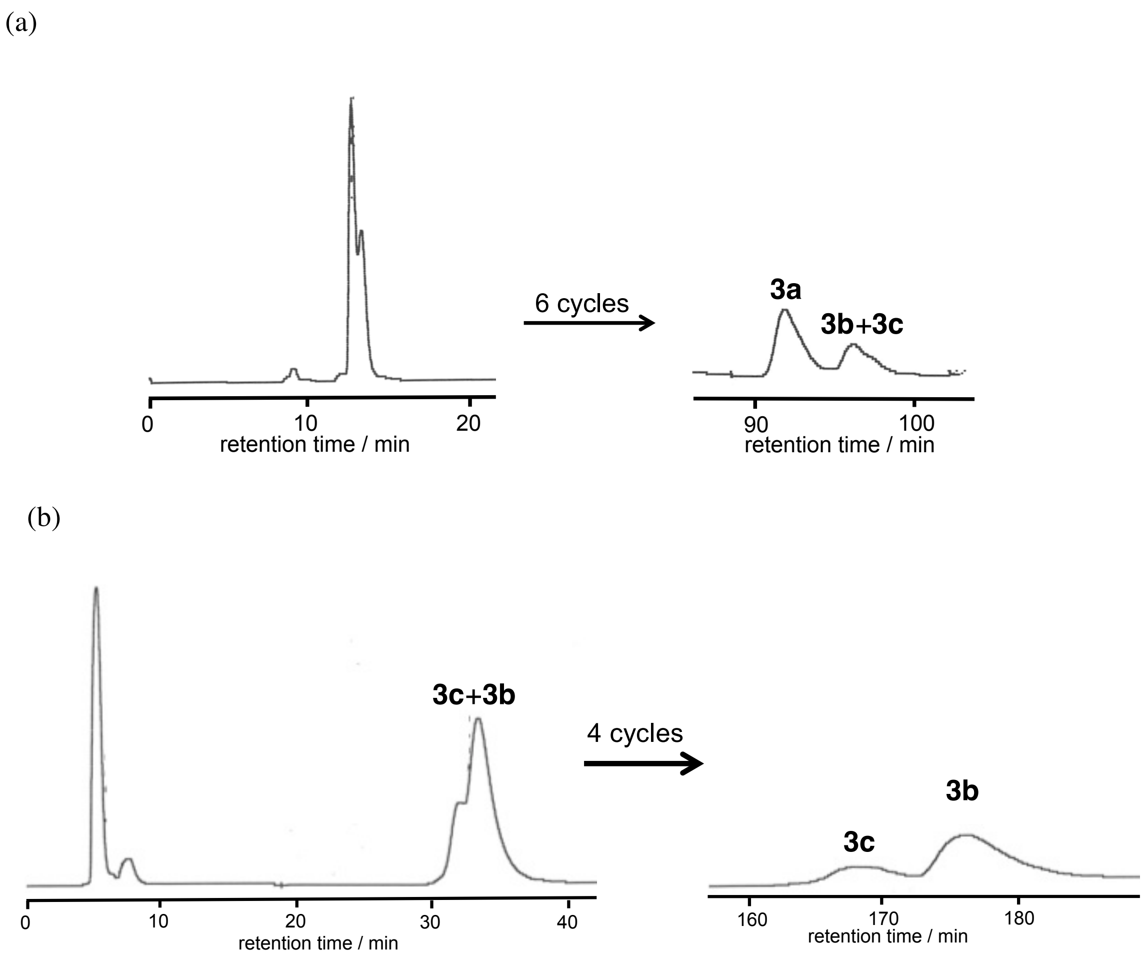


**Figure S1.** HPLC profiles of the separation of **3a**, **3b**, and **3c**. HPLC conditions: (a) Buckyprep (ϕ 20 × 250 mm); eluent, toluene; flow rate, 6.5 mL/min; detection wavelength, 282 nm; temperature, room temperature. (b) 5PBB (ϕ 10 × 250 mm); eluent, toluene; flow rate, 3.0 mL/min; detection wavelength, 282 nm; temperature, room temperature.

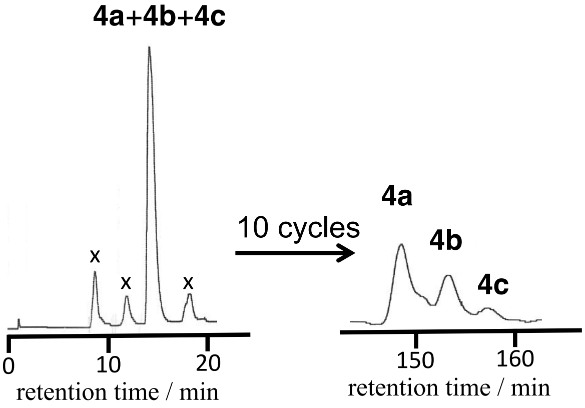


**Figure S2.** HPLC profiles of the separation of **4a**, **4b**, and **4c**. HPLC conditions: Buckyprep (ϕ 20 × 250 mm); eluent, toluene; flow rate, 6.5 mL/min; detection wavelength, 330 nm; temperature, room temperature.

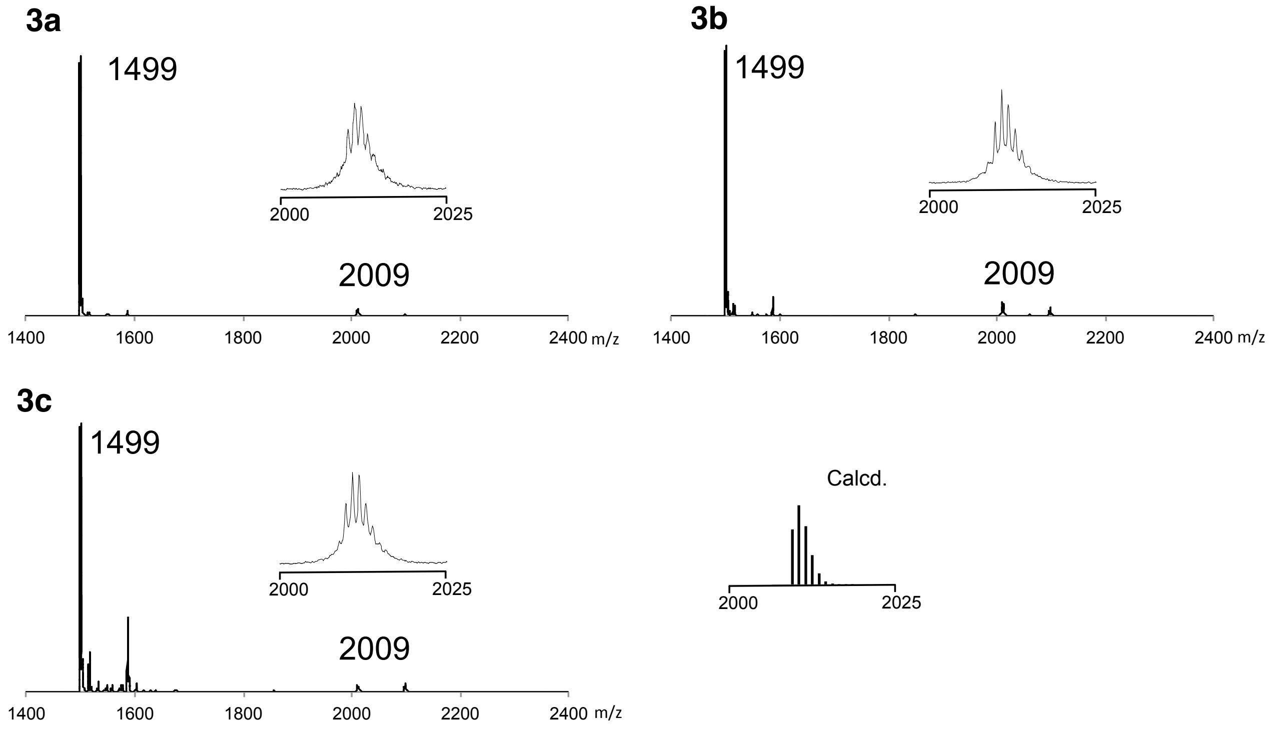


**Figure S3.** Negative-mode MALDI-TOF mass spectrum of **3a**, **3b**, and **3c**, and the simulated isotope pattern of the parent peak. Matrix: 1,1,4,4-tetraphenyl-1,3-butadiene.

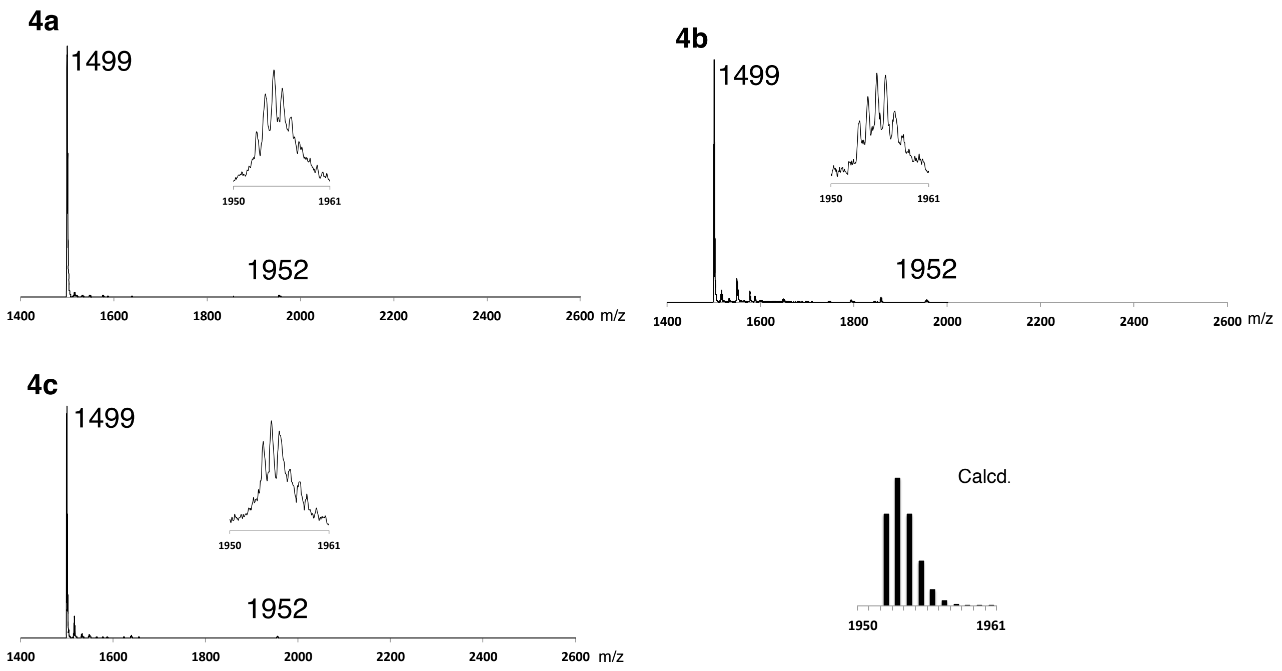


**Figure S4.** Negative-mode MALDI-TOF mass spectrum of **4a**, **4b**, and **4c**, and the simulated isotope pattern of the parent peak. Matrix: 1,1,4,4-tetraphenyl-1,3-butadiene.

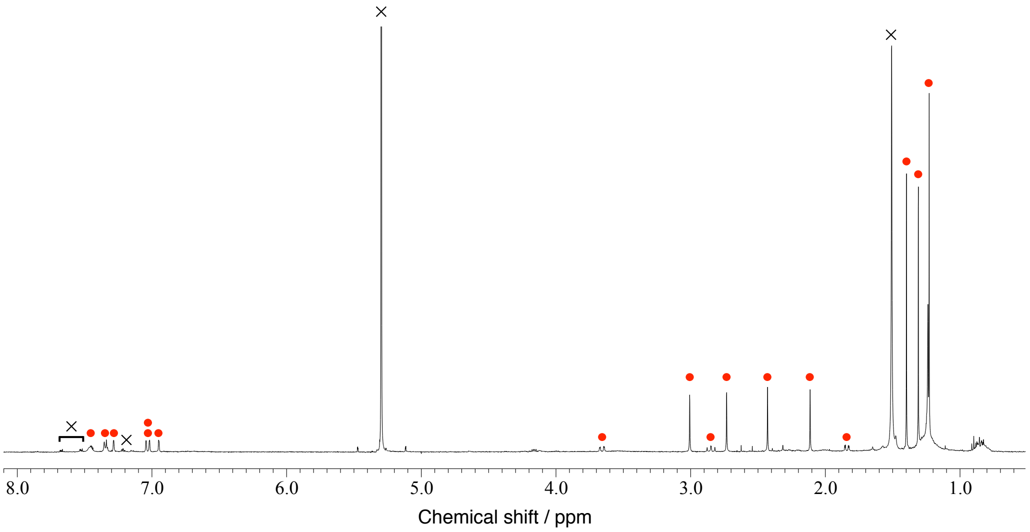


**Figure S5.** 500 MHz $^{1}$H NMR of **3a** recorded at 293 K in $CD_2Cl_2$.

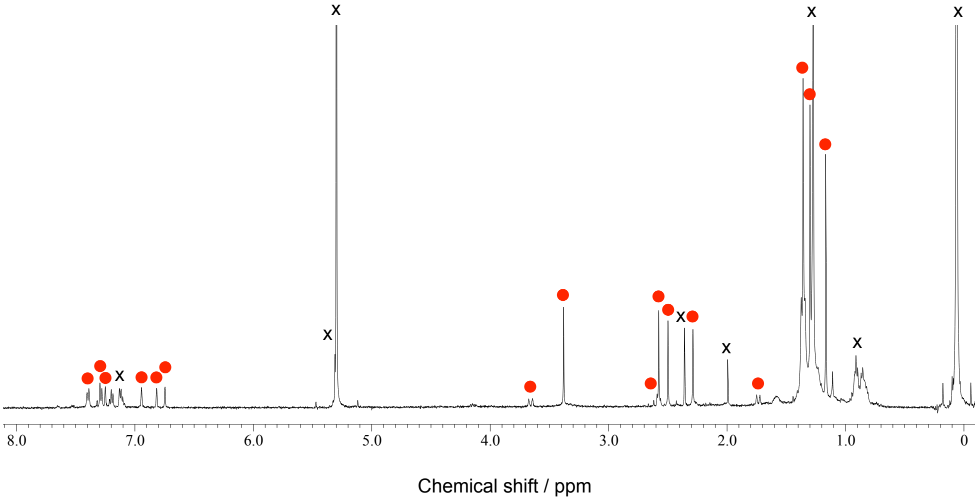


**Figure S6.** 500 MHz $^{1}$H NMR of **3b** recorded at 293 K in $CD_2Cl_2$ / $CS_2$(1:1).

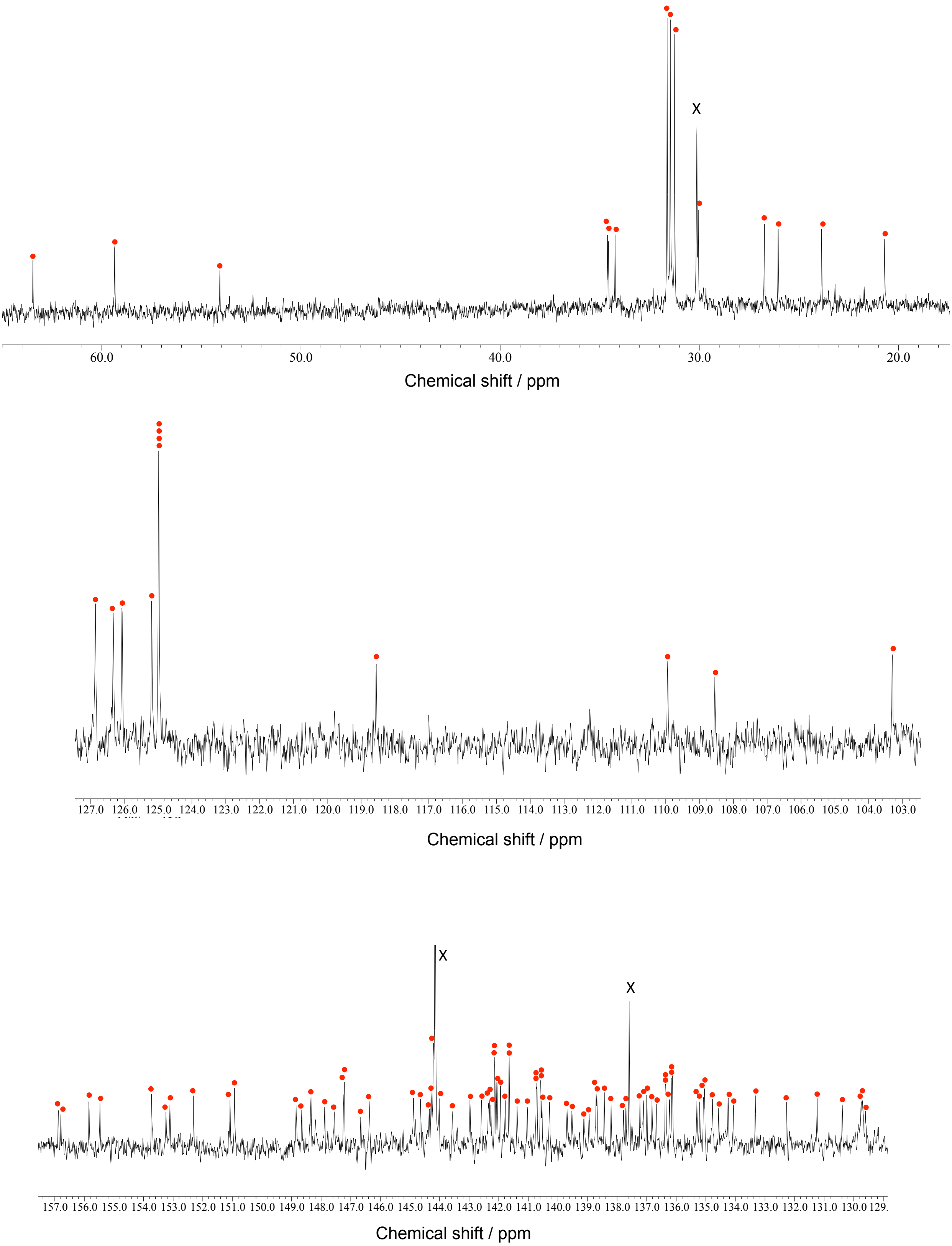


**Figure S7.** 125 MHz $^{13}C$ NMR spectra of **3a** recorded at 293 K in $CDCl_3$ /$CS_2$(1:1).

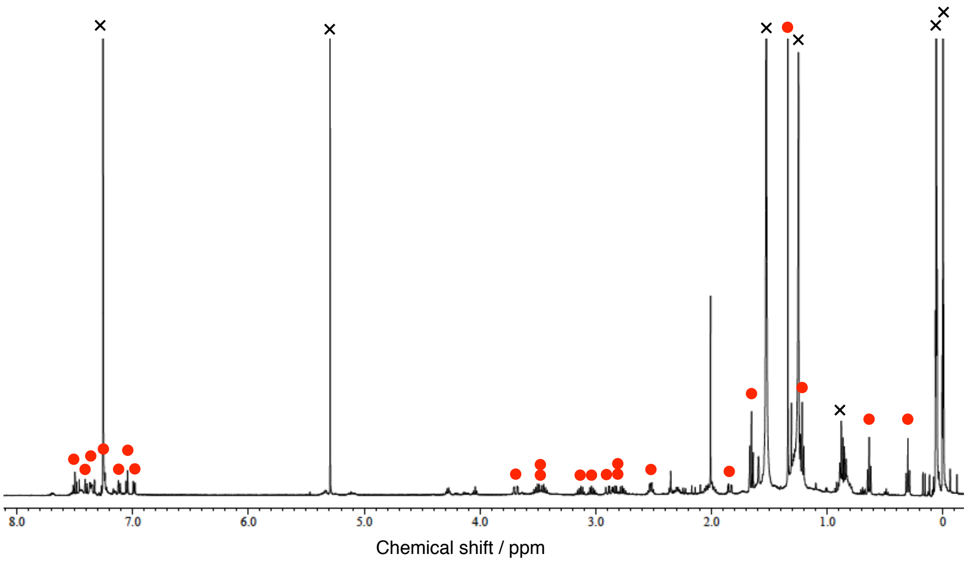


**Figure S8.** 500 MHz $^{1}$H NMR of **4a** recorded at 293 K in $CDCl_3/CS_2$(1:1).

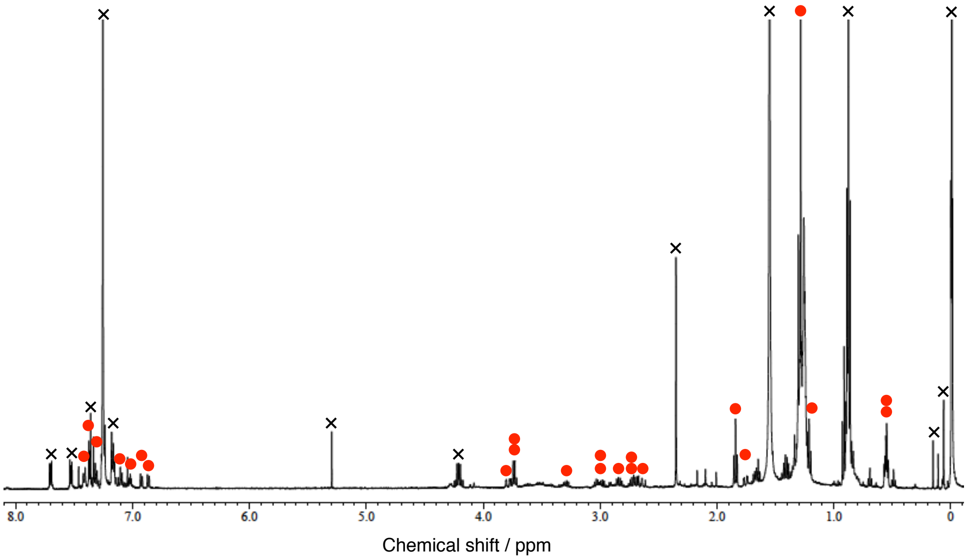


**Figure S9.** 500 MHz $^{1}$H NMR of **4b** recorded at 293 K in $CDCl_3/CS_2$(1:1).

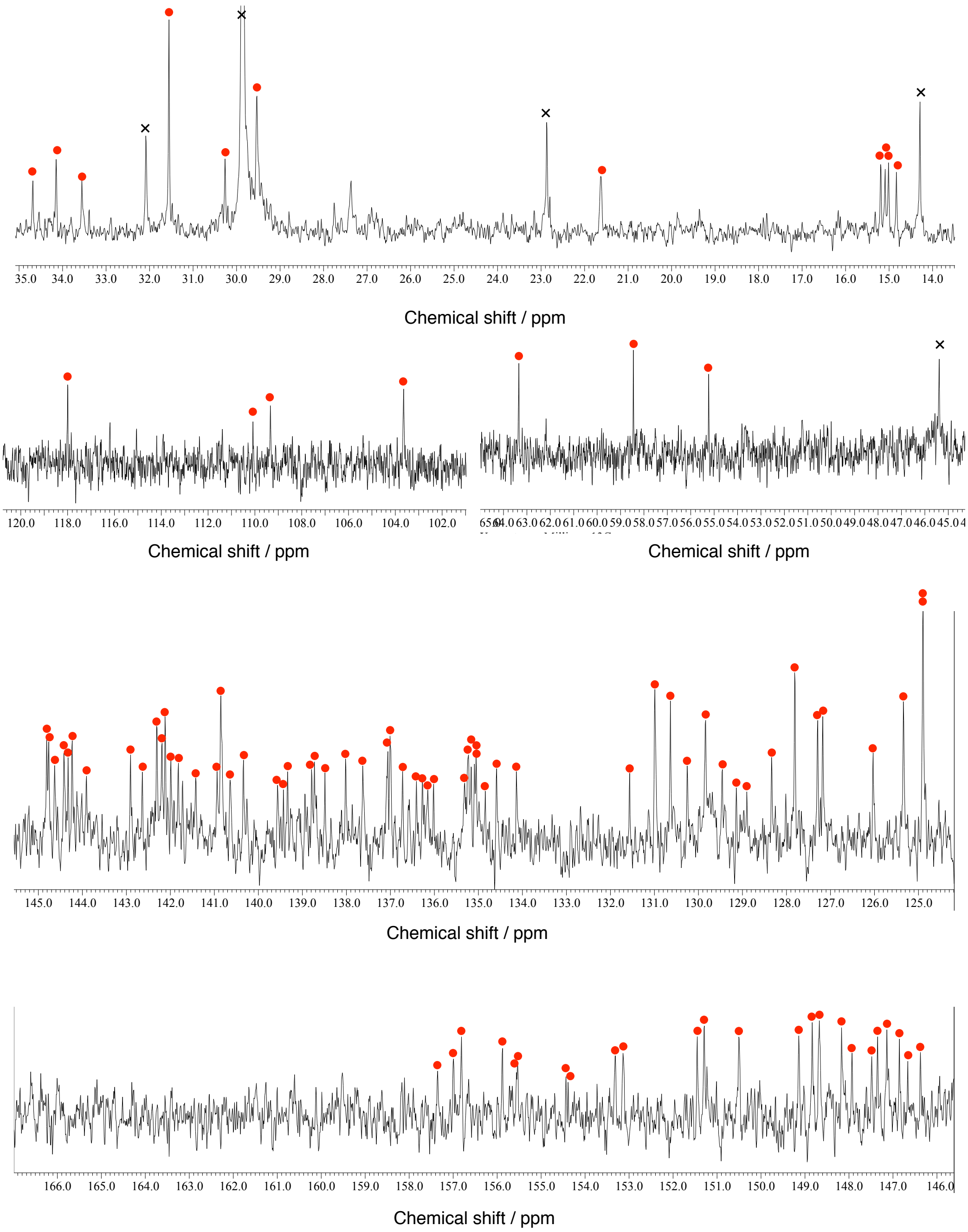


**Figure S10.** 125 MHz $^{13}C$ NMR spectra of **4a** recorded at 293 K in $CDCl_3/CS_2$(1:1).

(a)

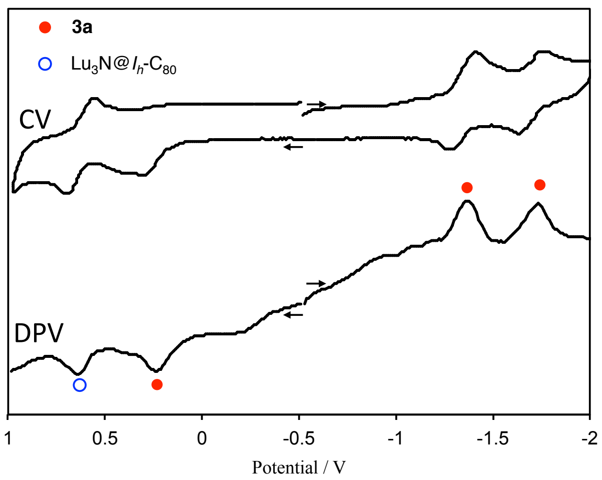


(b)

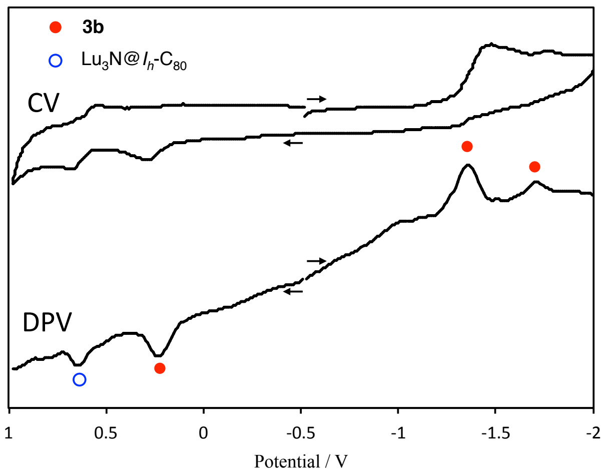


**Figure S11.** Cyclic voltammograms (CV) and differential pulse voltammograms (DPV) of (a) **3a** and (b) **3b** in ODCB containing 0.1 M ($n$-Bu)$_4$NPF$_6$. Conditions: working electrode, a glassy carbon electrode; counter-electrode, Pt wire; reference electrode, SCE; CV scan rates, 20 mV/s; DPV scan rate, 50 mV/s.

(a)

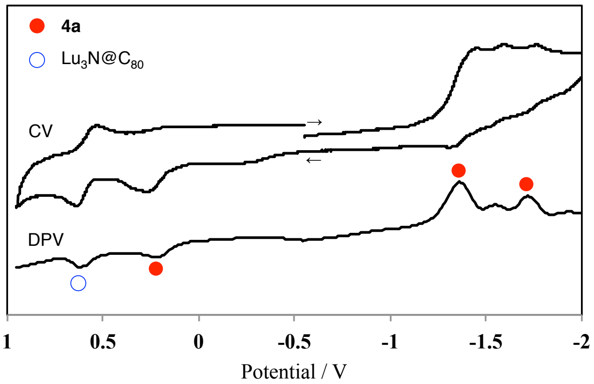


(b)

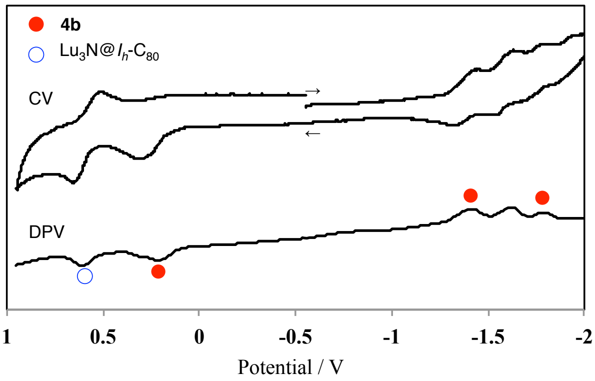


**Figure S12.** Cyclic voltammograms (CV) and differential pulse voltammograms (DPV) of (a) **4a** and (b) **4b** in ODCB containing 0.1 M ($n$-Bu)$_4$NPF$_6$. Conditions: working electrode, a glassy carbon electrode; counter-electrode, Pt wire; reference electrode, SCE; CV scan rates, 20 mV/s; DPV scan rate, 50 mV/s.

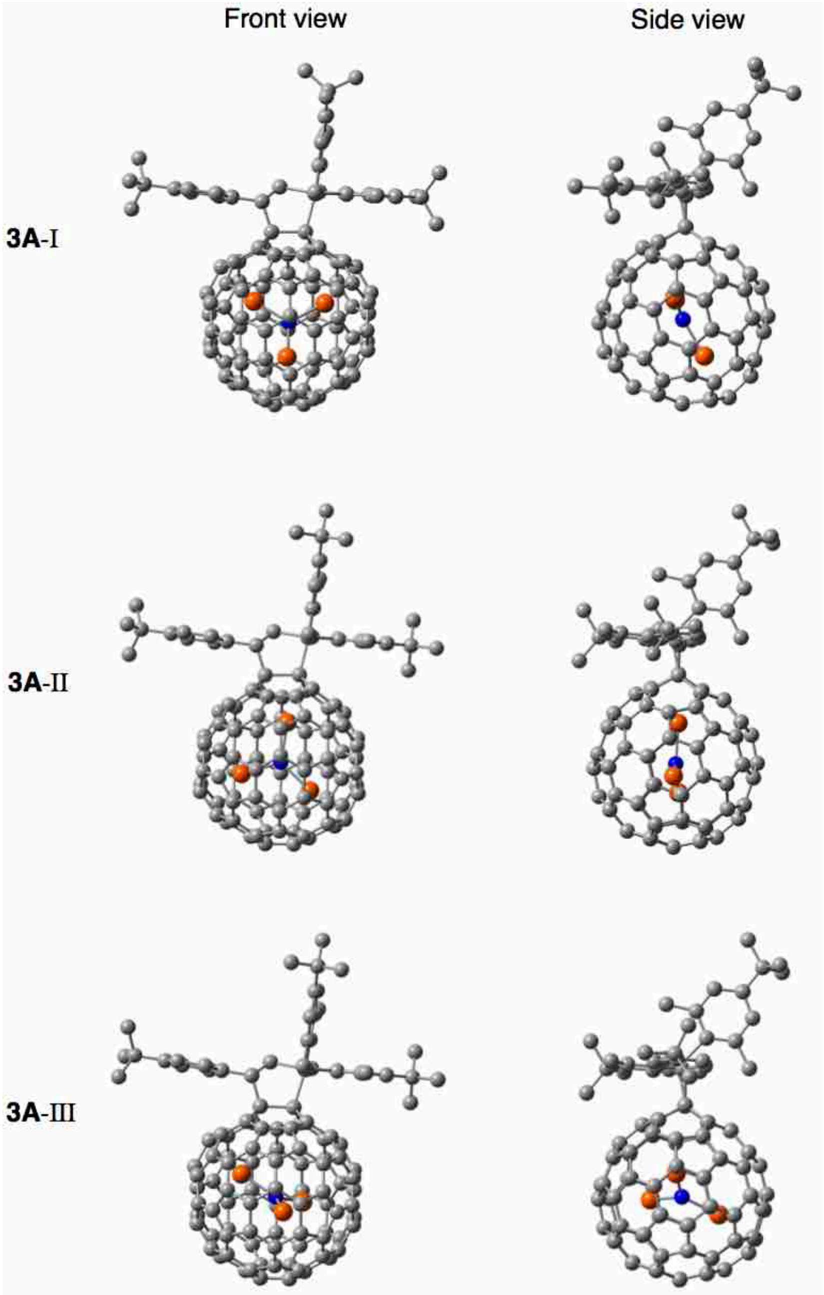


**Figure S13.** Optimized structures of **3A**-I, **3A**-II, and **3A**-III.

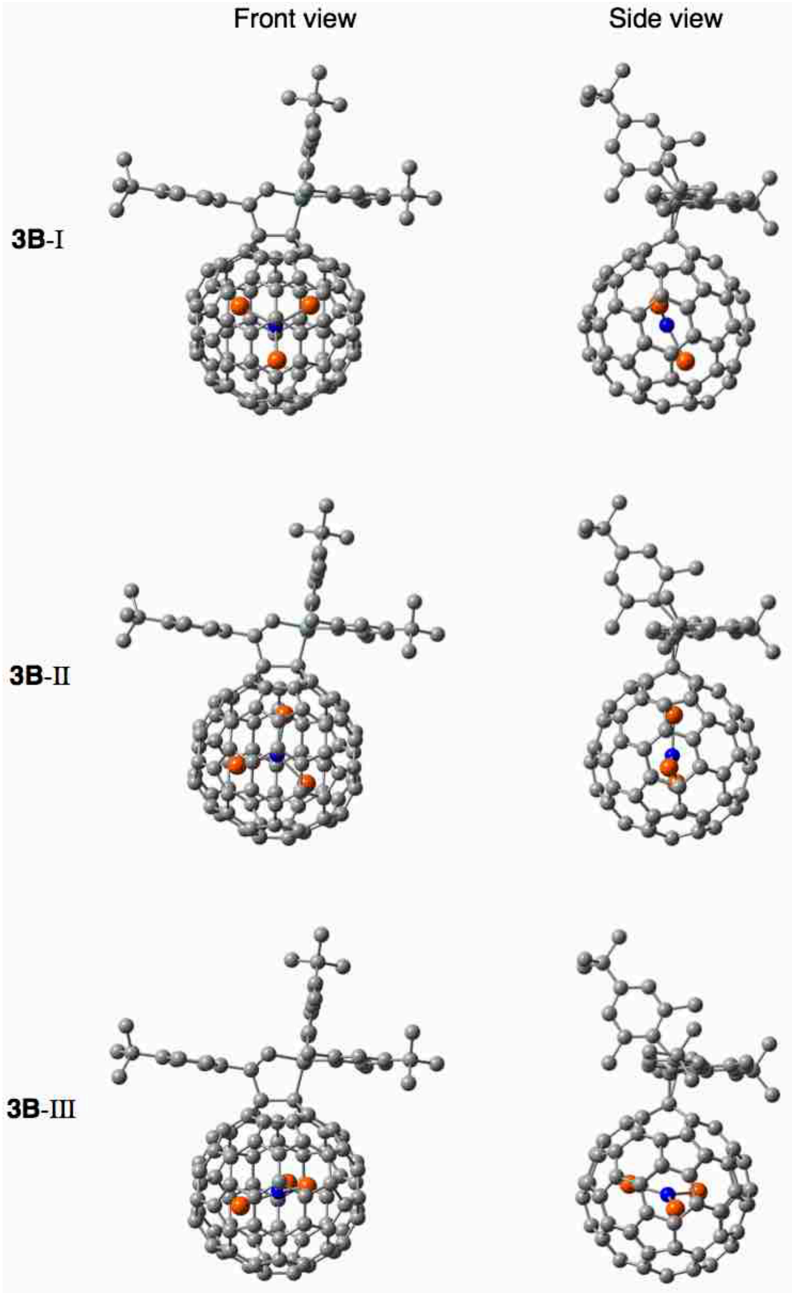


**Figure S14.** Optimized structures of **3B**-I, **3B**-II, and **3B**-III.

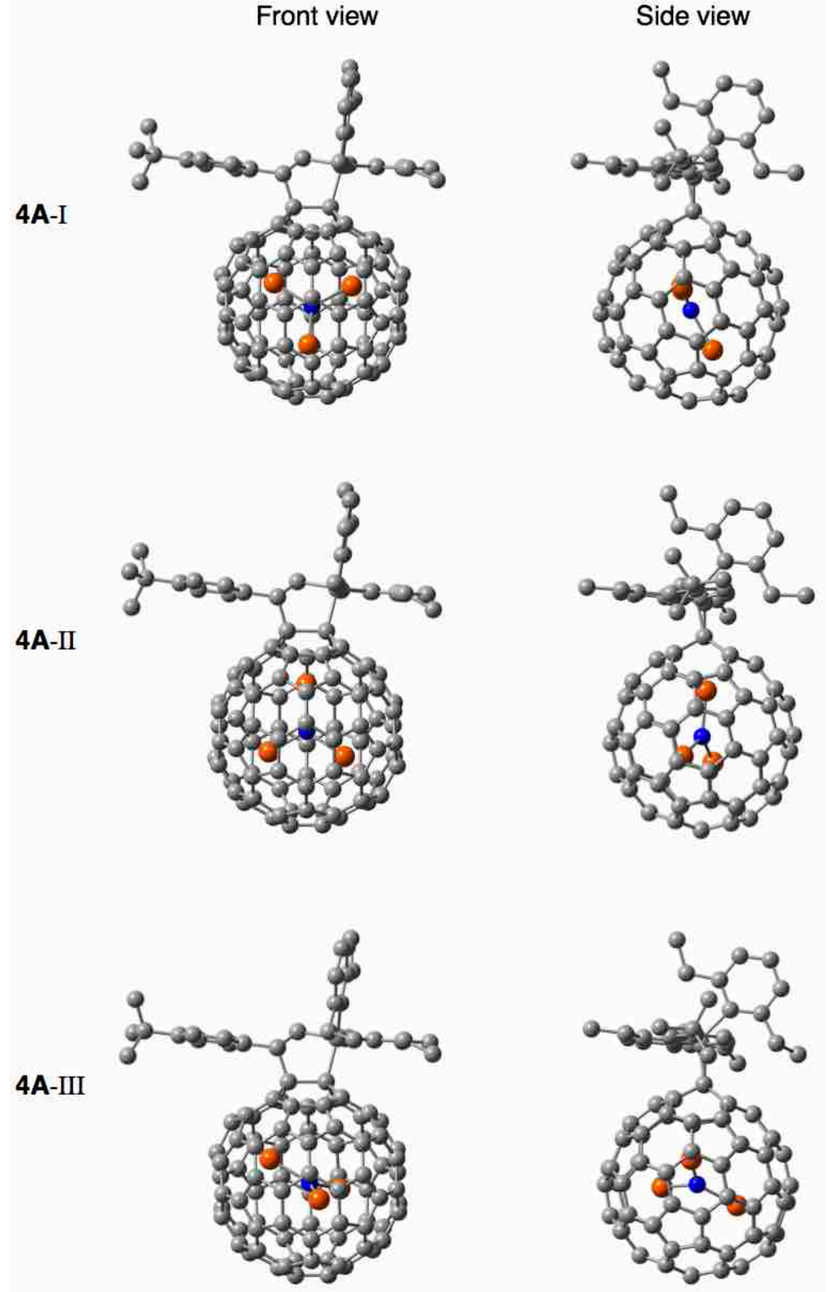


**Figure S15.** Optimized structures of **4A**-I, **4A**-II, and **4A**-III.

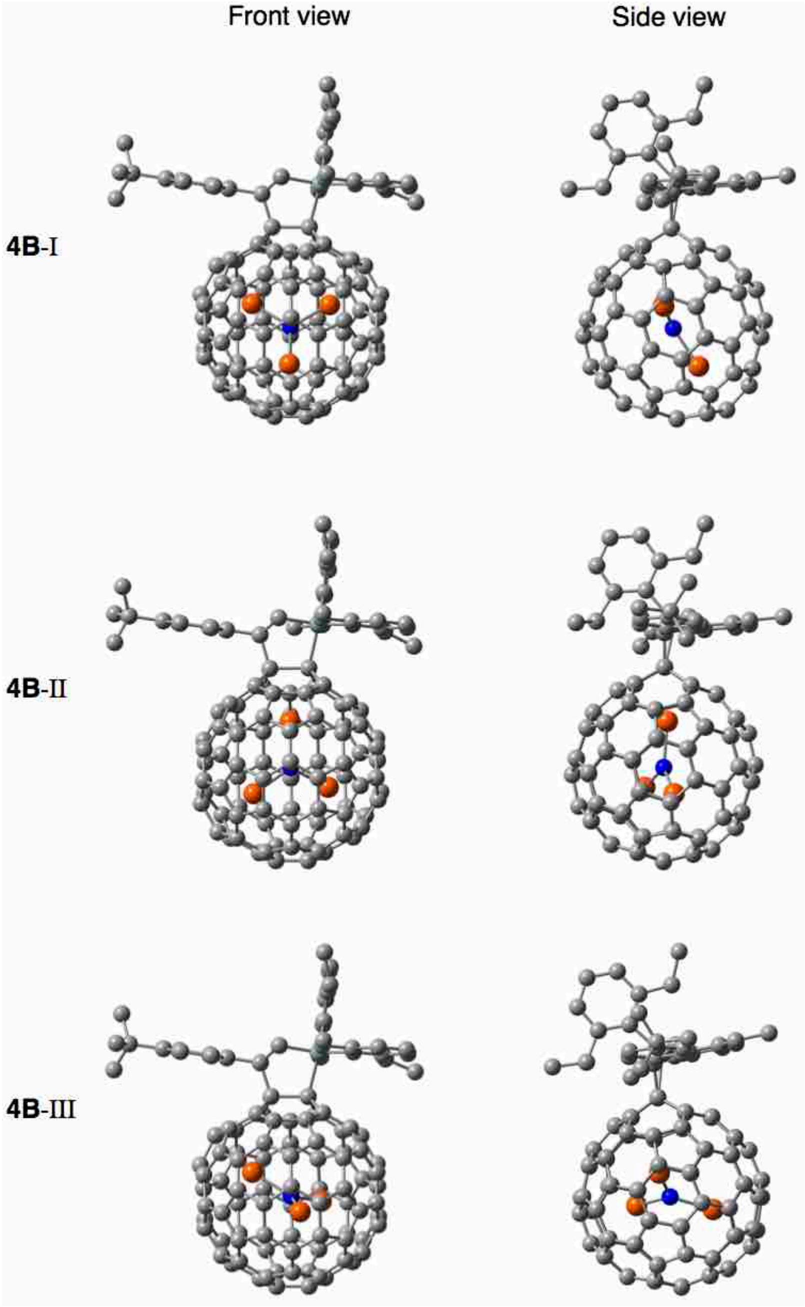


**Figure S16.** Optimized structures of **4B**-I, **4B**-II, and **4B**-III.

**Table S1.** Cartesian coordinates of optimized structures.

**3A**-I

| | | | |
|---|---|---|---|
| C | -2.44222900 | 2.73204800 | 2.98298300 |
| C | -2.84686000 | 1.59785000 | 3.75725300 |
| C | -1.90752600 | 0.57764900 | 4.10625900 |
| C | -0.54703900 | 0.76374200 | 3.72744100 |
| C | -4.22172800 | 1.27438800 | 3.42005900 |
| C | -2.40383700 | -0.75940100 | 4.18654200 |
| C | -3.77729600 | -1.07188900 | 3.83512500 |
| C | -4.75667500 | -0.06862000 | 3.49177900 |
| C | -2.48056500 | -0.97000200 | -3.82105500 |
| C | -1.99498000 | 0.37622300 | -3.88885400 |
| C | -0.64862000 | 0.69655100 | -3.50959100 |
| C | 0.22395600 | -0.32221100 | -3.04173600 |
| C | -0.20965400 | -1.67440500 | -3.07988000 |
| C | -1.55366200 | -1.99976400 | -3.46148400 |
| C | -0.61406600 | 2.04097900 | -2.96835200 |
| C | 1.13779100 | 0.03964100 | -2.00087200 |
| C | 1.22288400 | 1.37963300 | -1.49968100 |
| C | 0.35037100 | 2.42381800 | -1.95543100 |
| C | 0.25577100 | 3.13044300 | 0.40490800 |
| C | 1.15948400 | 2.08583100 | 0.82417300 |
| C | -0.14671300 | 1.89542700 | 2.93870400 |
| C | -1.09047100 | 2.86423600 | 2.52576700 |
| C | -0.87065700 | 3.46464900 | 1.24409200 |
| C | 0.93120900 | 1.49883300 | 2.07082700 |
| C | -5.51166200 | -2.45346000 | -0.56670200 |
| C | -5.34659500 | -1.89566200 | -1.86295300 |
| C | -4.26292000 | -2.30720100 | -2.71360600 |
| C | -3.31760800 | -3.28492000 | -2.27729500 |
| C | -3.53621400 | -3.90724700 | -1.01472200 |
| C | -4.62797900 | -3.50354100 | -0.17284000 |
| C | -3.84773900 | -1.16247100 | -3.47818700 |
| C | -1.95806800 | -3.14695000 | -2.70496600 |
| C | -4.21766500 | -3.63662400 | 1.19753600 |
| C | -6.03800400 | -1.59417700 | 0.47282100 |
| C | -5.70652500 | -1.75136200 | 1.87049100 |
| C | -4.68696000 | -2.73524900 | 2.20703400 |
| C | 1.18945200 | 0.07762000 | 2.32462800 |
| C | 0.26820200 | -0.35225400 | 3.34384400 |
| C | -3.53564000 | 3.12459300 | 2.14360100 |
| C | -4.63506400 | 2.22039700 | 2.40146500 |
| C | 0.24938500 | -2.64111500 | -2.10892700 |
| C | -0.84408700 | -3.57055600 | -1.87323000 |
| C | -4.68264200 | -0.04875300 | -3.11977800 |
| C | -5.60856900 | -0.49923000 | -2.11558500 |
| C | -0.24417600 | -1.66978600 | 3.35480800 |
| C | -1.57968300 | -1.87272400 | 3.82748400 |
| C | -1.04657800 | -4.19329800 | -0.58144800 |
| C | -1.95014700 | 3.91900600 | 0.41330100 |
| C | -3.31571100 | 3.67506600 | 0.84216300 |
| C | -4.16999400 | 1.28372200 | -3.10480600 |
| C | 1.20379900 | -2.28264800 | -1.08703200 |
| C | -3.76277500 | -2.39201200 | 3.23647300 |
| C | -2.40904200 | -2.88802600 | 3.24367000 |
| C | -1.92796600 | -3.73207800 | 2.19241300 |
| C | -2.86010400 | -4.12741800 | 1.20026200 |
| C | -2.42603900 | -4.29901700 | -0.17127600 |
| C | 1.69332800 | -0.77295300 | 1.34197900 |
| C | 1.65013300 | -0.92042800 | -1.07861500 |
| C | 1.22763800 | -2.14123500 | 1.36337200 |
| C | 0.25049800 | -2.55288300 | 2.33965100 |
| C | -0.53620200 | -3.62223500 | 1.78796800 |
| C | -0.03234800 | -3.92952400 | 0.45011600 |
| C | 1.09862000 | -3.00145700 | 0.19043800 |
| C | -5.81259900 | -0.43734100 | 2.53314700 |
| C | -6.18069500 | 0.52946900 | 1.46818900 |
| C | -6.31786100 | -0.20333700 | 0.22322100 |
| C | -6.04791500 | 0.37153100 | -1.08089500 |

| | | | |
|---|---|---|---|
| C | -5.57028500 | 1.85350300 | 1.38130900 |
| C | -0.08931800 | 3.44214900 | -0.98213100 |
| C | -1.50151300 | 3.90818700 | -0.97358700 |
| C | -2.47418300 | 3.42762000 | -1.95663500 |
| C | -2.82303000 | 1.49114700 | -3.54320900 |
| C | -1.98265000 | 2.53218900 | -2.97975500 |
| C | -3.83377700 | 3.19573800 | -1.53107300 |
| C | -4.26442400 | 3.36162800 | -0.16139600 |
| C | -5.35908100 | 2.45134800 | 0.09302900 |
| C | -5.60872100 | 1.72878800 | -1.12485300 |
| C | -4.67714400 | 2.18135700 | -2.12120100 |
| N | -2.19372800 | -0.19556200 | 0.12041000 |
| C | 2.34887200 | -0.21331600 | 0.09407900 |
| C | 2.05172700 | 1.40073800 | -0.20704800 |
| Si | 4.32665000 | -0.43495000 | -0.26145000 |
| C | 4.55914400 | -2.14553700 | -1.08311600 |
| C | 4.59701000 | -2.35627600 | -2.48543200 |
| C | 4.70971300 | -3.28706900 | -0.24671500 |
| C | 4.77058600 | -3.64793700 | -3.00327700 |
| C | 4.87335500 | -4.55634300 | -0.80948000 |
| C | 4.90864600 | -4.77485600 | -2.19080900 |
| H | 4.79257900 | -3.75668900 | -4.08216400 |
| H | 4.97632800 | -5.40001300 | -0.13196000 |
| C | 5.76519300 | -0.16727800 | 0.99945700 |
| C | 7.08186000 | -0.23070800 | 0.45051900 |
| C | 5.66131900 | 0.14115400 | 2.37666600 |
| C | 8.19590200 | 0.00974200 | 1.26138700 |
| C | 6.80849200 | 0.37162300 | 3.15081600 |
| C | 8.09778600 | 0.31286500 | 2.62321900 |
| H | 9.17716000 | -0.04747100 | 0.79694700 |
| H | 6.66443400 | 0.60434700 | 4.20037300 |
| C | 4.46291100 | -1.23815900 | -3.50015300 |
| H | 3.48112500 | -0.75450200 | -3.45091100 |
| H | 5.21939600 | -0.45803500 | -3.36198600 |
| H | 4.58008900 | -1.62820500 | -4.51568700 |
| C | 4.72984700 | -3.21000100 | 1.26837600 |
| H | 5.69077000 | -2.83765700 | 1.64027300 |
| H | 3.95275100 | -2.55391600 | 1.67113300 |
| H | 4.56471400 | -4.20127700 | 1.70220400 |
| C | 5.09006000 | -6.19760500 | -2.74770500 |
| C | 3.92308800 | -7.09026300 | -2.26130200 |
| H | 4.04100300 | -8.11184400 | -2.64286600 |
| H | 3.87939000 | -7.14510600 | -1.16860600 |
| H | 2.96041800 | -6.70405100 | -2.61537600 |
| C | 5.10879900 | -6.22847100 | -4.28792500 |
| H | 4.17221200 | -5.85309700 | -4.71563000 |
| H | 5.93470900 | -5.63617600 | -4.69831300 |
| H | 5.23913800 | -7.26019700 | -4.63361500 |
| C | 6.42931900 | -6.78165700 | -2.23760600 |
| H | 7.27628100 | -6.17343000 | -2.57567800 |
| H | 6.46489700 | -6.82597200 | -1.14414800 |
| H | 6.56981300 | -7.80085000 | -2.61813700 |
| C | 4.33698800 | 0.22999500 | 3.09644300 |
| H | 3.67715200 | 0.98612000 | 2.66095700 |
| H | 3.79402900 | -0.72005300 | 3.07572700 |
| H | 4.48147500 | 0.49666800 | 4.14793500 |
| C | 7.37891800 | -0.57762700 | -0.99758200 |
| H | 7.22800300 | -1.64340300 | -1.19938000 |
| H | 6.75248500 | -0.02850900 | -1.70691800 |
| H | 8.41973400 | -0.33904600 | -1.23756700 |
| C | 9.36501600 | 0.56128500 | 3.45946600 |
| C | 10.25551200 | -0.70404000 | 3.42808000 |
| H | 11.16943600 | -0.54116000 | 4.01236800 |
| H | 9.72803300 | -1.56480700 | 3.85506600 |
| H | 10.55371400 | -0.96722400 | 2.40787700 |
| C | 9.04272000 | 0.88573400 | 4.93052600 |
| H | 8.43302800 | 1.79161900 | 5.02425900 |
| H | 8.51192300 | 0.06309500 | 5.42310100 |
| H | 9.97349900 | 1.05598900 | 5.48342300 |
| C | 10.14924500 | 1.75452300 | 2.86308500 |

| | | | |
|---|---|---|---|
| H | 10.44275000 | 1.57137800 | 1.82420300 |
| H | 9.54620700 | 2.66957300 | 2.88377900 |
| H | 11.06356300 | 1.93670900 | 3.44119400 |
| C | 3.40569900 | 3.56124200 | -0.80937300 |
| C | 3.42806200 | 4.58127400 | 0.14806700 |
| C | 3.35675200 | 3.94752800 | -2.15690400 |
| C | 3.38459400 | 5.92860600 | -0.21612400 |
| H | 3.48242700 | 4.32140200 | 1.20312400 |
| C | 3.31228400 | 5.29163400 | -2.51856300 |
| H | 3.35665100 | 3.19502400 | -2.93991700 |
| C | 3.32122600 | 6.31973200 | -1.55990900 |
| H | 3.40552200 | 6.67442500 | 0.57086700 |
| H | 3.27331200 | 5.53907200 | -3.57603600 |
| C | 3.26896800 | 7.79224500 | -2.00450100 |
| C | 4.49232900 | 8.10556500 | -2.89899500 |
| H | 4.51791400 | 7.47241000 | -3.79208900 |
| H | 4.46373500 | 9.15038500 | -3.23212800 |
| H | 5.42888300 | 7.94906900 | -2.35139700 |
| C | 1.96998300 | 8.03959300 | -2.80858900 |
| H | 1.91320000 | 7.40297200 | -3.69765800 |
| H | 1.08491600 | 7.83580300 | -2.19512600 |
| H | 1.92047800 | 9.08355000 | -3.14206300 |
| C | 3.28645400 | 8.76484600 | -0.81014600 |
| H | 2.42658100 | 8.61206800 | -0.14805400 |
| H | 4.20071900 | 8.66257400 | -0.21439700 |
| H | 3.24361600 | 9.79739700 | -1.17461300 |
| C | 4.32853600 | 1.19361800 | -1.25384000 |
| H | 5.32990700 | 1.62105800 | -1.35732100 |
| H | 3.89515100 | 1.11374700 | -2.25325900 |
| C | 3.45451700 | 2.10686100 | -0.36716800 |
| H | 3.88525900 | 2.11208700 | 0.64452800 |
| Lu | -0.94908600 | -1.82401600 | -0.00029200 |
| Lu | -3.90944300 | -0.19971500 | 1.22925600 |
| Lu | -1.47035700 | 1.57507000 | -0.63704500 |

**3A**-II

| | | | |
|---|---|---|---|
| C | -2.38151800 | 2.74544100 | 3.00686900 |
| C | -2.79083300 | 1.61710200 | 3.78879100 |
| C | -1.86441300 | 0.58304000 | 4.13303300 |
| C | -0.50074700 | 0.75433000 | 3.74092400 |
| C | -4.15771600 | 1.31366300 | 3.46258600 |
| C | -2.36387700 | -0.75537400 | 4.22410700 |
| C | -3.72367500 | -1.06365800 | 3.87889200 |
| C | -4.61946900 | -0.03886700 | 3.44829600 |
| C | -2.53062700 | -0.99588500 | -3.81024600 |
| C | -2.03551600 | 0.34080700 | -3.89683500 |
| C | -0.68405600 | 0.64860100 | -3.51389400 |
| C | 0.20831100 | -0.36521600 | -3.06563800 |
| C | -0.25783400 | -1.70664600 | -3.09020200 |
| C | -1.60987600 | -2.02638100 | -3.46242000 |
| C | -0.66573800 | 1.95757300 | -2.92880300 |
| C | 1.21098800 | -0.01073500 | -2.06939100 |
| C | 1.25768500 | 1.31217300 | -1.52934000 |
| C | 0.26071200 | 2.27890600 | -1.90128500 |
| C | 0.24843000 | 3.05661100 | 0.43017700 |
| C | 1.19543000 | 2.05813700 | 0.82776900 |
| C | -0.08724200 | 1.88277900 | 2.96537300 |
| C | -1.03346300 | 2.86517300 | 2.55290500 |
| C | -0.83461100 | 3.46748800 | 1.28432500 |
| C | 1.00086100 | 1.47324200 | 2.10127500 |
| C | -5.56964000 | -2.46946400 | -0.50234300 |
| C | -5.38392100 | -1.89399100 | -1.80012100 |
| C | -4.30961100 | -2.30587600 | -2.66473100 |
| C | -3.36898100 | -3.28626600 | -2.23136700 |
| C | -3.59782700 | -3.90123800 | -0.94084300 |
| C | -4.73058200 | -3.58755200 | -0.09232900 |
| C | -3.89978000 | -1.17296300 | -3.44540200 |
| C | -2.02275700 | -3.15703500 | -2.67554000 |
| C | -4.32823000 | -3.79683400 | 1.31529100 |
| C | -6.04842200 | -1.58985300 | 0.51997200 |

| | | | |
|---|---|---|---|
| C | -5.61493400 | -1.73441500 | 1.89464500 |
| C | -4.73619400 | -2.79915600 | 2.31990800 |
| C | 1.28641200 | 0.05972800 | 2.37942700 |
| C | 0.32507100 | -0.36389300 | 3.38124300 |
| C | -3.48890700 | 3.14698000 | 2.16869000 |
| C | -4.58512700 | 2.24391500 | 2.45570900 |
| C | 0.17831200 | -2.64863800 | -2.09825700 |
| C | -0.91852100 | -3.54253500 | -1.83349300 |
| C | -4.71724600 | -0.05009700 | -3.06889600 |
| C | -5.63185000 | -0.49241500 | -2.05162400 |
| C | -0.20438400 | -1.68426800 | 3.39749700 |
| C | -1.54316500 | -1.87540700 | 3.87071600 |
| C | -1.12775800 | -4.08190200 | -0.53726200 |
| C | -1.94174400 | 3.90101200 | 0.46216400 |
| C | -3.31654200 | 3.77424600 | 0.88428300 |
| C | -4.19551300 | 1.28035600 | -3.07228400 |
| C | 1.11454500 | -2.27164200 | -1.08744100 |
| C | -3.75709400 | -2.38824500 | 3.29642500 |
| C | -2.38937700 | -2.87665300 | 3.28353900 |
| C | -1.93166800 | -3.73798300 | 2.24208000 |
| C | -2.91319400 | -4.20589000 | 1.27757100 |
| C | -2.49133400 | -4.28615900 | -0.10669100 |
| C | 1.83161300 | -0.83743000 | 1.40771500 |
| C | 1.75219200 | -0.98108000 | -1.15578900 |
| C | 1.23518100 | -2.13880000 | 1.40369700 |
| C | 0.27808200 | -2.57640400 | 2.40465000 |
| C | -0.56266100 | -3.58783300 | 1.83278200 |
| C | -0.15669700 | -3.76908000 | 0.46243900 |
| C | 0.94878400 | -2.89475200 | 0.19266000 |
| C | -5.58639200 | -0.40225100 | 2.46923700 |
| C | -6.02054000 | 0.53825900 | 1.46994600 |
| C | -6.30248100 | -0.19218500 | 0.26744300 |
| C | -6.04762800 | 0.38005500 | -1.01242300 |
| C | -5.49521400 | 1.87196500 | 1.42730500 |
| C | -0.17990900 | 3.23159600 | -0.93070900 |
| C | -1.53366400 | 3.72837000 | -0.92073000 |
| C | -2.49919500 | 3.38300200 | -1.93289300 |
| C | -2.85790200 | 1.45652600 | -3.52708400 |
| C | -2.01633200 | 2.46790000 | -2.92890400 |
| C | -3.91412800 | 3.33866300 | -1.55984100 |
| C | -4.33699500 | 3.54577200 | -0.14796300 |
| C | -5.35245800 | 2.52816100 | 0.13889100 |
| C | -5.58037400 | 1.74891800 | -1.05374700 |
| C | -4.69379100 | 2.22229600 | -2.08597800 |
| N | -2.10422700 | -0.19822600 | 0.16400100 |
| C | 2.44640200 | -0.28385800 | 0.07928600 |
| C | 2.09303300 | 1.38098600 | -0.23255100 |
| Si | 4.41430700 | -0.45040100 | -0.32566100 |
| C | 4.62159500 | -2.14880200 | -1.17263200 |
| C | 4.65771800 | -2.33902500 | -2.58191300 |
| C | 4.74926600 | -3.30047500 | -0.35395500 |
| C | 4.80769200 | -3.62375400 | -3.11050200 |
| C | 4.88686500 | -4.57126000 | -0.93195100 |
| H | 4.83096700 | -3.72541600 | -4.19229700 |
| H | 4.96960100 | -5.42003900 | -0.26188400 |
| C | 5.86837800 | -0.19578400 | 0.91677400 |
| C | 7.16986100 | -0.24913100 | 0.34204600 |
| C | 5.79093100 | 0.09518700 | 2.30378100 |
| C | 8.30640500 | -0.01958300 | 1.13271600 |
| C | 6.95201500 | 0.31541500 | 3.05087100 |
| H | 9.27257200 | -0.07036400 | 0.64230600 |
| H | 6.83633900 | 0.53740600 | 4.10856600 |
| C | 3.40936200 | 3.54182400 | -0.83109800 |
| C | 3.48135200 | 4.56292600 | 0.12227600 |
| C | 3.27764800 | 3.92359600 | -2.17435800 |
| C | 3.41367000 | 5.90931100 | -0.24199800 |
| H | 3.59590400 | 4.30552000 | 1.17310100 |
| C | 3.20746800 | 5.26659700 | -2.53509800 |
| H | 3.23019300 | 3.16849600 | -2.95318000 |
| C | 3.27134500 | 6.29677500 | -1.58057500 |

| | | | |
|---|---|---|---|
| H | 3.47720900 | 6.65691900 | 0.54089500 |
| H | 3.10286500 | 5.51201800 | -3.58847300 |
| C | 3.18817000 | 7.76792200 | -2.02480600 |
| C | 4.35340700 | 8.08184000 | -2.99346600 |
| H | 4.32505100 | 7.44703100 | -3.88533300 |
| H | 4.30185900 | 9.12591400 | -3.32598400 |
| H | 5.32224000 | 7.92845200 | -2.50431600 |
| C | 1.84166500 | 8.00911900 | -2.74864100 |
| H | 1.73472000 | 7.37093600 | -3.63193200 |
| H | 0.99624200 | 7.80224900 | -2.08264500 |
| H | 1.76782300 | 9.05242500 | -3.07938300 |
| C | 3.27669900 | 8.74427900 | -0.83641500 |
| H | 2.45873600 | 8.59419200 | -0.12248800 |
| H | 4.22530000 | 8.64472500 | -0.29644000 |
| H | 3.21124600 | 9.77557900 | -1.20115500 |
| C | 4.36938600 | 1.19280400 | -1.28957800 |
| H | 5.36440300 | 1.63519700 | -1.39215000 |
| H | 3.93053200 | 1.11846500 | -2.28657200 |
| C | 3.49147000 | 2.08847000 | -0.39108800 |
| H | 3.92586700 | 2.09601500 | 0.61881800 |
| Lu | -0.05414600 | -0.13095800 | 0.16189300 |
| Lu | -3.05340600 | 1.59718300 | -0.23826600 |
| Lu | -3.23071500 | -1.85103200 | 0.67668000 |
| C | 7.43916400 | -0.56950500 | -1.11746100 |
| H | 7.28546200 | -1.63144300 | -1.33663900 |
| H | 6.79971300 | -0.00715000 | -1.80457700 |
| H | 8.47469900 | -0.32488300 | -1.37321900 |
| C | 4.48086900 | 0.17363100 | 3.04990800 |
| H | 3.80328000 | 0.91861000 | 2.62306200 |
| H | 3.95040400 | -0.78406900 | 3.04678900 |
| H | 4.64369300 | 0.44941200 | 4.09629900 |
| C | 4.53980200 | -1.20480200 | -3.58006200 |
| H | 3.55727300 | -0.72233200 | -3.53510500 |
| H | 5.29514500 | -0.42755600 | -3.42168000 |
| H | 4.66942400 | -1.57956800 | -4.59983400 |
| C | 4.91907400 | -4.76897100 | -2.31206600 |
| C | 8.23498000 | 0.26382000 | 2.49571800 |
| C | 4.77222200 | -3.24229200 | 1.16188900 |
| H | 3.99577000 | -2.59128800 | 1.57450100 |
| H | 4.60805300 | -4.23843800 | 1.58456500 |
| H | 5.73437700 | -2.87517700 | 1.53616500 |
| C | 5.06583200 | -6.15543500 | -2.96242800 |
| C | 9.47405700 | 0.51844000 | 3.37113700 |
| C | 10.78845800 | 0.40450000 | 2.57533700 |
| H | 11.63923400 | 0.58923900 | 3.24092300 |
| H | 10.91813900 | -0.59417100 | 2.14281200 |
| H | 10.83979700 | 1.13884000 | 1.76339300 |
| C | 9.51520500 | -0.51817900 | 4.51933200 |
| H | 8.62413700 | -0.46179500 | 5.15324700 |
| H | 9.58088300 | -1.53855800 | 4.12447800 |
| H | 10.39035400 | -0.34350000 | 5.15696000 |
| C | 9.39409900 | 1.94319600 | 3.96979100 |
| H | 9.37661200 | 2.70049400 | 3.17760400 |
| H | 8.49594200 | 2.07832400 | 4.58138300 |
| H | 10.26527900 | 2.13777300 | 4.60728400 |
| C | 6.35187900 | -6.18493100 | -3.82278200 |
| H | 7.24023100 | -6.00537800 | -3.20621700 |
| H | 6.46480800 | -7.16364200 | -4.30495800 |
| H | 6.33231200 | -5.42484200 | -4.61073900 |
| C | 3.83873300 | -6.43104200 | -3.86446800 |
| H | 2.91143100 | -6.41479400 | -3.28068000 |
| H | 3.74271500 | -5.68846000 | -4.66349200 |
| H | 3.92754400 | -7.41775800 | -4.33533200 |
| C | 5.15769900 | -7.28631500 | -1.92012400 |
| H | 6.02905500 | -7.16987300 | -1.26552800 |
| H | 4.26093900 | -7.33536100 | -1.29212100 |
| H | 5.25607900 | -8.25066900 | -2.43128700 |

**3A**-III

| | | | |
|---|---|---|---|
| C | -2.42895400 | 2.24569800 | 3.35965700 |

| | | | |
|---|---|---|---|
| C | -2.99828400 | 1.05594900 | 3.94834700 |
| C | -2.19736900 | -0.11295200 | 4.20563500 |
| C | -0.80802200 | -0.01312600 | 3.85400500 |
| C | -4.38219900 | 0.94722700 | 3.50212300 |
| C | -2.85228400 | -1.41562300 | 4.11734800 |
| C | -4.27316300 | -1.54914200 | 3.69267000 |
| C | -5.03723700 | -0.33919500 | 3.32769200 |
| C | -2.44057300 | -0.28644300 | -4.00723400 |
| C | -1.81589100 | 0.99221200 | -3.80881300 |
| C | -0.47112300 | 1.09888600 | -3.31302500 |
| C | 0.25802900 | -0.06518900 | -2.98674000 |
| C | -0.33740400 | -1.33872000 | -3.27032600 |
| C | -1.65621100 | -1.49296300 | -3.84470900 |
| C | -0.34094200 | 2.31574800 | -2.53536100 |
| C | 1.17009100 | 0.02073100 | -1.88201000 |
| C | 1.33320700 | 1.24393200 | -1.13376300 |
| C | 0.60098300 | 2.42966600 | -1.43741900 |
| C | 0.45261800 | 2.77234500 | 1.00312000 |
| C | 1.20272600 | 1.56897200 | 1.27198200 |
| C | -0.24305000 | 1.18322900 | 3.27505000 |
| C | -1.04903500 | 2.30565200 | 2.98600300 |
| C | -0.68652500 | 3.08759100 | 1.83836300 |
| C | 0.83923500 | 0.81382400 | 2.40115900 |
| C | -5.80153700 | -1.93049500 | -1.14291900 |
| C | -5.48711800 | -1.19849300 | -2.32565900 |
| C | -4.40368400 | -1.60472100 | -3.19963100 |
| C | -3.63539100 | -2.81906800 | -3.01137600 |
| C | -3.96555100 | -3.53046400 | -1.79048100 |
| C | -5.03574800 | -3.11070800 | -0.89968400 |
| C | -3.83532100 | -0.37829400 | -3.73777000 |
| C | -2.21300400 | -2.78250300 | -3.41088200 |
| C | -4.70525100 | -3.50345400 | 0.44365800 |
| C | -6.30681400 | -1.20954300 | -0.01626400 |
| C | -5.96936100 | -1.60616700 | 1.32064100 |
| C | -5.14411800 | -2.73426100 | 1.57654300 |
| C | 0.95832600 | -0.63680600 | 2.45084900 |
| C | -0.06133900 | -1.12894800 | 3.33783300 |
| C | -3.42768500 | 2.87554600 | 2.54936600 |
| C | -4.61877900 | 2.07171300 | 2.61305700 |
| C | -0.05954600 | -2.46807100 | -2.44318600 |
| C | -1.19250200 | -3.37227400 | -2.50402900 |
| C | -4.57340900 | 0.74208600 | -3.22476700 |
| C | -5.58854800 | 0.23470000 | -2.35057100 |
| C | -0.70069700 | -2.37126000 | 3.10798100 |
| C | -2.09632200 | -2.47582900 | 3.47599500 |
| C | -1.55192700 | -4.03624000 | -1.26234200 |
| C | -1.67102600 | 3.77446200 | 1.04561200 |
| C | -3.07900300 | 3.60922400 | 1.36474500 |
| C | -3.92838900 | 1.99614900 | -2.95670900 |
| C | 0.84154100 | -2.33744200 | -1.31911000 |
| C | -4.30499700 | -2.68113000 | 2.75853500 |
| C | -2.98446600 | -3.25601300 | 2.66533600 |
| C | -2.51607200 | -3.98459400 | 1.50795200 |
| C | -3.41960900 | -4.13942200 | 0.41632500 |
| C | -2.94752300 | -4.15005700 | -0.94958000 |
| C | 1.40533300 | -1.37006900 | 1.34419400 |
| C | 1.52169400 | -1.10805800 | -1.11791700 |
| C | 0.80345800 | -2.63705700 | 1.13672400 |
| C | -0.23825700 | -3.13222300 | 1.99966700 |
| C | -1.12930800 | -3.93540300 | 1.21582100 |
| C | -0.64337800 | -3.94245000 | -0.14838900 |
| C | 0.55546500 | -3.15521700 | -0.18709900 |
| C | -5.86809100 | -0.41397500 | 2.15123300 |
| C | -6.13231000 | 0.71686000 | 1.28121700 |
| C | -6.39490700 | 0.22655300 | -0.03888100 |
| C | -6.00038000 | 0.97156000 | -1.19370000 |
| C | -5.48315000 | 1.97250500 | 1.47980700 |
| C | 0.21796300 | 3.33330000 | -0.32917200 |
| C | -1.14578200 | 3.93358300 | -0.30230600 |
| C | -2.10049700 | 3.71290900 | -1.38615000 |

| | | | |
|---|---|---|---|
| C | -2.54898800 | 2.11418600 | -3.29497800 |
| C | -1.64528200 | 2.94057200 | -2.51861000 |
| C | -3.50065800 | 3.58703900 | -1.07601800 |
| C | -3.99235600 | 3.57932600 | 0.28292100 |
| C | -5.18409800 | 2.76662300 | 0.33349200 |
| C | -5.42394100 | 2.26116800 | -0.98665000 |
| C | -4.39729600 | 2.77004200 | -1.85872100 |
| N | -2.32734400 | -0.18296200 | 0.06637300 |
| C | 2.16469200 | -0.69305100 | 0.21568400 |
| C | 2.07996100 | 0.96197000 | 0.17817200 |
| C | 4.37955700 | 0.64816900 | -0.79041600 |
| H | 5.42657700 | 0.96362300 | -0.78292500 |
| H | 4.00013400 | 0.77363500 | -1.80661100 |
| Si | 4.13520100 | -1.10090600 | -0.07188100 |
| C | 3.55941500 | 1.51280200 | 0.19270600 |
| H | 3.92696600 | 1.31347100 | 1.21001400 |
| C | 4.23656000 | -2.68649300 | -1.13723200 |
| C | 4.20250700 | -3.94726000 | -0.48690500 |
| C | 4.34736100 | -2.68626200 | -2.55566400 |
| C | 4.25886400 | -5.13405100 | -1.23375800 |
| C | 4.40802800 | -3.89419000 | -3.25551500 |
| C | 4.36110500 | -5.14346500 | -2.62431500 |
| H | 4.21931900 | -6.07101500 | -0.68861500 |
| H | 4.49158700 | -3.84739500 | -4.33824000 |
| C | 5.52180700 | -1.19322700 | 1.27460500 |
| C | 5.37663800 | -1.08550300 | 2.68196000 |
| C | 6.84922700 | -1.32262400 | 0.77502800 |
| C | 6.49641400 | -1.10293200 | 3.51892800 |
| C | 7.94331000 | -1.33204100 | 1.65464400 |
| C | 7.80375500 | -1.22468200 | 3.03736100 |
| H | 6.32717100 | -1.01630000 | 4.58909900 |
| H | 8.93200500 | -1.42857900 | 1.21896300 |
| C | 3.69775100 | 3.01188800 | -0.02112300 |
| C | 3.78167400 | 3.87181900 | 1.08387400 |
| C | 3.76623500 | 3.59532000 | -1.29105000 |
| C | 3.90946200 | 5.24884900 | 0.92416000 |
| H | 3.74983000 | 3.45485700 | 2.08822100 |
| C | 3.89152800 | 4.97753800 | -1.44995800 |
| H | 3.72540200 | 2.97166700 | -2.17915000 |
| C | 3.96286000 | 5.84140000 | -0.34899600 |
| H | 3.97364500 | 5.86944200 | 1.81381600 |
| H | 3.93701800 | 5.37377400 | -2.45844200 |
| C | 4.10558900 | 7.36763300 | -0.48635700 |
| C | 5.43389500 | 7.81985000 | 0.16605600 |
| H | 6.29225800 | 7.34736800 | -0.32527300 |
| H | 5.55002300 | 8.90762900 | 0.08257300 |
| H | 5.47334000 | 7.56066300 | 1.22933700 |
| C | 2.92177100 | 8.06281500 | 0.22783900 |
| H | 1.96574300 | 7.76362800 | -0.21671200 |
| H | 2.88606600 | 7.81574100 | 1.29405800 |
| H | 3.01166100 | 9.15269300 | 0.14140000 |
| C | 4.11198400 | 7.82519900 | -1.95741300 |
| H | 4.95370200 | 7.39866800 | -2.51509200 |
| H | 3.18486500 | 7.55039100 | -2.47328200 |
| H | 4.20559100 | 8.91615900 | -2.00413300 |
| C | 4.40999700 | -1.41917200 | -3.38492000 |
| H | 3.48911600 | -0.83198500 | -3.30288000 |
| H | 5.23948400 | -0.76833200 | -3.08786300 |
| C | 4.12933100 | -4.10475500 | 1.02039500 |
| H | 5.09067700 | -3.88207500 | 1.49621600 |
| H | 3.37894700 | -3.45348600 | 1.47767000 |
| C | 4.03180500 | -0.95824700 | 3.35468100 |
| H | 4.14454300 | -0.87706000 | 4.44029800 |
| H | 3.48339800 | -0.07276300 | 3.02068600 |
| C | 7.19231300 | -1.48183200 | -0.69563700 |
| H | 6.93474100 | -2.47912000 | -1.06726800 |
| H | 6.67701200 | -0.76065300 | -1.33684200 |
| H | 8.26603900 | -1.33733400 | -0.85023100 |
| H | 3.39214000 | -1.82393700 | 3.15855900 |
| H | 3.85755300 | -5.13220800 | 1.28220000 |

| | | | |
|---|---|---|---|
| H | 4.54961300 | -1.66136800 | -4.44293800 |
| C | 4.42051800 | -6.43608900 | -3.45655300 |
| C | 5.74839900 | -6.47569600 | -4.25028600 |
| H | 5.80022400 | -7.38563500 | -4.86075200 |
| H | 6.61029100 | -6.47154600 | -3.57308400 |
| H | 5.84726000 | -5.61699300 | -4.92255800 |
| C | 3.23119200 | -6.46424700 | -4.44654800 |
| H | 3.25246800 | -5.61317400 | -5.13521000 |
| H | 2.27534300 | -6.43732700 | -3.91094600 |
| H | 3.25833400 | -7.38095200 | -5.04846900 |
| C | 4.34616400 | -7.70197100 | -2.58176500 |
| H | 3.41005500 | -7.75138800 | -2.01405100 |
| H | 5.18002700 | -7.75734100 | -1.87269200 |
| H | 4.39441100 | -8.59292700 | -3.21804400 |
| C | 8.99458500 | -1.23269800 | 4.01154000 |
| C | 10.34658200 | -1.37057800 | 3.28577300 |
| H | 11.16031900 | -1.37020300 | 4.01997700 |
| H | 10.52552100 | -0.53937200 | 2.59423900 |
| H | 10.41294400 | -2.30705600 | 2.72028800 |
| C | 8.84921400 | -2.42243200 | 4.99020200 |
| H | 8.84759900 | -3.37628700 | 4.45022800 |
| H | 7.92072500 | -2.36385300 | 5.56763200 |
| H | 9.68488300 | -2.43502300 | 5.70065900 |
| C | 9.01083200 | 0.09081800 | 4.81317700 |
| H | 8.09021500 | 0.22990000 | 5.38947100 |
| H | 9.12036400 | 0.95254000 | 4.14477300 |
| H | 9.85086700 | 0.09826700 | 5.51862600 |
| Lu | -1.32913200 | 1.58751900 | -0.26748900 |
| Lu | -3.15898300 | -0.60948700 | 1.88224800 |
| Lu | -2.36863100 | -1.55332700 | -1.45063900 |

**3B**-I

| | | | |
|---|---|---|---|
| C | 2.64446300 | 1.39186600 | -3.50710000 |
| C | 3.09013600 | 0.05000000 | -3.72807200 |
| C | 2.17051400 | -1.04302600 | -3.69971700 |
| C | 0.78802000 | -0.74962300 | -3.51686500 |
| C | 4.43247300 | -0.08395900 | -3.19163600 |
| C | 2.66410000 | -2.29071200 | -3.20619600 |
| C | 4.01247300 | -2.41421200 | -2.67972100 |
| C | 4.96995600 | -1.33045400 | -2.69676700 |
| C | 2.20031700 | 0.71760300 | 4.20206000 |
| C | 1.72261500 | 1.96943800 | 3.69708500 |
| C | 0.40786700 | 2.08060200 | 3.13458400 |
| C | -0.43946300 | 0.94288600 | 3.05545700 |
| C | -0.02452500 | -0.27304000 | 3.66295800 |
| C | 1.29122300 | -0.38862100 | 4.22320300 |
| C | 0.42408100 | 3.08899600 | 2.09622500 |
| C | -1.27702200 | 0.83817100 | 1.90031000 |
| C | -1.32449900 | 1.86391000 | 0.90615700 |
| C | -0.46010800 | 3.00770300 | 0.95515700 |
| C | -0.20532700 | 2.71754900 | -1.47186200 |
| C | -1.09281800 | 1.57987500 | -1.49945500 |
| C | 0.34562600 | 0.59439300 | -3.27561000 |
| C | 1.26762300 | 1.66648500 | -3.22580100 |
| C | 0.96988900 | 2.72415600 | -2.30608100 |
| C | -0.79514800 | 0.55640000 | -2.39677500 |
| C | 5.43788500 | -1.88393600 | 2.01849200 |
| C | 5.18613400 | -0.85575800 | 2.96755800 |
| C | 4.04186500 | -0.91456600 | 3.83728800 |
| C | 3.12105100 | -2.00383400 | 3.76974000 |
| C | 3.42130000 | -3.07568700 | 2.88138100 |
| C | 4.57060900 | -3.01755700 | 2.02113600 |
| C | 3.58576100 | 0.43106700 | 4.05417200 |
| C | 1.73701700 | -1.73316000 | 4.01762600 |
| C | 4.25330300 | -3.69319600 | 0.79271900 |
| C | 6.04898400 | -1.50983800 | 0.75743600 |
| C | 5.83366200 | -2.23100400 | -0.48155400 |
| C | 4.81296200 | -3.27441100 | -0.46128200 |
| C | -1.05420700 | -0.85459000 | -2.07933800 |
| C | -0.06336200 | -1.63611900 | -2.77526900 |

| | | | |
|---|---|---|---|
| C | 3.68251900 | 2.11274600 | -2.82716000 |
| C | 4.78167600 | 1.20180700 | -2.62073600 |
| C | -0.43454600 | -1.56073200 | 3.14437900 |
| C | 0.67632700 | -2.47587100 | 3.35916400 |
| C | 4.45242400 | 1.32642300 | 3.33737400 |
| C | 5.43853600 | 0.53102400 | 2.65755100 |
| C | 0.44339000 | -2.83269100 | -2.21936600 |
| C | 1.80884500 | -3.17847500 | -2.47993700 |
| C | 0.96820600 | -3.54632000 | 2.43061800 |
| C | 1.99134100 | 3.50530600 | -1.66374800 |
| C | 3.37989300 | 3.13080200 | -1.86852300 |
| C | 3.95071800 | 2.53062300 | 2.75636000 |
| C | -1.32711200 | -1.66274700 | 2.00638700 |
| C | 3.95040200 | -3.37727100 | -1.59765800 |
| C | 2.59053700 | -3.85545300 | -1.48677100 |
| C | 2.03453600 | -4.22250100 | -0.22172000 |
| C | 2.89544600 | -4.17593000 | 0.90221000 |
| C | 2.37052900 | -3.79297800 | 2.19461200 |
| C | -1.60862000 | -1.24635000 | -0.85989300 |
| C | -1.74577100 | -0.42499300 | 1.41351400 |
| C | -1.16545300 | -2.50188000 | -0.30385700 |
| C | -0.12405300 | -3.24379400 | -0.96979700 |
| C | 0.62272200 | -3.97731700 | 0.01292500 |
| C | 0.02736900 | -3.73683400 | 1.32244900 |
| C | -1.13072100 | -2.82733500 | 1.12111800 |
| C | 5.95739100 | -1.26847300 | -1.60419900 |
| C | 6.21735800 | 0.04718900 | -0.98554500 |
| C | 6.28830500 | -0.12339300 | 0.44835700 |
| C | 5.94683000 | 0.92303100 | 1.38971600 |
| C | 5.63013700 | 1.28693900 | -1.47735100 |
| C | 0.04701300 | 3.56381600 | -0.30964300 |
| C | 1.45059500 | 4.04215600 | -0.41790700 |
| C | 2.35139100 | 4.02014100 | 0.74074900 |
| C | 2.57976300 | 2.87121100 | 2.98803200 |
| C | 1.78753000 | 3.58524400 | 2.00187000 |
| C | 3.72976000 | 3.64824500 | 0.53014400 |
| C | 4.25557100 | 3.26117100 | -0.76208400 |
| C | 5.35946900 | 2.35263100 | -0.55875900 |
| C | 5.52026600 | 2.17749000 | 0.85838800 |
| C | 4.52703400 | 2.96965400 | 1.52948500 |
| N | 2.16497800 | -0.16439700 | 0.23354400 |
| C | -2.35600300 | -0.25010900 | 0.02295200 |
| C | -2.05745200 | 1.35175700 | -0.33607300 |
| Si | -4.31398900 | -0.49816200 | -0.42712500 |
| C | -3.46072000 | 2.03450700 | -0.58090300 |
| H | -3.94461800 | 2.05143200 | 0.40631900 |
| C | -4.26933300 | 1.08536600 | -1.49043200 |
| H | -3.76451000 | 0.96527800 | -2.45205500 |
| H | -5.26329500 | 1.50001200 | -1.68194400 |
| C | -3.41126700 | 3.48164600 | -1.04505400 |
| C | -3.51148300 | 4.51910300 | -0.10697600 |
| C | -3.28340200 | 3.84444200 | -2.39031900 |
| C | -3.46701100 | 5.85533300 | -0.49602100 |
| H | -3.62806300 | 4.27638600 | 0.94703200 |
| C | -3.24021000 | 5.18526800 | -2.77906500 |
| H | -3.21694300 | 3.07714600 | -3.15631700 |
| C | -3.32678500 | 6.22586200 | -1.84406800 |
| H | -3.54759400 | 6.62080000 | 0.27099200 |
| H | -3.13898500 | 5.40821200 | -3.83554100 |
| C | -3.27935000 | 7.71361300 | -2.23526600 |
| C | -4.59076000 | 8.40626200 | -1.79371800 |
| H | -4.56886900 | 9.46986600 | -2.06163500 |
| H | -5.45923000 | 7.95031200 | -2.28289200 |
| H | -4.74310800 | 8.33879200 | -0.71136800 |
| C | -3.11836000 | 7.91524200 | -3.75409900 |
| H | -2.18933700 | 7.47026500 | -4.12825800 |
| H | -3.95550700 | 7.48334500 | -4.31454300 |
| H | -3.08678100 | 8.98660200 | -3.98266500 |
| C | -2.08025200 | 8.39118600 | -1.52943600 |
| H | -2.15496400 | 8.31824000 | -0.43937700 |

| | | | |
|---|---|---|---|
| H | -1.13444700 | 7.92664700 | -1.83082100 |
| H | -2.03578800 | 9.45573200 | -1.79032700 |
| C | -4.50720300 | -2.23573000 | -1.20022400 |
| C | -4.66968100 | -3.34554900 | -0.33171700 |
| C | -4.51366600 | -2.49434900 | -2.59911000 |
| C | -4.81887500 | -4.64025800 | -0.85006700 |
| C | -4.67417900 | -3.80076600 | -3.06909300 |
| C | -4.82738700 | -4.90349800 | -2.21973900 |
| H | -4.93129000 | -5.45421500 | -0.14193500 |
| H | -4.67505000 | -3.95378300 | -4.14511900 |
| C | -5.80009800 | -0.19070400 | 0.76685300 |
| C | -5.75377100 | 0.16745000 | 2.14059700 |
| C | -7.09038100 | -0.28662300 | 0.17194000 |
| C | -6.93263100 | 0.40742200 | 2.85340400 |
| C | -8.24506500 | -0.03110700 | 0.92784000 |
| C | -8.20365500 | 0.31591500 | 2.27712000 |
| H | -6.84171200 | 0.67907400 | 3.90176800 |
| H | -9.20079900 | -0.11584900 | 0.42194100 |
| C | -4.72066900 | -3.21472900 | 1.17885600 |
| H | -3.97418600 | -2.51882800 | 1.57192100 |
| H | -5.70113000 | -2.86246800 | 1.51919600 |
| H | -4.53028000 | -4.18286600 | 1.65269900 |
| C | -4.35354700 | -1.41199200 | -3.64762000 |
| H | -5.10025900 | -0.61766700 | -3.54461000 |
| H | -3.36466200 | -0.94249000 | -3.59942200 |
| H | -4.46320400 | -1.83399500 | -4.65125700 |
| C | -7.32980500 | -0.68631000 | -1.27335300 |
| H | -8.36080300 | -0.45953800 | -1.56216700 |
| H | -6.67721800 | -0.16163500 | -1.97715300 |
| H | -7.16990000 | -1.75833100 | -1.43007300 |
| C | -4.46323400 | 0.31235600 | 2.91316700 |
| H | -3.87020400 | -0.60709700 | 2.90606500 |
| H | -3.82956200 | 1.10909100 | 2.51016600 |
| H | -4.66444700 | 0.56129800 | 3.95960300 |
| C | -4.99365700 | -6.31633600 | -2.80592100 |
| C | -3.75579300 | -6.66393000 | -3.66744600 |
| H | -3.62437300 | -5.95993800 | -4.49563100 |
| H | -2.84085300 | -6.64532800 | -3.06438300 |
| H | -3.86043000 | -7.66804600 | -4.09649200 |
| C | -6.26190400 | -6.35687600 | -3.69203300 |
| H | -6.39232800 | -7.35636300 | -4.12466700 |
| H | -7.15743600 | -6.12070000 | -3.10577300 |
| H | -6.20435200 | -5.64043800 | -4.51811200 |
| C | -5.13537500 | -7.39278900 | -1.71287400 |
| H | -4.25284600 | -7.43390400 | -1.06442800 |
| H | -6.01576400 | -7.22173300 | -1.08290100 |
| H | -5.24925300 | -8.37813300 | -2.17879900 |
| C | -9.46176700 | 0.59278200 | 3.11808900 |
| C | -10.75934700 | 0.43709600 | 2.30211700 |
| H | -10.87310300 | -0.57861100 | 1.90630800 |
| H | -11.62446300 | 0.64018100 | 2.94351300 |
| H | -10.80035200 | 1.13866700 | 1.46104600 |
| C | -9.40649400 | 2.03925900 | 3.66515800 |
| H | -8.52315400 | 2.20521600 | 4.29060300 |
| H | -9.37822800 | 2.76723700 | 2.84626900 |
| H | -10.29281500 | 2.24892800 | 4.27648800 |
| C | -9.51601600 | -0.40084700 | 4.30318300 |
| H | -9.56545900 | -1.43570000 | 3.94542900 |
| H | -8.63719900 | -0.31256600 | 4.95046100 |
| H | -10.40436100 | -0.21067800 | 4.91780000 |
| Lu | 1.50602800 | 1.77998000 | 0.19301200 |
| Lu | 0.84220500 | -1.55652400 | 0.96473200 |
| Lu | 3.93907300 | -0.75297800 | -0.58942500 |

**3B**-II

| | | | |
|---|---|---|---|
| C | 2.53014700 | 1.32443400 | -3.58914900 |
| C | 2.97212500 | -0.01937300 | -3.81456100 |
| C | 2.06006800 | -1.11840500 | -3.74203500 |
| C | 0.67899900 | -0.82456400 | -3.51864000 |
| C | 4.32125500 | -0.13665600 | -3.33234900 |

| | | | |
|---|---|---|---|
| C | 2.56011300 | -2.36599800 | -3.24806100 |
| C | 3.90096300 | -2.48054500 | -2.74571700 |
| C | 4.77812300 | -1.35349200 | -2.73943500 |
| C | 2.34311900 | 0.74480400 | 4.16178300 |
| C | 1.84877900 | 1.98953800 | 3.66507100 |
| C | 0.51815900 | 2.08879700 | 3.12943700 |
| C | -0.35600600 | 0.96498100 | 3.10042700 |
| C | 0.10373200 | -0.23725800 | 3.70000600 |
| C | 1.43612700 | -0.35169600 | 4.23100200 |
| C | 0.53193100 | 3.03827200 | 2.05588000 |
| C | -1.30980900 | 0.85854400 | 2.00400400 |
| C | -1.32960700 | 1.84416900 | 0.96755800 |
| C | -0.34642600 | 2.89265200 | 0.94946400 |
| C | -0.21340100 | 2.62403400 | -1.48794600 |
| C | -1.14480700 | 1.53554100 | -1.48046800 |
| C | 0.23398400 | 0.51711900 | -3.29806600 |
| C | 1.16372700 | 1.59743800 | -3.28484600 |
| C | 0.90738800 | 2.66487800 | -2.38723000 |
| C | -0.89569700 | 0.48406300 | -2.39264700 |
| C | 5.52982100 | -1.91026500 | 1.89970300 |
| C | 5.28862200 | -0.85566300 | 2.83684200 |
| C | 4.17123000 | -0.89107500 | 3.74322900 |
| C | 3.24756800 | -1.97673000 | 3.71051200 |
| C | 3.53277300 | -3.06275100 | 2.79870600 |
| C | 4.70262900 | -3.10703700 | 1.94695000 |
| C | 3.72715400 | 0.45611500 | 3.96440800 |
| C | 1.88244600 | -1.69879900 | 4.00180800 |
| C | 4.37477300 | -3.89612200 | 0.74229600 |
| C | 6.06224600 | -1.52749500 | 0.62858200 |
| C | 5.69433200 | -2.23751600 | -0.58031500 |
| C | 4.83855800 | -3.40171600 | -0.56689200 |
| C | -1.18093300 | -0.92398300 | -2.07999100 |
| C | -0.17014700 | -1.70886200 | -2.77051500 |
| C | 3.59755300 | 2.05454300 | -2.94481500 |
| C | 4.70403500 | 1.13463100 | -2.78510100 |
| C | -0.28801000 | -1.51226700 | 3.16949900 |
| C | 0.81831700 | -2.41691000 | 3.34703500 |
| C | 4.56727100 | 1.33682900 | 3.19585300 |
| C | 5.52932300 | 0.52765600 | 2.49666500 |
| C | 0.35902900 | -2.90489600 | -2.21038900 |
| C | 1.71985800 | -3.25115500 | -2.49871700 |
| C | 1.08721900 | -3.44087900 | 2.40185700 |
| C | 1.97308900 | 3.42012900 | -1.77109300 |
| C | 3.36336200 | 3.14507400 | -2.03661300 |
| C | 4.05134500 | 2.54140200 | 2.62500900 |
| C | -1.17399500 | -1.60208200 | 2.05152400 |
| C | 3.90546300 | -3.44744800 | -1.66805200 |
| C | 2.53504600 | -3.90683900 | -1.51302400 |
| C | 2.02510100 | -4.26928700 | -0.22983600 |
| C | 2.95782300 | -4.28157500 | 0.88439600 |
| C | 2.46859900 | -3.78165900 | 2.15403800 |
| C | -1.75454600 | -1.34258500 | -0.83987900 |
| C | -1.81801300 | -0.41098600 | 1.55091600 |
| C | -1.16991900 | -2.51460100 | -0.27257500 |
| C | -0.17072000 | -3.31312400 | -0.95841900 |
| C | 0.63845300 | -3.98256300 | 0.01838400 |
| C | 0.16604300 | -3.58631100 | 1.32090000 |
| C | -0.94625300 | -2.69656300 | 1.15319900 |
| C | 5.69514700 | -1.26199200 | -1.65492300 |
| C | 6.08299100 | 0.01477400 | -1.11685800 |
| C | 6.30746100 | -0.14729300 | 0.29123500 |
| C | 5.99362600 | 0.89861400 | 1.20814200 |
| C | 5.56067500 | 1.23585000 | -1.65441800 |
| C | 0.14630200 | 3.36074400 | -0.30800800 |
| C | 1.50100100 | 3.83641800 | -0.46284600 |
| C | 2.41744900 | 3.96836100 | 0.64338300 |
| C | 2.69207300 | 2.86453000 | 2.90331000 |
| C | 1.88358600 | 3.52705500 | 1.90344500 |
| C | 3.85174400 | 3.79598800 | 0.38792000 |
| C | 4.33146400 | 3.38562200 | -0.96054000 |

| | | | |
|---|---|---|---|
| C | 5.35267300 | 2.35873200 | -0.75665300 |
| C | 5.52867200 | 2.15435500 | 0.66006000 |
| C | 4.60371000 | 3.00751700 | 1.36447800 |
| N | 2.11046100 | -0.18067400 | 0.19580500 |
| C | -2.44449400 | -0.29583500 | 0.12358500 |
| C | -2.09648800 | 1.35334100 | -0.27527800 |
| Si | -4.41314500 | -0.47942000 | -0.31117200 |
| C | -3.49941000 | 2.03627600 | -0.49928800 |
| H | -3.95676200 | 2.06782000 | 0.49980200 |
| C | -4.34127300 | 1.09475600 | -1.38290200 |
| H | -3.85989000 | 0.95347400 | -2.35358600 |
| H | -5.32993200 | 1.52902600 | -1.55758000 |
| C | -3.42396700 | 3.47826500 | -0.97586700 |
| C | -3.44047100 | 4.52308300 | -0.04079000 |
| C | -3.34537100 | 3.82714500 | -2.32831900 |
| C | -3.36665100 | 5.85418300 | -0.44219800 |
| H | -3.51291100 | 4.29048700 | 1.01928000 |
| C | -3.27266500 | 5.16313700 | -2.72873900 |
| H | -3.34190600 | 3.05347900 | -3.09079400 |
| C | -3.27748600 | 6.21125300 | -1.79829400 |
| H | -3.38263800 | 6.62699100 | 0.32143600 |
| H | -3.21243700 | 5.37589100 | -3.79035300 |
| C | -3.19432200 | 7.69396300 | -2.20265900 |
| C | -4.46117400 | 8.43481200 | -1.71223500 |
| H | -4.41225500 | 9.49607100 | -1.98586700 |
| H | -5.36423800 | 8.00863300 | -2.16428600 |
| H | -4.57228800 | 8.37618600 | -0.62437700 |
| C | -3.08996800 | 7.87993000 | -3.72839500 |
| H | -2.19441500 | 7.39870800 | -4.13766100 |
| H | -3.96525700 | 7.47576200 | -4.24986800 |
| H | -3.02900000 | 8.94784900 | -3.96670900 |
| C | -1.94334700 | 8.33267300 | -1.55273100 |
| H | -1.97652200 | 8.27411300 | -0.45974700 |
| H | -1.02854200 | 7.82965200 | -1.88614700 |
| H | -1.86954800 | 9.39195800 | -1.82771600 |
| C | -4.62909700 | -2.21776900 | -1.06822900 |
| C | -4.73588100 | -3.32264100 | -0.18495700 |
| C | -4.69758300 | -2.48413200 | -2.46353800 |
| C | -4.90148100 | -4.62124900 | -0.68679700 |
| C | -4.87256100 | -3.79493200 | -2.91622500 |
| C | -4.97753800 | -4.89257000 | -2.05326500 |
| H | -4.97027400 | -5.43208900 | 0.03040800 |
| H | -4.92204600 | -3.95618700 | -3.98980900 |
| C | -5.87245000 | -0.14512400 | 0.90644400 |
| C | -5.80687800 | 0.21005600 | 2.28052300 |
| C | -7.17005100 | -0.21795300 | 0.32295700 |
| C | -6.97546800 | 0.47216000 | 3.00266000 |
| C | -8.31256600 | 0.06037100 | 1.08867000 |
| C | -8.25279700 | 0.40761600 | 2.43710100 |
| H | -6.86980700 | 0.74007000 | 4.05060300 |
| H | -9.27397400 | -0.00731500 | 0.59116100 |
| C | -4.69309400 | -3.17659700 | 1.32417800 |
| H | -3.88586100 | -2.51910900 | 1.66274600 |
| H | -5.63035700 | -2.76762200 | 1.71797000 |
| H | -4.52791600 | -4.14886300 | 1.79860000 |
| C | -4.58023100 | -1.40956800 | -3.52591000 |
| H | -5.31137200 | -0.60526800 | -3.39353100 |
| H | -3.58458200 | -0.95127700 | -3.53148800 |
| H | -4.74382300 | -1.83689200 | -4.51981300 |
| C | -7.43119300 | -0.61495100 | -1.11897800 |
| H | -8.46429600 | -0.38121200 | -1.39389000 |
| H | -6.78580700 | -0.09285100 | -1.83118200 |
| H | -7.28008600 | -1.68764900 | -1.27920000 |
| C | -4.50855600 | 0.33075500 | 3.04312500 |
| H | -3.91099500 | -0.58365000 | 2.99348700 |
| H | -3.88198900 | 1.14403500 | 2.66241000 |
| H | -4.69942700 | 0.54042300 | 4.10003900 |
| C | -5.16541700 | -6.30971800 | -2.62193800 |
| C | -3.97531900 | -6.65461600 | -3.54920300 |
| H | -3.89480900 | -5.95676200 | -4.38911100 |

| | | | |
|---|---|---|---|
| H | -3.02819100 | -6.62538300 | -2.99863500 |
| H | -4.09695000 | -7.66266200 | -3.96416500 |
| C | -6.48153900 | -6.36396200 | -3.43440700 |
| H | -6.62729100 | -7.36605500 | -3.85590000 |
| H | -7.34430600 | -6.13406300 | -2.79855000 |
| H | -6.47838600 | -5.64974000 | -4.26459800 |
| C | -5.23787300 | -7.38058700 | -1.51677000 |
| H | -4.32068600 | -7.41102100 | -0.91768800 |
| H | -6.08380000 | -7.21286200 | -0.84038500 |
| H | -5.36906600 | -8.36959300 | -1.97009300 |
| C | -9.49813100 | 0.71081800 | 3.28774700 |
| C | -10.80493900 | 0.58357200 | 2.48159100 |
| H | -10.94409900 | -0.42941600 | 2.08694100 |
| H | -11.66039500 | 0.80571300 | 3.12952700 |
| H | -10.83692900 | 1.28599600 | 1.64083900 |
| C | -9.40703700 | 2.15566200 | 3.83435900 |
| H | -8.51456200 | 2.30264900 | 4.45148000 |
| H | -9.37108700 | 2.88313000 | 3.01530700 |
| H | -10.28296100 | 2.38384900 | 4.45392400 |
| C | -9.56437500 | -0.28201900 | 4.47284000 |
| H | -9.63809600 | -1.31542600 | 4.11512000 |
| H | -8.67937600 | -0.21249900 | 5.11391600 |
| H | -10.44406400 | -0.07348800 | 5.09385700 |
| Lu | 0.06078000 | -0.14613900 | 0.07980100 |
| Lu | 3.23647800 | -1.89604700 | 0.42048800 |
| Lu | 3.02244400 | 1.65819900 | -0.06462500 |

**3B**-III

| | | | |
|---|---|---|---|
| C | 3.03732100 | 2.82092500 | 2.96797700 |
| C | 2.17701000 | 1.96295300 | 3.76086700 |
| C | 2.63588400 | 0.72897900 | 4.35339300 |
| C | 4.00059900 | 0.39548200 | 4.03584700 |
| C | 0.83772100 | 2.08563700 | 3.19587800 |
| C | 1.67002800 | -0.37432400 | 4.48758700 |
| C | 0.30765400 | -0.22858500 | 3.89252800 |
| C | -0.08350800 | 0.99225500 | 3.19987800 |
| C | 2.40836400 | -1.13177300 | -3.72007100 |
| C | 3.34550000 | -0.04123600 | -3.75908500 |
| C | 4.68053300 | -0.17572400 | -3.24871100 |
| C | 5.10432600 | -1.40206600 | -2.65455900 |
| C | 4.17716400 | -2.51283800 | -2.67065800 |
| C | 2.85971300 | -2.43168300 | -3.25123100 |
| C | 5.09781000 | 1.10409600 | -2.72460300 |
| C | 6.02180400 | -1.32843800 | -1.57991800 |
| C | 6.43190100 | -0.05188400 | -1.03712800 |
| C | 5.97613500 | 1.19503500 | -1.58885500 |
| C | 5.90710400 | 2.07306300 | 0.72271600 |
| C | 6.33158200 | 0.81020200 | 1.28762200 |
| C | 4.87095600 | 1.26242000 | 3.26062800 |
| C | 4.38436800 | 2.46629100 | 2.68106400 |
| C | 4.93350000 | 2.87358500 | 1.40908600 |
| C | 5.83260600 | 0.44811200 | 2.57831700 |
| C | -1.29034100 | -1.23459200 | -0.79583900 |
| C | -0.76973500 | -0.87235100 | -2.03905700 |
| C | 0.18600900 | -1.70512800 | -2.75282600 |
| C | 0.60958700 | -2.99656500 | -2.29131500 |
| C | 0.09828800 | -3.32875600 | -0.98075400 |
| C | -0.83509900 | -2.47043700 | -0.26681800 |
| C | 1.04193100 | -0.81000500 | -3.50792400 |
| C | 1.99105900 | -3.42161500 | -2.61203000 |
| C | -0.65190900 | -2.66139700 | 1.14307900 |
| C | -1.40846700 | -0.36061900 | 1.52400600 |
| C | -0.86329000 | -1.54657300 | 2.03812000 |
| C | 5.56933900 | -0.92769200 | 2.91198300 |
| C | 4.44577100 | -0.96343500 | 3.79988000 |
| C | 2.24175600 | 3.48312400 | 1.97186300 |
| C | 0.89080700 | 3.01991300 | 2.10356500 |
| C | 4.14798400 | -3.49383900 | -1.62435100 |
| C | 2.81552700 | -4.07544100 | -1.56166300 |
| C | 0.61298100 | 0.54027100 | -3.28861000 |

| | | | |
|---|---|---|---|
| C | -0.49687000 | 0.52053000 | -2.37286500 |
| C | 3.50685200 | -2.04020000 | 3.77972100 |
| C | 2.13013600 | -1.71693700 | 4.11388300 |
| C | 2.28299300 | -4.34016100 | -0.22378200 |
| C | 4.18410400 | 3.62136300 | 0.42564800 |
| C | 2.77914800 | 3.84656900 | 0.69075900 |
| C | 1.55656800 | 1.60702300 | -3.26010200 |
| C | 5.03161500 | -3.35117500 | -0.48504900 |
| C | -0.00972600 | -1.46546100 | 3.21214000 |
| C | 1.08769300 | -2.38072800 | 3.37305200 |
| C | 1.34716300 | -3.43834700 | 2.42198100 |
| C | 0.43970600 | -3.56284200 | 1.32235500 |
| C | 0.90260400 | -4.00396600 | 0.01917400 |
| C | 5.78161300 | -1.96976000 | 1.95537800 |
| C | 5.98384900 | -2.28843600 | -0.50508600 |
| C | 4.89532700 | -3.08801300 | 1.98661500 |
| C | 3.76453800 | -3.11679100 | 2.87875800 |
| C | 2.69370600 | -3.81313300 | 2.20979900 |
| C | 3.16293100 | -4.22789200 | 0.90398600 |
| C | 4.52800900 | -3.76845200 | 0.77731600 |
| C | -0.93759500 | 0.89391300 | 2.02433000 |
| C | -0.95784800 | 1.88293700 | 0.99966900 |
| C | -0.73720300 | 1.55537700 | -1.46362800 |
| C | 0.01668300 | 2.89998400 | 0.97923500 |
| C | 5.81337000 | 2.34010800 | -0.70150200 |
| C | 4.74139600 | 3.35305000 | -0.91091200 |
| C | 3.78125100 | 3.17214300 | -2.01973600 |
| C | 2.92655000 | 1.31519400 | -3.54477000 |
| C | 4.01158600 | 2.05713000 | -2.92585900 |
| C | 2.38558200 | 3.40188200 | -1.72764600 |
| C | 1.88098900 | 3.79623100 | -0.41760600 |
| C | 0.52429200 | 3.37038700 | -0.28599700 |
| C | 0.17545500 | 2.64408300 | -1.47481500 |
| C | 1.30649400 | 2.67165200 | -2.35981400 |
| N | 2.56655000 | -0.21996100 | 0.25043400 |
| Si | -3.95573000 | -0.44886500 | -0.32351800 |
| C | -1.97930500 | -0.21483800 | 0.11536000 |
| C | -1.66736000 | 1.36230600 | -0.25842900 |
| C | 6.63590700 | -0.23618800 | 0.37696900 |
| C | 6.36406900 | -1.61332800 | 0.70291100 |
| C | -3.89743800 | 1.12295900 | -1.39950900 |
| H | -4.88775500 | 1.54960000 | -1.58410400 |
| H | -3.40454800 | 0.98550100 | -2.36481500 |
| C | -3.06586400 | 2.06565500 | -0.50514200 |
| H | -3.54265300 | 2.10002500 | 0.48505500 |
| C | -4.16721300 | -2.19272100 | -1.08067000 |
| C | -4.29814900 | -3.29526700 | -0.19796100 |
| C | -4.20983600 | -2.46409200 | -2.47619800 |
| C | -4.45409800 | -4.59558700 | -0.69921900 |
| C | -4.37475900 | -3.77639600 | -2.92921000 |
| C | -4.49748900 | -4.87180900 | -2.06582700 |
| H | -4.54144800 | -5.40365100 | 0.01920900 |
| H | -4.40299600 | -3.94016200 | -4.00332600 |
| C | -2.99689100 | 3.50788000 | -0.98291400 |
| C | -3.03626700 | 4.55419600 | -0.05559000 |
| C | -2.90886700 | 3.85584800 | -2.33891900 |
| C | -2.97537100 | 5.89093000 | -0.45715500 |
| H | -3.11530500 | 4.32404400 | 1.00451200 |
| C | -2.85082600 | 5.18810500 | -2.73794100 |
| H | -2.88535000 | 3.07963400 | -3.09807600 |
| C | -2.87842700 | 6.24318400 | -1.80930000 |
| H | -3.00955100 | 6.65932600 | 0.30758400 |
| H | -2.78233600 | 5.40513600 | -3.80064600 |
| C | -5.44455800 | -0.12603300 | 0.86794800 |
| C | -5.40302200 | 0.23536500 | 2.24101300 |
| C | -6.73344300 | -0.21197800 | 0.26746400 |
| C | -6.58340300 | 0.48874000 | 2.94703500 |
| C | -7.88941200 | 0.05790100 | 1.01633600 |
| C | -7.85206600 | 0.40964600 | 2.36431900 |
| H | -6.49488800 | 0.76218900 | 3.99520200 |

| | | | |
|---|---|---|---|
| H | -8.84310700 | -0.01954600 | 0.50549100 |
| C | -2.80398000 | 7.70150300 | -2.29561300 |
| C | -3.99633300 | 7.99563800 | -3.23700200 |
| H | -3.95007800 | 9.02994400 | -3.59989600 |
| H | -3.99853000 | 7.33638200 | -4.11147000 |
| H | -4.95057200 | 7.86099600 | -2.71475200 |
| C | -2.85240700 | 8.70985800 | -1.13220100 |
| H | -3.78461800 | 8.62919400 | -0.56142900 |
| H | -2.01396300 | 8.57384400 | -0.43959300 |
| H | -2.79345900 | 9.73092400 | -1.52596200 |
| C | -1.47905500 | 7.91793000 | -3.06567400 |
| H | -0.61502000 | 7.72571400 | -2.41942400 |
| H | -1.39978700 | 7.25545200 | -3.93392900 |
| H | -1.41143400 | 8.95162700 | -3.42705000 |
| C | -4.07976500 | -1.39215900 | -3.53997000 |
| H | -4.81795200 | -0.59234500 | -3.42110900 |
| H | -3.08797100 | -0.92648600 | -3.52857100 |
| H | -4.22431400 | -1.82348500 | -4.53529800 |
| C | -4.29641400 | -3.14732700 | 1.31168700 |
| H | -5.25170300 | -2.75643300 | 1.67990400 |
| H | -4.12496700 | -4.11642600 | 1.79072000 |
| H | -3.51135000 | -2.47444300 | 1.67012500 |
| C | -4.66776600 | -6.29147400 | -2.63407500 |
| C | -5.95912300 | -6.35185800 | -3.48477100 |
| H | -6.09145000 | -7.35621000 | -3.90550600 |
| H | -6.84040800 | -6.11964800 | -2.87577900 |
| H | -5.93212800 | -5.64162400 | -4.31786000 |
| C | -4.77073800 | -7.35833000 | -1.52735700 |
| H | -3.87065900 | -7.38604100 | -0.90273000 |
| H | -5.63503100 | -7.18781300 | -0.87538200 |
| H | -4.88920000 | -8.34920500 | -1.98031600 |
| C | -3.45038700 | -6.63776800 | -3.52480000 |
| H | -3.34766000 | -5.94242900 | -4.36433700 |
| H | -2.51989100 | -6.60349300 | -2.94657800 |
| H | -3.55672400 | -7.64781900 | -3.93934800 |
| C | -6.97193800 | -0.61328200 | -1.17721900 |
| H | -6.32034800 | -0.08681100 | -1.88021200 |
| H | -6.80970900 | -1.68486200 | -1.33369600 |
| H | -8.00322300 | -0.38837400 | -1.46639200 |
| C | -4.11581800 | 0.37529400 | 3.01903400 |
| H | -3.50458700 | -0.53061700 | 2.97614900 |
| H | -3.49809800 | 1.19846600 | 2.64457000 |
| H | -4.32188100 | 0.58211400 | 4.07387200 |
| C | -9.11201400 | 0.70391300 | 3.19653400 |
| C | -10.40635600 | 0.56054000 | 2.37316700 |
| H | -11.27301600 | 0.77613800 | 3.00842800 |
| H | -10.43376000 | 1.25948700 | 1.52937500 |
| H | -10.52955600 | -0.45514600 | 1.98014200 |
| C | -9.18532200 | -0.28473700 | 4.38463900 |
| H | -9.24428800 | -1.32015700 | 4.02995000 |
| H | -8.30962800 | -0.20438900 | 5.03718400 |
| H | -10.07530200 | -0.08240100 | 4.99308500 |
| C | -9.04320600 | 2.15167100 | 3.73863300 |
| H | -8.16135800 | 2.30967700 | 4.36822300 |
| H | -9.00166900 | 2.87603300 | 2.91712000 |
| H | -9.93049400 | 2.37411000 | 4.34410900 |
| Lu | 2.19351200 | -1.88614100 | -0.87808900 |
| Lu | 1.93162500 | -0.12169800 | 2.18736100 |
| Lu | 3.63998600 | 1.35450600 | -0.50233800 |

**4A**-I

| | | | |
|---|---|---|---|
| C | -1.96579600 | 2.38089200 | 3.25056900 |
| C | -2.62760500 | 1.22531000 | 3.77482400 |
| C | -1.90629500 | 0.01219000 | 4.00563100 |
| C | -0.50048100 | 0.01556600 | 3.77555100 |
| C | -3.99203300 | 1.21021700 | 3.27959100 |
| C | -2.61555000 | -1.21106300 | 3.80569600 |
| C | -3.97359000 | -1.21407400 | 3.29479200 |
| C | -4.73543800 | -0.01160500 | 3.05996100 |
| C | -1.86416800 | -0.08579000 | -4.08689500 |

| | | | |
|---|---|---|---|
| C | -1.16511500 | 1.14648800 | -3.87322600 |
| C | 0.16597300 | 1.15784600 | -3.33806400 |
| C | 0.80793200 | -0.06200200 | -2.99444300 |
| C | 0.17129200 | -1.29143800 | -3.31064000 |
| C | -1.15854000 | -1.30754600 | -3.84738100 |
| C | 0.35924200 | 2.36929500 | -2.56523300 |
| C | 1.65071700 | -0.04323700 | -1.83748900 |
| C | 1.89422500 | 1.16064600 | -1.09899000 |
| C | 1.26003900 | 2.40548400 | -1.42868400 |
| C | 1.02485400 | 2.71741000 | 1.00808700 |
| C | 1.70056500 | 1.47604800 | 1.30234000 |
| C | 0.15937800 | 1.17285800 | 3.23839100 |
| C | -0.56872100 | 2.34600200 | 2.93297000 |
| C | -0.11875500 | 3.10023900 | 1.80218000 |
| C | 1.24965600 | 0.74153600 | 2.40267600 |
| C | -5.42608900 | -1.52545900 | -1.40095500 |
| C | -5.03625600 | -0.80067600 | -2.55911500 |
| C | -3.94657400 | -1.25114700 | -3.38183900 |
| C | -3.22176800 | -2.43773000 | -3.05553700 |
| C | -3.67135200 | -3.21120600 | -1.94727800 |
| C | -4.76840800 | -2.76486800 | -1.13451700 |
| C | -3.27352400 | -0.08791800 | -3.89256400 |
| C | -1.81997800 | -2.47390500 | -3.34368200 |
| C | -4.53363400 | -3.19145900 | 0.21681500 |
| C | -5.91649700 | -0.76787800 | -0.26868500 |
| C | -5.75979400 | -1.20758900 | 1.09801400 |
| C | -4.95685900 | -2.39976300 | 1.33192500 |
| C | 1.24845400 | -0.72554900 | 2.41586200 |
| C | 0.16324000 | -1.14829400 | 3.26388300 |
| C | -2.88605200 | 3.09253300 | 2.41343600 |
| C | -4.13969200 | 2.36828800 | 2.41992900 |
| C | 0.36173800 | -2.46912100 | -2.49597400 |
| C | -0.88432200 | -3.21846600 | -2.51805200 |
| C | -3.95297600 | 1.08104500 | -3.40548200 |
| C | -5.04344600 | 0.64223000 | -2.57551500 |
| C | -0.55299500 | -2.33835300 | 2.99713200 |
| C | -1.94664800 | -2.37842600 | 3.31941400 |
| C | -1.32044600 | -4.00196500 | -1.38040200 |
| C | -1.01704300 | 3.86574000 | 0.98516300 |
| C | -2.44185900 | 3.80170800 | 1.25486300 |
| C | -3.23784300 | 2.28089400 | -3.11058900 |
| C | 1.24444400 | -2.45657600 | -1.35495900 |
| C | -4.10832500 | -2.40158400 | 2.47603100 |
| C | -2.86118700 | -3.12476200 | 2.50184500 |
| C | -2.40996100 | -3.85307800 | 1.35516800 |
| C | -3.28129900 | -3.90829100 | 0.23763800 |
| C | -2.73490800 | -3.92632000 | -1.10429500 |
| C | 1.70813200 | -1.47700600 | 1.33531600 |
| C | 1.90423600 | -1.21654800 | -1.06779000 |
| C | 1.02710800 | -2.72452700 | 1.07339400 |
| C | -0.09990600 | -3.11599900 | 1.88155600 |
| C | -0.98297900 | -3.92642500 | 1.08718200 |
| C | -0.39412300 | -4.09353600 | -0.24097800 |
| C | 0.88962000 | -3.34537000 | -0.24062800 |
| C | -5.73075500 | -0.02468300 | 1.97536200 |
| C | -5.83742400 | 1.15766300 | 1.08075400 |
| C | -5.95095200 | 0.67274300 | -0.28426700 |
| C | -5.44737500 | 1.39482100 | -1.43847400 |
| C | -5.01238300 | 2.34806300 | 1.28300400 |
| C | 0.88523400 | 3.30942600 | -0.32318900 |
| C | -0.42932400 | 4.00382800 | -0.34060600 |
| C | -1.35522700 | 3.87127700 | -1.46572100 |
| C | -1.83613100 | 2.31799600 | -3.39890500 |
| C | -0.90519500 | 3.08734100 | -2.59335700 |
| C | -2.77288200 | 3.81976100 | -1.19742800 |
| C | -3.31647500 | 3.83233400 | 0.14131800 |
| C | -4.56273300 | 3.09737200 | 0.14237000 |
| C | -4.79090100 | 2.63972900 | -1.20199200 |
| C | -3.69781700 | 3.07981500 | -2.02436600 |
| N | -1.87351200 | -0.03154000 | -0.03721500 |

| | | | |
|---|---|---|---|
| C | 2.57733900 | -0.84672500 | 0.26375700 |
| C | 2.57562700 | 0.82305300 | 0.23574900 |
| Si | 4.52852800 | -1.35545500 | 0.02116400 |
| C | 4.55987700 | -2.94076900 | -1.07287900 |
| C | 4.71595100 | -2.92151700 | -2.49055100 |
| C | 4.44007800 | -4.20232900 | -0.42042100 |
| C | 4.75423100 | -4.12761600 | -3.20049000 |
| C | 4.48631900 | -5.38441100 | -1.17165300 |
| C | 4.64361600 | -5.35195600 | -2.55068200 |
| H | 4.86967700 | -4.11501900 | -4.27806500 |
| H | 4.39252500 | -6.34266500 | -0.67319800 |
| C | 5.88327500 | -1.52522100 | 1.40726400 |
| C | 7.22225500 | -1.71588200 | 0.94207300 |
| C | 5.67364000 | -1.41066400 | 2.80898100 |
| C | 8.27754900 | -1.77167100 | 1.86328700 |
| C | 6.75774100 | -1.47264000 | 3.69320700 |
| C | 8.05437600 | -1.65036000 | 3.22808800 |
| H | 9.29183700 | -1.91271600 | 1.50793400 |
| H | 6.58706200 | -1.38136200 | 4.75980600 |
| C | 4.84276600 | -1.61651200 | -3.27082700 |
| H | 3.94753100 | -1.01006300 | -3.08964100 |
| H | 5.67924800 | -1.03603900 | -2.86338200 |
| C | 4.28044100 | -4.31477700 | 1.09273300 |
| H | 5.25466300 | -4.15120400 | 1.57049400 |
| H | 3.64197300 | -3.49821900 | 1.44514300 |
| C | 4.27813000 | -1.22763700 | 3.37971800 |
| H | 3.84629900 | -0.30334900 | 2.97894200 |
| H | 3.63794900 | -2.03199600 | 3.00252600 |
| C | 7.56207600 | -1.90085000 | -0.53593400 |
| H | 7.24577900 | -2.90298200 | -0.85042100 |
| H | 6.96384800 | -1.21045300 | -1.13858900 |
| C | 4.31220300 | 2.78155200 | 0.12166300 |
| C | 4.40148300 | 3.61852100 | 1.23934200 |
| C | 4.46366800 | 3.37775700 | -1.13909700 |
| C | 4.61315100 | 4.99284100 | 1.11015500 |
| H | 4.30603800 | 3.19113700 | 2.23527800 |
| C | 4.67410200 | 4.74841400 | -1.26632600 |
| H | 4.42191300 | 2.77000900 | -2.03810800 |
| C | 4.75116000 | 5.59517100 | -0.14709900 |
| H | 4.67341100 | 5.59021000 | 2.01322800 |
| H | 4.78473500 | 5.16213200 | -2.26503800 |
| C | 4.98575200 | 7.10434600 | -0.33521400 |
| C | 6.32753900 | 7.32993000 | -1.07258400 |
| H | 6.33581000 | 6.85386500 | -2.05870500 |
| H | 6.50676000 | 8.40228300 | -1.21843100 |
| H | 7.16523300 | 6.92030200 | -0.49649300 |
| C | 3.83234000 | 7.70212200 | -1.17610400 |
| H | 3.76575700 | 7.23716100 | -2.16532400 |
| H | 2.86801400 | 7.56014200 | -0.67503200 |
| H | 3.98509800 | 8.77844700 | -1.32326000 |
| C | 5.04234500 | 7.85905200 | 1.00666500 |
| H | 4.10604500 | 7.76192700 | 1.56791000 |
| H | 5.86133100 | 7.50264100 | 1.64200700 |
| H | 5.20808200 | 8.92661300 | 0.82274700 |
| C | 4.88356900 | 0.39374900 | -0.65959900 |
| H | 5.94499900 | 0.65232200 | -0.61095800 |
| H | 4.54758800 | 0.55888300 | -1.68512900 |
| C | 4.08125400 | 1.28950200 | 0.30760200 |
| H | 4.40262800 | 1.05850800 | 1.33286700 |
| Lu | -0.92047900 | -1.81899600 | -0.36765400 |
| Lu | -3.69791600 | 0.12913700 | 0.86615200 |
| Lu | -0.78907700 | 1.67153500 | -0.43225100 |
| H | 8.88672400 | -1.69488900 | 3.92590500 |
| H | 4.67538300 | -6.27735000 | -3.12019400 |
| C | 4.13545100 | -1.18874400 | 4.90461800 |
| H | 4.50215700 | -2.10904900 | 5.37309000 |
| H | 4.67603200 | -0.34457300 | 5.34716000 |
| H | 3.07840300 | -1.07975200 | 5.16944000 |
| C | 9.03006800 | -1.70577600 | -0.93980800 |
| H | 9.68353800 | -2.47708500 | -0.51869600 |

| | | | |
|---|---|---|---|
| H | 9.12095400 | -1.76748700 | -2.02978600 |
| H | 9.41334400 | -0.72831000 | -0.62525500 |
| C | 5.03927700 | -1.72519200 | -4.78742600 |
| H | 5.12555500 | -0.72207700 | -5.21908600 |
| H | 5.95137800 | -2.27515400 | -5.04497500 |
| H | 4.19404900 | -2.22150900 | -5.27669800 |
| C | 3.68192700 | -5.62379100 | 1.62449200 |
| H | 4.34846100 | -6.47882200 | 1.46870600 |
| H | 3.51257200 | -5.54048400 | 2.70355200 |
| H | 2.72184600 | -5.85004100 | 1.14754300 |

**4A**-II

| | | | |
|---|---|---|---|
| C | -0.03630300 | 1.28920400 | -3.27497900 |
| C | 1.30784300 | 1.31549800 | -3.77694300 |
| C | 2.01333400 | 0.09750700 | -4.01608300 |
| C | 1.30867300 | -1.12926900 | -3.85824700 |
| C | 1.97249200 | 2.47993400 | -3.22895200 |
| C | 3.41766100 | 0.10684600 | -3.75845700 |
| C | 4.06847100 | 1.26993600 | -3.19560700 |
| C | 3.38927600 | 2.51814000 | -2.95275600 |
| C | 1.79860800 | -0.12856500 | 4.09811400 |
| C | 0.39445600 | -0.12849300 | 3.82744200 |
| C | -0.26009300 | -1.27775800 | 3.27548100 |
| C | 0.48048900 | -2.45447500 | 2.97302100 |
| C | 1.87353300 | -2.49959700 | 3.31503600 |
| C | 2.52022000 | -1.34556700 | 3.85893400 |
| C | -1.36353000 | -0.83224300 | 2.44224100 |
| C | 0.07655400 | -3.18927600 | 1.83253600 |
| C | -1.01385900 | -2.73873600 | 0.99785200 |
| C | -1.79294700 | -1.57904000 | 1.30344200 |
| C | -1.89972500 | -1.17935300 | -1.17281900 |
| C | -1.09297300 | -2.34209900 | -1.44250000 |
| C | -0.73667000 | -3.19549700 | -0.35187300 |
| C | -0.03802600 | -1.14073800 | -3.34571000 |
| C | -0.70936200 | 0.05976500 | -3.00774600 |
| C | -1.66033400 | 0.02452100 | -1.90464200 |
| C | -0.21951500 | -2.33206900 | -2.56475300 |
| C | 4.90308900 | 2.30502200 | 1.55537300 |
| C | 4.04372400 | 2.30390800 | 2.68758000 |
| C | 3.86850300 | 1.12056000 | 3.48569100 |
| C | 4.54880200 | -0.08793000 | 3.15717100 |
| C | 5.47824400 | -0.05995500 | 2.08041000 |
| C | 5.66407500 | 1.12611300 | 1.29251100 |
| C | 2.50558100 | 1.09865900 | 3.94830200 |
| C | 3.87921100 | -1.33282200 | 3.39942000 |
| C | 5.96231900 | 0.73096200 | -0.05735300 |
| C | 4.52091100 | 3.12041900 | 0.42289800 |
| C | 4.86428800 | 2.78300300 | -0.93842000 |
| C | 5.51444800 | 1.50411800 | -1.17609800 |
| C | 1.02687700 | -3.05117900 | -2.56397600 |
| C | 1.97164600 | -2.30003300 | -3.35245300 |
| C | -0.22764800 | 2.42433900 | -2.42411700 |
| C | 1.01215600 | 3.16137000 | -2.38160200 |
| C | 2.83442700 | -3.23067300 | 2.51750900 |
| C | 4.10221400 | -2.50582100 | 2.57851000 |
| C | 1.84648900 | 2.27532700 | 3.46087300 |
| C | 2.79299600 | 3.02260800 | 2.67378400 |
| C | 3.34724300 | -2.26290400 | -3.00341000 |
| C | 4.07983700 | -1.06451200 | -3.26552400 |
| C | 5.02499900 | -2.48834100 | 1.46093800 |
| C | -1.93401000 | 1.19217300 | -1.11382400 |
| C | -1.11203600 | 2.35582600 | -1.30386800 |
| C | 0.46337500 | 2.24300800 | 3.10883200 |
| C | 2.43226300 | -3.95423400 | 1.33539300 |
| C | 5.13726400 | 0.78676700 | -2.34630500 |
| C | 5.14901600 | -0.65144900 | -2.39933100 |
| C | 5.51710100 | -1.41854500 | -1.25109200 |
| C | 5.95390700 | -0.70896900 | -0.10229100 |
| C | 5.65888400 | -1.21516700 | 1.22305200 |
| C | 1.43479800 | -3.80066400 | -1.42746800 |

| | | | |
|---|---|---|---|
| C | 1.02623100 | -3.90127600 | 1.01872300 |
| C | 0.51809200 | -3.88114300 | -0.33687400 |
| C | 2.84391800 | -3.84695000 | -1.10983900 |
| C | 3.76438900 | -3.05791900 | -1.87669800 |
| C | 4.86486800 | -2.69124700 | -1.02585800 |
| C | 4.67693100 | -3.29916300 | 0.28807100 |
| C | 3.38359100 | -4.02631400 | 0.23228600 |
| C | 3.85568900 | 3.35787700 | -1.83845100 |
| C | 2.85627500 | 4.03171900 | -0.96861400 |
| C | 3.29252200 | 3.87186700 | 0.41131800 |
| C | 2.37813900 | 3.76450000 | 1.53307200 |
| C | 1.42485500 | 3.90197400 | -1.22466200 |
| C | -1.81176300 | 1.44720200 | 1.39798400 |
| C | -0.27219900 | 1.04146300 | 3.34334200 |
| C | -1.37810600 | 0.63793500 | 2.49176400 |
| C | -1.03355300 | 2.62082400 | 1.15245200 |
| C | -0.74010200 | 3.13178600 | -0.16860300 |
| C | 0.51101100 | 3.84361600 | -0.11446100 |
| C | 0.98062000 | 3.80556700 | 1.24850000 |
| C | 0.04164900 | 3.04993000 | 2.02004300 |
| N | 1.80280200 | -0.02849900 | 0.03534000 |
| C | -4.92193500 | 0.40363300 | -0.74005900 |
| H | -4.57195100 | 0.64115900 | -1.74617000 |
| H | -5.98853900 | 0.64122100 | -0.69116300 |
| C | -2.62782500 | -0.90676300 | 0.18145200 |
| C | -2.64401700 | 0.83305300 | 0.22767100 |
| C | -4.38355800 | 2.75279700 | 0.17970400 |
| C | -4.78717800 | 4.89448500 | 1.27644000 |
| C | -4.63044400 | 4.80284000 | -1.10677500 |
| C | -4.81599700 | 5.57556300 | 0.05295200 |
| H | -4.93137600 | 5.43244700 | 2.20665400 |
| H | -4.65011300 | 5.28101500 | -2.08235900 |
| C | -4.51816800 | -2.87981200 | -1.38372600 |
| C | -4.38100900 | -4.18009900 | -0.81965100 |
| C | -4.64926500 | -2.76211700 | -2.79987700 |
| C | -4.39624700 | -5.30819500 | -1.65141900 |
| C | -4.65525000 | -3.91644700 | -3.59128900 |
| H | -4.29255200 | -6.29740500 | -1.21909400 |
| H | -4.75144700 | -3.83145500 | -4.66747600 |
| C | -5.03974900 | 7.09421500 | -0.05802600 |
| C | -6.31120700 | 7.36887900 | -0.89628300 |
| H | -7.19545300 | 6.92497300 | -0.42462300 |
| H | -6.48073500 | 8.44877800 | -0.98849600 |
| H | -6.22979300 | 6.95728700 | -1.90783900 |
| C | -5.21879100 | 7.76162300 | 1.31891700 |
| H | -6.08979900 | 7.36629100 | 1.85404500 |
| H | -4.33613600 | 7.62803500 | 1.95467700 |
| H | -5.37235500 | 8.83893800 | 1.18951400 |
| C | -3.81836400 | 7.74277200 | -0.75291700 |
| H | -2.90182000 | 7.56743200 | -0.17820300 |
| H | -3.66245100 | 7.34275300 | -1.76031400 |
| H | -3.96218500 | 8.82655300 | -0.84274000 |
| C | -4.57662200 | 3.51513400 | 1.33638300 |
| H | -4.56469900 | 3.02493800 | 2.30757400 |
| C | -4.42242100 | 3.42718400 | -1.04963000 |
| H | -4.28984000 | 2.87814600 | -1.97694000 |
| C | -4.53536700 | -5.18182000 | -3.02632800 |
| H | -4.54375000 | -6.06522500 | -3.65972500 |
| C | -4.15504500 | 1.25276100 | 0.29223300 |
| H | -4.49284200 | 0.96618100 | 1.29787100 |
| Si | -4.56779000 | -1.38565900 | -0.17387200 |
| C | -5.95892200 | -1.69247500 | 1.14683800 |
| C | -7.27322000 | -1.88998300 | 0.61939400 |
| C | -5.80066200 | -1.68068600 | 2.56007900 |
| C | -8.35514000 | -2.06345800 | 1.49389100 |
| C | -6.91016400 | -1.85661700 | 3.39585900 |
| C | -8.18183500 | -2.04872300 | 2.87098500 |
| H | -9.35077100 | -2.21116000 | 1.09129900 |
| H | -6.78009900 | -1.84206500 | 4.47189000 |
| H | -9.03393100 | -2.18333600 | 3.53240400 |

| | | | |
|---|---|---|---|
| C | -7.55879600 | -1.95236000 | -0.87959900 |
| H | -6.93570000 | -1.21975600 | -1.40097900 |
| H | -7.23637200 | -2.92790100 | -1.26413000 |
| C | -9.01013200 | -1.71050500 | -1.31781800 |
| H | -9.40072500 | -0.76371000 | -0.92764800 |
| H | -9.06083700 | -1.67065500 | -2.41141000 |
| H | -9.68205300 | -2.51250800 | -0.99480100 |
| C | -4.43414200 | -1.49002600 | 3.19558600 |
| H | -3.77471800 | -2.29979200 | 2.86118900 |
| H | -3.98591700 | -0.57093500 | 2.80444500 |
| C | -4.36571000 | -1.42953800 | 4.72497000 |
| H | -4.71760300 | -2.35779200 | 5.18860500 |
| H | -3.32733000 | -1.27794500 | 5.03801100 |
| H | -4.95945900 | -0.60188100 | 5.12900200 |
| C | -4.23721000 | -4.39988200 | 0.68312400 |
| H | -3.71575800 | -3.54482400 | 1.12384800 |
| H | -5.23306100 | -4.40464900 | 1.14610600 |
| C | -3.48242600 | -5.66513200 | 1.11534300 |
| H | -2.50085300 | -5.72855600 | 0.63348900 |
| H | -3.32810700 | -5.65149900 | 2.19982000 |
| H | -4.03631400 | -6.58034100 | 0.88180200 |
| C | -4.78173900 | -1.40855000 | -3.49147800 |
| H | -5.64768700 | -0.87677600 | -3.07850200 |
| H | -3.90895100 | -0.79690500 | -3.23558800 |
| C | -4.92046200 | -1.41481000 | -5.01827500 |
| H | -5.00967200 | -0.38544200 | -5.38212400 |
| H | -4.04831300 | -1.86213200 | -5.50739200 |
| H | -5.81211600 | -1.95971900 | -5.34794200 |
| Lu | -0.23810200 | -0.01542200 | 0.25598400 |
| Lu | 2.63862300 | 1.68117000 | -0.77333500 |
| Lu | 2.96849000 | -1.68850200 | 0.45366700 |

**4A**-III

| | | | |
|---|---|---|---|
| C | -1.81861400 | 2.50410000 | 3.26629600 |
| C | -2.51609500 | 1.38019600 | 3.84698000 |
| C | -1.84063600 | 0.13997300 | 4.13032600 |
| C | -0.44128200 | 0.10088100 | 3.80891300 |
| C | -3.89230400 | 1.40467400 | 3.36739500 |
| C | -2.61820800 | -1.09296600 | 4.03364200 |
| C | -4.03397800 | -1.08861500 | 3.57270000 |
| C | -4.66567500 | 0.18766600 | 3.18180800 |
| C | -1.90352600 | -0.06639900 | -4.08789900 |
| C | -1.16160900 | 1.14688200 | -3.87878400 |
| C | 0.17531900 | 1.12492200 | -3.35097800 |
| C | 0.77766200 | -0.10302800 | -3.00003700 |
| C | 0.06697300 | -1.31346000 | -3.29233700 |
| C | -1.24540000 | -1.34307600 | -3.90086200 |
| C | 0.40526300 | 2.32820200 | -2.57526300 |
| C | 1.66466300 | -0.09960800 | -1.87175800 |
| C | 1.93055500 | 1.10693200 | -1.12766400 |
| C | 1.32774700 | 2.35757500 | -1.45561900 |
| C | 1.15709500 | 2.73101000 | 0.97949500 |
| C | 1.77947200 | 1.46149200 | 1.27206800 |
| C | 0.25323600 | 1.23153400 | 3.23850300 |
| C | -0.43056100 | 2.42637100 | 2.92677800 |
| C | 0.03426600 | 3.16160600 | 1.78499300 |
| C | 1.31470700 | 0.75217000 | 2.39376600 |
| C | -5.47828400 | -1.35198300 | -1.29666800 |
| C | -5.06574500 | -0.66268800 | -2.47553000 |
| C | -4.00775900 | -1.18066600 | -3.32226700 |
| C | -3.36794100 | -2.46469800 | -3.11109800 |
| C | -3.79284300 | -3.12885800 | -1.89275900 |
| C | -4.83621200 | -2.59834000 | -1.02902400 |
| C | -3.30717000 | -0.01956700 | -3.85029400 |
| C | -1.93770600 | -2.56776300 | -3.47208600 |
| C | -4.57703700 | -3.01066800 | 0.32363600 |
| C | -5.93805700 | -0.57718100 | -0.18625000 |
| C | -5.67174200 | -0.99518600 | 1.16008000 |
| C | -4.96637300 | -2.19593100 | 1.44164300 |
| C | 1.29041200 | -0.70346500 | 2.45528600 |

| | | | |
|---|---|---|---|
| C | 0.20595600 | -1.08678100 | 3.31980200 |
| C | -2.73114400 | 3.22357600 | 2.42977400 |
| C | -3.99682100 | 2.54073700 | 2.46827600 |
| C | 0.21116500 | -2.45584600 | -2.45023900 |
| C | -1.00386200 | -3.24246100 | -2.53316200 |
| C | -3.94320800 | 1.17161100 | -3.36012800 |
| C | -5.02506400 | 0.77324800 | -2.50962500 |
| C | -0.54775600 | -2.26127800 | 3.07882800 |
| C | -1.95500600 | -2.22656000 | 3.41478600 |
| C | -1.45495900 | -3.86172000 | -1.29965700 |
| C | -0.85959700 | 3.93480200 | 0.96500200 |
| C | -2.28429600 | 3.91133100 | 1.25064700 |
| C | -3.18484400 | 2.35818200 | -3.08185900 |
| C | 1.09497400 | -2.40982600 | -1.30683000 |
| C | -4.15411800 | -2.21760300 | 2.64309700 |
| C | -2.89537800 | -2.92109500 | 2.58552900 |
| C | -2.47375800 | -3.70102900 | 1.44369300 |
| C | -3.36221900 | -3.77265500 | 0.33074500 |
| C | -2.86119000 | -3.83789800 | -1.02248700 |
| C | 1.68858700 | -1.48510100 | 1.36210400 |
| C | 1.89045400 | -1.25277100 | -1.09511400 |
| C | 0.96969300 | -2.68802300 | 1.14653500 |
| C | -0.13616100 | -3.07171900 | 1.98642900 |
| C | -1.08240900 | -3.79107000 | 1.18566300 |
| C | -0.56683200 | -3.85574900 | -0.16596800 |
| C | 0.70363100 | -3.18964900 | -0.17992400 |
| C | -5.47289800 | 0.18666500 | 1.98694300 |
| C | -5.60548100 | 1.33234400 | 1.10651200 |
| C | -5.88311000 | 0.86074800 | -0.21766800 |
| C | -5.39023300 | 1.55536000 | -1.36622200 |
| C | -4.83998700 | 2.51914400 | 1.31515900 |
| C | 1.00989800 | 3.30245100 | -0.36128100 |
| C | -0.28925000 | 4.03270200 | -0.37018600 |
| C | -1.23542700 | 3.89850900 | -1.47564500 |
| C | -1.79316900 | 2.33912900 | -3.38799200 |
| C | -0.83134900 | 3.07782700 | -2.59353200 |
| C | -2.64793800 | 3.91203600 | -1.19891700 |
| C | -3.17062300 | 3.96270700 | 0.14714000 |
| C | -4.43757400 | 3.27219800 | 0.17270400 |
| C | -4.69444800 | 2.78325100 | -1.15061000 |
| C | -3.60151900 | 3.18170700 | -1.99949500 |
| N | -1.86113900 | 0.04687600 | -0.00406500 |
| C | 2.54068700 | -0.89206100 | 0.25137900 |
| C | 2.61870300 | 0.76425100 | 0.20150700 |
| C | 4.89704300 | 0.21605600 | -0.71741100 |
| H | 5.96964400 | 0.42922000 | -0.69066500 |
| H | 4.55258700 | 0.36462900 | -1.74270500 |
| Si | 4.47980500 | -1.49242200 | 0.02237400 |
| C | 4.14441200 | 1.16822200 | 0.23993700 |
| H | 4.47179600 | 0.94972300 | 1.26619500 |
| C | 4.40432400 | -3.12194000 | -0.99665600 |
| C | 4.21853800 | -4.34016000 | -0.28255800 |
| C | 4.53367500 | -3.17700800 | -2.41586800 |
| C | 4.16531100 | -5.55597700 | -0.97846700 |
| C | 4.47430000 | -4.41356500 | -3.06875500 |
| C | 4.29170300 | -5.59668300 | -2.36009000 |
| H | 4.01979400 | -6.48191900 | -0.43324000 |
| H | 4.57009300 | -4.45857900 | -4.14753400 |
| C | 5.87102200 | -1.66769900 | 1.38178800 |
| C | 5.73978600 | -1.40270000 | 2.76724600 |
| C | 7.18001300 | -1.99906700 | 0.89148400 |
| C | 6.85615400 | -1.46433700 | 3.61760200 |
| C | 8.26112700 | -2.06171000 | 1.77706300 |
| C | 8.10978000 | -1.79752900 | 3.13558200 |
| H | 6.72465200 | -1.23600500 | 4.67284600 |
| H | 9.24653700 | -2.31190800 | 1.40142300 |
| C | 4.43058500 | 2.64470500 | 0.01210200 |
| C | 4.57439300 | 3.50575000 | 1.11012200 |
| C | 4.57421400 | 3.20492000 | -1.26204200 |
| C | 4.82895200 | 4.86362800 | 0.93950600 |

| | | | |
|---|---|---|---|
| H | 4.48666700 | 3.10450500 | 2.11752500 |
| C | 4.82710700 | 4.56832000 | -1.43188100 |
| H | 4.49183600 | 2.57778300 | -2.14485200 |
| C | 4.95644700 | 5.43460300 | -0.33818700 |
| H | 4.93307400 | 5.48636200 | 1.82384800 |
| H | 4.92579700 | 4.94811200 | -2.44292300 |
| C | 5.23607100 | 6.94061300 | -0.48720000 |
| C | 6.58577700 | 7.28170600 | 0.18864900 |
| H | 7.40949400 | 6.73311000 | -0.28248900 |
| H | 6.79724200 | 8.35457700 | 0.10123200 |
| H | 6.58235500 | 7.02821100 | 1.25406500 |
| C | 4.10359800 | 7.74443800 | 0.19556400 |
| H | 3.13428800 | 7.52577300 | -0.26684000 |
| H | 4.02409300 | 7.51201200 | 1.26270300 |
| H | 4.29080900 | 8.82126400 | 0.10230900 |
| C | 5.31176400 | 7.37922800 | -1.96209400 |
| H | 6.12374300 | 6.87440400 | -2.49807600 |
| H | 4.37442500 | 7.18102800 | -2.49446600 |
| H | 5.50137700 | 8.45721400 | -2.01735800 |
| C | 4.73958900 | -1.91968400 | -3.25481200 |
| H | 3.89019600 | -1.24694200 | -3.09053800 |
| H | 5.61820000 | -1.37959400 | -2.88140400 |
| C | 4.10833000 | -4.37498100 | 1.23892300 |
| H | 5.11573100 | -4.31328900 | 1.67097800 |
| H | 3.58942900 | -3.47595500 | 1.58497000 |
| C | 4.43603500 | -1.03052700 | 3.44296500 |
| H | 4.57163600 | -0.08053700 | 3.97922600 |
| H | 3.66372500 | -0.84720200 | 2.70161100 |
| C | 7.45232100 | -2.29874900 | -0.58117500 |
| H | 6.99056500 | -3.25710600 | -0.84392200 |
| H | 6.94122400 | -1.55309400 | -1.20045300 |
| C | 8.92062000 | -2.33223600 | -1.02529700 |
| H | 9.44396300 | -1.40060700 | -0.78180100 |
| H | 9.47259300 | -3.16067300 | -0.56845200 |
| H | 8.97065700 | -2.47108500 | -2.11082000 |
| C | 3.38276300 | -5.58436200 | 1.84382500 |
| H | 3.93923300 | -6.51711300 | 1.70327000 |
| H | 3.26368800 | -5.44183500 | 2.92363300 |
| H | 2.38564100 | -5.71303000 | 1.40898000 |
| C | 4.91005900 | -2.10870400 | -4.76688300 |
| H | 5.77825300 | -2.73213300 | -5.00852600 |
| H | 4.02666400 | -2.56608000 | -5.22610800 |
| H | 5.06052000 | -1.13411700 | -5.24403600 |
| H | 8.96724100 | -1.84184600 | 3.80225100 |
| H | 4.24686500 | -6.54681600 | -2.88650000 |
| C | 3.92385800 | -2.08523900 | 4.43982100 |
| H | 4.64703500 | -2.26056400 | 5.24362900 |
| H | 2.98378200 | -1.75663800 | 4.89593100 |
| H | 3.74100300 | -3.04443700 | 3.94411800 |
| Lu | -0.69649700 | 1.71480700 | -0.32675700 |
| Lu | -2.03721800 | -1.29886100 | -1.53341700 |
| Lu | -2.77943800 | -0.27796100 | 1.79065700 |

**4B**-I

| | | | |
|---|---|---|---|
| C | 1.72567500 | 2.21596100 | -3.33134000 |
| C | 2.35681600 | 1.03501700 | -3.83650300 |
| C | 1.63010100 | -0.19066500 | -3.95408400 |
| C | 0.24315200 | -0.18045600 | -3.62603200 |
| C | 3.75271300 | 1.04976400 | -3.43521000 |
| C | 2.36149900 | -1.39888300 | -3.73808000 |
| C | 3.75494900 | -1.37067600 | -3.32842700 |
| C | 4.52488800 | -0.15380500 | -3.21403900 |
| C | 2.14864300 | 0.14532200 | 4.12107500 |
| C | 1.42832600 | 1.36251700 | 3.89162700 |
| C | 0.06368400 | 1.33965000 | 3.44941100 |
| C | -0.59228100 | 0.10105000 | 3.21473100 |
| C | 0.07114400 | -1.10861700 | 3.55344600 |
| C | 1.43607900 | -1.08997700 | 3.99565400 |
| C | -0.18967200 | 2.50665300 | 2.62848900 |
| C | -1.51192300 | 0.05438100 | 2.11941800 |

| | | | |
|---|---|---|---|
| C | -1.81985000 | 1.21678400 | 1.34141100 |
| C | -1.16299400 | 2.47517700 | 1.55498800 |
| C | -1.09318900 | 2.65197100 | -0.90072700 |
| C | -1.78010300 | 1.39643600 | -1.08221700 |
| C | -0.38295100 | 1.00221100 | -3.10522500 |
| C | 0.35589900 | 2.19286300 | -2.91385500 |
| C | -0.01656700 | 3.00277400 | -1.79364200 |
| C | -1.40938200 | 0.61258000 | -2.17436700 |
| C | 5.52767400 | -1.42553500 | 1.27329500 |
| C | 5.21117100 | -0.64003100 | 2.41453500 |
| C | 4.18419300 | -1.04960300 | 3.33333800 |
| C | 3.44785900 | -2.25480600 | 3.12231300 |
| C | 3.82675700 | -3.08515400 | 2.02873400 |
| C | 4.86226100 | -2.67910600 | 1.11971600 |
| C | 3.54080900 | 0.13705900 | 3.82866500 |
| C | 2.06964700 | -2.27828600 | 3.50908900 |
| C | 4.53868200 | -3.17790600 | -0.18856800 |
| C | 5.93526400 | -0.73124700 | 0.06956300 |
| C | 5.70201700 | -1.24685600 | -1.26126500 |
| C | 4.88394600 | -2.44744500 | -1.37200900 |
| C | -1.40747300 | -0.85484500 | -2.11339200 |
| C | -0.37770000 | -1.31777700 | -3.01004100 |
| C | 2.69592800 | 2.97608300 | -2.59912900 |
| C | 3.94587500 | 2.25290900 | -2.64884700 |
| C | -0.16974100 | -2.33178400 | 2.82066400 |
| C | 1.08440900 | -3.06660800 | 2.79002700 |
| C | 4.17746300 | 1.28096100 | 3.23537900 |
| C | 5.20826400 | 0.80171600 | 2.35429900 |
| C | 0.36536100 | -2.48661800 | -2.72676300 |
| C | 1.73530900 | -2.53807000 | -3.14037300 |
| C | 1.44927300 | -3.89976800 | 1.66348600 |
| C | 0.92679500 | 3.82059000 | -1.08444700 |
| C | 2.32965000 | 3.74499600 | -1.45010300 |
| C | 3.43570600 | 2.46143000 | 2.92758200 |
| C | -1.13566700 | -2.38709800 | 1.74537300 |
| C | 3.95258200 | -2.50789700 | -2.45138700 |
| C | 2.70940000 | -3.23317300 | -2.34800600 |
| C | 2.34555200 | -3.90307100 | -1.13708200 |
| C | 3.29313300 | -3.90121200 | -0.08348600 |
| C | 2.84161500 | -3.84654800 | 1.29103100 |
| C | -1.77257800 | -1.54448400 | -0.95744500 |
| C | -1.81595500 | -1.16358500 | 1.43321300 |
| C | -1.07735300 | -2.77874500 | -0.67968900 |
| C | -0.00326400 | -3.20254900 | -1.54157200 |
| C | 0.94148600 | -3.96020900 | -0.76783800 |
| C | 0.44741400 | -4.05868400 | 0.60228700 |
| C | -0.84844400 | -3.33211600 | 0.65206600 |
| C | 5.58899900 | -0.10730800 | -2.19535500 |
| C | 5.72119700 | 1.11381100 | -1.36483500 |
| C | 5.94245500 | 0.70747100 | 0.00859700 |
| C | 5.52475400 | 1.49299200 | 1.15336400 |
| C | 4.88529200 | 2.29143100 | -1.57138100 |
| C | -0.87095200 | 3.31788300 | 0.38076900 |
| C | 0.43035700 | 4.02978500 | 0.27047400 |
| C | 1.43138800 | 3.96448800 | 1.33788700 |
| C | 2.05654400 | 2.50985500 | 3.31035100 |
| C | 1.06658000 | 3.23494900 | 2.53332600 |
| C | 2.82579200 | 3.89331400 | 0.97187500 |
| C | 3.27691600 | 3.83561300 | -0.40136000 |
| C | 4.52522800 | 3.10923000 | -0.44824400 |
| C | 4.85072000 | 2.72483200 | 0.89801400 |
| C | 3.81293300 | 3.20160400 | 1.77021900 |
| N | 1.89636600 | -0.01744500 | 0.11632100 |
| C | -2.58020400 | -0.86253600 | 0.14600400 |
| C | -2.58035200 | 0.80481800 | 0.07647400 |
| Si | -4.51554900 | -1.37487100 | -0.21557900 |
| C | -4.09105200 | 1.26284500 | 0.05493100 |
| H | -4.47608400 | 1.02651400 | 1.05668700 |
| C | -4.81937600 | 0.35844200 | -0.96046200 |
| H | -4.39639500 | 0.50014700 | -1.95731900 |

| | | | |
|---|---|---|---|
| H | -5.88015500 | 0.62105400 | -1.00476400 |
| C | -4.32252300 | 2.75346100 | -0.13850000 |
| C | -4.50429700 | 3.58293700 | 0.97748100 |
| C | -4.38403500 | 3.35508300 | -1.40042400 |
| C | -4.71770900 | 4.95168600 | 0.83653600 |
| H | -4.48019500 | 3.14959900 | 1.97498900 |
| C | -4.59879800 | 4.72804400 | -1.53999600 |
| H | -4.26607100 | 2.75277600 | -2.29667300 |
| C | -4.76735500 | 5.56344100 | -0.42731500 |
| H | -4.85261600 | 5.54980400 | 1.73359700 |
| H | -4.63612300 | 5.14066300 | -2.54222800 |
| C | -5.00633300 | 7.07937700 | -0.54278400 |
| C | -6.37724700 | 7.43270600 | 0.08207900 |
| H | -6.56086900 | 8.51221700 | 0.01571100 |
| H | -7.19071300 | 6.91669700 | -0.44085500 |
| H | -6.42766700 | 7.15053000 | 1.13902200 |
| C | -5.00596400 | 7.56305500 | -2.00545000 |
| H | -4.04944800 | 7.36175600 | -2.50127500 |
| H | -5.80238000 | 7.09138500 | -2.59257900 |
| H | -5.17206200 | 8.64585600 | -2.03585600 |
| C | -3.88789800 | 7.83481900 | 0.21432800 |
| H | -3.86146600 | 7.56740700 | 1.27588000 |
| H | -2.90398900 | 7.60763100 | -0.21170000 |
| H | -4.04662100 | 8.91811900 | 0.14711800 |
| C | -4.48175400 | -2.97632600 | -1.28422400 |
| C | -4.37915000 | -4.22456000 | -0.60347300 |
| C | -4.57328900 | -2.98383600 | -2.70754700 |
| C | -4.37331800 | -5.42033700 | -1.33288000 |
| C | -4.56320500 | -4.20391500 | -3.39509300 |
| C | -4.46585900 | -5.41438400 | -2.71848900 |
| H | -4.29154400 | -6.36796400 | -0.81254500 |
| H | -4.62907900 | -4.21251100 | -4.47682100 |
| C | -5.94433200 | -1.52399100 | 1.09771200 |
| C | -5.81698300 | -1.38699100 | 2.50834100 |
| C | -7.25337000 | -1.73522200 | 0.56077200 |
| C | -6.94939800 | -1.45402800 | 3.32950000 |
| C | -8.35881200 | -1.79449400 | 1.42100300 |
| C | -8.21506600 | -1.65590700 | 2.79452600 |
| H | -6.84217600 | -1.34727800 | 4.40285400 |
| H | -9.34950600 | -1.95284100 | 1.01042300 |
| C | -4.29751100 | -4.30425000 | 0.91805700 |
| H | -3.67202700 | -3.48394400 | 1.28632800 |
| H | -5.29412600 | -4.12308900 | 1.33973700 |
| C | -4.67915100 | -1.69610500 | -3.51895800 |
| H | -5.53287700 | -1.11124100 | -3.15576500 |
| H | -3.79303500 | -1.08253000 | -3.31852100 |
| C | -7.50817600 | -1.93987000 | -0.93129000 |
| H | -6.86846100 | -1.26460500 | -1.50696200 |
| H | -7.18456700 | -2.94972200 | -1.21212800 |
| C | -4.46273500 | -1.16636900 | 3.16215400 |
| H | -3.77709800 | -1.95021200 | 2.82422800 |
| H | -4.03726100 | -0.22808100 | 2.78731800 |
| H | -4.45868100 | -6.35014300 | -3.27172900 |
| H | -9.08463100 | -1.70483800 | 3.44501000 |
| C | -3.74041500 | -5.60452700 | 1.51181300 |
| H | -3.63926900 | -5.49958600 | 2.59755200 |
| H | -4.40097700 | -6.45934800 | 1.33118900 |
| H | -2.75258900 | -5.84301900 | 1.10291100 |
| C | -4.81845300 | -1.83833800 | -5.03919300 |
| H | -5.71447100 | -2.40354600 | -5.31956900 |
| H | -4.89836100 | -0.84512000 | -5.49435600 |
| H | -3.95018800 | -2.33476300 | -5.48616100 |
| C | -8.94775600 | -1.73470200 | -1.42298200 |
| H | -8.97451600 | -1.80219500 | -2.51607700 |
| H | -9.63240700 | -2.49750500 | -1.03758700 |
| H | -9.33956400 | -0.75202200 | -1.13662300 |
| C | -4.41268800 | -1.12441100 | 4.69328800 |
| H | -5.00194500 | -0.29628600 | 5.10254800 |
| H | -4.77985300 | -2.05525400 | 5.13985300 |
| H | -3.37698200 | -0.98505800 | 5.02122400 |

| | | | |
|---|---|---|---|
| Lu | 0.90672800 | -1.75711600 | 0.58261100 |
| Lu | 0.85302000 | 1.72074300 | 0.46951000 |
| Lu | 3.61642700 | -0.00010900 | -0.98393000 |

**4B**-II

| | | | |
|---|---|---|---|
| C | -0.24619500 | -1.20271900 | -3.50346700 |
| C | -1.62576600 | -1.19882600 | -3.90022700 |
| C | -2.33490000 | 0.03272100 | -4.03520500 |
| C | -1.60747600 | 1.24660000 | -3.88115000 |
| C | -2.25789400 | -2.38066700 | -3.35191600 |
| C | -3.71614100 | 0.02272900 | -3.67175100 |
| C | -4.33287400 | -1.15884000 | -3.10809200 |
| C | -3.64992000 | -2.42152100 | -2.96981400 |
| C | -1.49407800 | -0.08208300 | 4.04384300 |
| C | -0.11366100 | -0.08083300 | 3.66670300 |
| C | 0.51025300 | 1.08661400 | 3.11285700 |
| C | -0.24338800 | 2.27928300 | 2.91308900 |
| C | -1.60507400 | 2.31892100 | 3.36453700 |
| C | -2.21978100 | 1.14864700 | 3.91038500 |
| C | 1.54741300 | 0.67269600 | 2.18467000 |
| C | 0.07662600 | 3.05663600 | 1.77562100 |
| C | 1.09498200 | 2.63252100 | 0.84112200 |
| C | 1.88799900 | 1.46234600 | 1.04468700 |
| C | 1.81481900 | 1.17215800 | -1.46222000 |
| C | 0.98979600 | 2.34422100 | -1.61345200 |
| C | 0.71961300 | 3.14611400 | -0.46077100 |
| C | -0.22503600 | 1.22828500 | -3.47534200 |
| C | 0.45750600 | 0.00997400 | -3.23717200 |
| C | 1.48999100 | -0.00413900 | -2.21029300 |
| C | 0.02822500 | 2.38597700 | -2.66285400 |
| C | -4.80849600 | -2.38541200 | 1.64854800 |
| C | -3.86528400 | -2.43749100 | 2.71050000 |
| C | -3.61874300 | -1.29054000 | 3.54265400 |
| C | -4.30892500 | -0.06469800 | 3.31605300 |
| C | -5.31750700 | -0.04036800 | 2.31240200 |
| C | -5.57514800 | -1.19127900 | 1.49336800 |
| C | -2.22320000 | -1.29680500 | 3.89881700 |
| C | -3.61032500 | 1.16417600 | 3.55790800 |
| C | -5.97225300 | -0.73859700 | 0.18780000 |
| C | -4.52312600 | -3.15571400 | 0.45709100 |
| C | -4.96719700 | -2.75955600 | -0.85894300 |
| C | -5.62017100 | -1.46697400 | -0.99282000 |
| C | -1.20788400 | 3.11017500 | -2.53396200 |
| C | -2.21823200 | 2.39915100 | -3.27682500 |
| C | -0.00182400 | -2.37280400 | -2.71791800 |
| C | -1.24221200 | -3.10371200 | -2.60995800 |
| C | -2.61639400 | 3.08506400 | 2.67010000 |
| C | -3.88255000 | 2.36938400 | 2.80004800 |
| C | -1.61506600 | -2.45574200 | 3.31279700 |
| C | -2.62616300 | -3.16291800 | 2.57018800 |
| C | -3.56390800 | 2.35837000 | -2.82646200 |
| C | -4.32679400 | 1.17720600 | -3.08139400 |
| C | -4.89083600 | 2.40903600 | 1.75986700 |
| C | 1.80523500 | -1.20347100 | -1.48541700 |
| C | 0.96387000 | -2.35623100 | -1.66587300 |
| C | -0.26213000 | -2.41853800 | 2.85914300 |
| C | -2.30080400 | 3.85590200 | 1.49274500 |
| C | -5.32647500 | -0.70392500 | -2.15822800 |
| C | -5.32854800 | 0.73581500 | -2.15120000 |
| C | -5.60042200 | 1.45701900 | -0.94780200 |
| C | -5.95160800 | 0.70181000 | 0.20128700 |
| C | -5.55249300 | 1.15039800 | 1.51967200 |
| C | -1.52083400 | 3.81485300 | -1.33892600 |
| C | -0.92416100 | 3.81122500 | 1.06900900 |
| C | -0.52368800 | 3.84064100 | -0.32100400 |
| C | -2.90179300 | 3.85916900 | -0.91337500 |
| C | -3.88686200 | 3.11005500 | -1.63939200 |
| C | -4.92487400 | 2.71849300 | -0.72247200 |
| C | -4.62966900 | 3.26900800 | 0.59856900 |
| C | -3.33494300 | 3.98571300 | 0.47258700 |

| | | | |
|---|---|---|---|
| C | -4.03661000 | -3.30281800 | -1.85716900 |
| C | -2.97963900 | -4.01805100 | -1.09568900 |
| C | -3.30646500 | -3.91306900 | 0.31970200 |
| C | -2.30799500 | -3.85896700 | 1.37113600 |
| C | -1.57169100 | -3.88821300 | -1.45613100 |
| C | 1.86685900 | -1.55698000 | 1.00138000 |
| C | 0.50086500 | -1.23411900 | 3.08608000 |
| C | 1.53960800 | -0.79987000 | 2.16484800 |
| C | 1.07042600 | -2.72081900 | 0.77503600 |
| C | 0.67490900 | -3.17967300 | -0.53943400 |
| C | -0.57385800 | -3.88527100 | -0.41785700 |
| C | -0.93710700 | -3.89725400 | 0.97797900 |
| C | 0.06455700 | -3.17887200 | 1.70488100 |
| N | -1.81398600 | -0.01422300 | -0.00043700 |
| C | 2.62851600 | 0.83573300 | -0.18406200 |
| C | 2.62205500 | -0.90408100 | -0.20122300 |
| Si | 4.58481000 | -1.37722800 | 0.12234400 |
| C | 4.55325600 | -2.92672500 | 1.25888400 |
| C | 4.71983900 | -2.88503100 | 2.67423000 |
| C | 4.37223400 | -4.19228500 | 0.62946900 |
| C | 4.72037000 | -4.07997400 | 3.40441500 |
| C | 4.38059700 | -5.36156400 | 1.40031100 |
| C | 4.55742100 | -5.30997000 | 2.77697200 |
| H | 4.84504900 | -4.05333600 | 4.48072400 |
| H | 4.24168300 | -6.32408000 | 0.92072900 |
| H | 4.56083500 | -6.22572800 | 3.36267600 |
| C | 4.88462200 | -1.57001000 | 3.42971600 |
| H | 5.71587700 | -1.00503300 | 2.99256200 |
| H | 3.99019100 | -0.95759000 | 3.26346700 |
| C | 4.18144900 | -4.31772700 | -0.87930100 |
| H | 3.58537300 | -3.47197900 | -1.24025900 |
| H | 5.15612600 | -4.21737400 | -1.37365900 |
| C | 3.50445500 | -5.60025100 | -1.38093300 |
| H | 2.53733400 | -5.76266300 | -0.89280400 |
| H | 3.32887400 | -5.52589300 | -2.45963500 |
| H | 4.12406000 | -6.48811300 | -1.21578400 |
| C | 5.98302000 | -1.62382700 | -1.20967000 |
| C | 7.30861200 | -1.79119900 | -0.68157300 |
| C | 5.83127700 | -1.63672700 | -2.61917400 |
| C | 8.39216500 | -1.94053800 | -1.55344200 |
| C | 6.94733700 | -1.80645800 | -3.45497600 |
| C | 8.22164900 | -1.95204600 | -2.93466600 |
| H | 9.39073900 | -2.06096300 | -1.14964800 |
| H | 6.79748700 | -1.82978900 | -4.53171300 |
| H | 9.07745300 | -2.08148700 | -3.59222500 |
| C | 7.59452900 | -1.86240600 | 0.81655600 |
| H | 6.99799900 | -1.10973700 | 1.33960200 |
| H | 7.23849400 | -2.82718400 | 1.19807800 |
| C | 9.05277000 | -1.67477700 | 1.25689900 |
| H | 9.69680000 | -2.49534100 | 0.92364700 |
| H | 9.47559700 | -0.73664800 | 0.87961400 |
| H | 9.10374900 | -1.65005700 | 2.35089600 |
| C | 4.50427100 | -1.49970000 | -3.33913100 |
| H | 4.36028100 | -2.38052100 | -3.98096000 |
| H | 3.68019600 | -1.51357200 | -2.62920600 |
| C | 5.11946000 | -1.65898300 | 4.94208800 |
| H | 5.23955000 | -0.65131900 | 5.35454600 |
| H | 4.27645300 | -2.12731200 | 5.46162600 |
| H | 6.02540800 | -2.22603900 | 5.18424200 |
| C | 4.38342700 | 2.74809600 | -0.14978400 |
| C | 4.49814700 | 3.51436100 | -1.31402100 |
| C | 4.51076700 | 3.41668900 | 1.07659700 |
| C | 4.71900400 | 4.89258800 | -1.26253000 |
| H | 4.41519900 | 3.02913700 | -2.28407900 |
| C | 4.73121700 | 4.79057600 | 1.12547200 |
| H | 4.44126000 | 2.86298900 | 2.00853200 |
| C | 4.83916100 | 5.56759700 | -0.04128900 |
| H | 4.79990100 | 5.43430000 | -2.19827500 |
| H | 4.82314800 | 5.26359700 | 2.09938200 |
| C | 5.08298500 | 7.08377400 | 0.05992500 |

| | | | |
|---|---|---|---|
| C | 6.41309200 | 7.34335700 | 0.80711300 |
| H | 6.59726700 | 8.42125500 | 0.89358600 |
| H | 6.40043700 | 6.92544500 | 1.81929100 |
| H | 7.25797200 | 6.89597100 | 0.27099100 |
| C | 3.91967200 | 7.73954600 | 0.84199700 |
| H | 2.96316200 | 7.57302600 | 0.33366000 |
| H | 3.83283200 | 7.33776000 | 1.85693600 |
| H | 4.07809500 | 8.82191100 | 0.92427600 |
| C | 5.16963300 | 7.75608300 | -1.32355300 |
| H | 5.99645200 | 7.35566800 | -1.92136800 |
| H | 4.24265100 | 7.63337000 | -1.89520800 |
| H | 5.34184600 | 8.83138800 | -1.20108800 |
| C | 4.39292700 | -0.23720300 | -4.21261300 |
| H | 3.41543600 | -0.19397600 | -4.70541100 |
| H | 4.51008300 | 0.67332600 | -3.61429600 |
| H | 5.16254000 | -0.21802000 | -4.99167700 |
| C | 4.14073800 | 1.24987800 | -0.25021700 |
| H | 4.46599700 | 0.95401300 | -1.25733900 |
| C | 4.90486800 | 0.38958600 | 0.77374500 |
| H | 4.52468000 | 0.57534700 | 1.78090200 |
| H | 5.96567900 | 0.65458300 | 0.75990100 |
| Lu | 0.23719900 | -0.03007500 | 0.05520700 |
| Lu | -2.72759300 | -1.67630000 | -0.82489300 |
| Lu | -2.94286900 | 1.63494900 | 0.54328100 |

**4B**-III

| | | | |
|---|---|---|---|
| C | -1.55763700 | 2.25381700 | 3.40099500 |
| C | -2.22271300 | 1.09292900 | 3.94559700 |
| C | -1.54241200 | -0.16937100 | 4.08630000 |
| C | -0.16980500 | -0.19390700 | 3.65967400 |
| C | -3.63022200 | 1.16276100 | 3.57173300 |
| C | -2.33950200 | -1.38632500 | 3.95336900 |
| C | -3.78491000 | -1.33708200 | 3.59760000 |
| C | -4.42881100 | -0.03159600 | 3.35172900 |
| C | -2.21342400 | 0.25277600 | -4.09689600 |
| C | -1.44272200 | 1.44098800 | -3.85498400 |
| C | -0.07058900 | 1.37109200 | -3.43253100 |
| C | 0.54217400 | 0.11616800 | -3.21947900 |
| C | -0.20452700 | -1.06377600 | -3.54761100 |
| C | -1.55930800 | -1.03757900 | -4.05307500 |
| C | 0.23104000 | 2.51269600 | -2.59054500 |
| C | 1.51259900 | 0.02735600 | -2.16692100 |
| C | 1.84790600 | 1.17511900 | -1.35772400 |
| C | 1.23350300 | 2.44958700 | -1.54279700 |
| C | 1.23616200 | 2.62897900 | 0.91902400 |
| C | 1.86080300 | 1.33736000 | 1.06807100 |
| C | 0.49140200 | 0.97393200 | 3.12460900 |
| C | -0.20024900 | 2.19277200 | 2.95342800 |
| C | 0.18513700 | 3.00732100 | 1.83703100 |
| C | 1.48185200 | 0.55401200 | 2.17032700 |
| C | -5.58644000 | -1.22049100 | -1.15696700 |
| C | -5.25352800 | -0.44581300 | -2.30708100 |
| C | -4.26590500 | -0.90351700 | -3.26519300 |
| C | -3.62816600 | -2.20340900 | -3.19921100 |
| C | -3.97287700 | -2.95996600 | -2.00941700 |
| C | -4.94245100 | -2.48910400 | -1.03300900 |
| C | -3.59522200 | 0.29065600 | -3.75687300 |
| C | -2.23038900 | -2.28827700 | -3.67273300 |
| C | -4.58855200 | -3.00432600 | 0.26197500 |
| C | -5.95386500 | -0.53004000 | 0.03986300 |
| C | -5.59395300 | -1.05084100 | 1.32697400 |
| C | -4.88467000 | -2.27401500 | 1.46401000 |
| C | 1.45480900 | -0.90344600 | 2.12731500 |
| C | 0.42777700 | -1.34500300 | 3.03699800 |
| C | -2.52054200 | 3.04069500 | 2.69076800 |
| C | -3.78716400 | 2.36427600 | 2.77094800 |
| C | -0.00998500 | -2.27028200 | -2.81267300 |
| C | -1.23639800 | -3.04411800 | -2.86550200 |
| C | -4.17991600 | 1.44437900 | -3.13333000 |
| C | -5.19979100 | 0.98840300 | -2.23627100 |

| | | | |
|---|---|---|---|
| C | -0.35752600 | -2.49108800 | 2.76225800 |
| C | -1.73637200 | -2.47291400 | 3.20237100 |
| C | -1.60465500 | -3.75325000 | -1.65122600 |
| C | -0.75475700 | 3.84938500 | 1.14757500 |
| C | -2.15415300 | 3.81398500 | 1.53692200 |
| C | -3.38838200 | 2.60122200 | -2.82339500 |
| C | 0.95776800 | -2.31391200 | -1.73720300 |
| C | -3.98545300 | -2.39018700 | 2.59599200 |
| C | -2.74348100 | -3.09576800 | 2.39396800 |
| C | -2.41702300 | -3.78950500 | 1.16808500 |
| C | -3.38513500 | -3.77182500 | 0.12159000 |
| C | -2.98728400 | -3.74040600 | -1.26831300 |
| C | 1.74411600 | -1.59333700 | 0.94320500 |
| C | 1.78454800 | -1.18458400 | -1.49830400 |
| C | 1.00102900 | -2.77233000 | 0.69204800 |
| C | -0.04015800 | -3.21555700 | 1.58093100 |
| C | -1.04987100 | -3.86894400 | 0.80185300 |
| C | -0.63771300 | -3.83133300 | -0.58615100 |
| C | 0.63479700 | -3.17124100 | -0.64246100 |
| C | -5.32159600 | 0.06327800 | 2.22369100 |
| C | -5.50514700 | 1.27335700 | 1.44452100 |
| C | -5.88539900 | 0.90563000 | 0.11296800 |
| C | -5.47008300 | 1.68247200 | -1.01317000 |
| C | -4.71384600 | 2.43644900 | 1.68547900 |
| C | 1.00398700 | 3.30630600 | -0.35724600 |
| C | -0.28242800 | 4.04631000 | -0.21495900 |
| C | -1.30768400 | 4.00448700 | -1.25522600 |
| C | -2.02249000 | 2.59673700 | -3.23183000 |
| C | -0.99565300 | 3.26912400 | -2.45923600 |
| C | -2.69558100 | 4.00294000 | -0.87330500 |
| C | -3.11699200 | 3.95266600 | 0.50820900 |
| C | -4.38661000 | 3.27074200 | 0.57556900 |
| C | -4.74659500 | 2.88669800 | -0.75809000 |
| C | -3.71458400 | 3.34210100 | -1.65359500 |
| N | -1.88105800 | 0.05920000 | -0.03899600 |
| C | 2.53093300 | -0.92906700 | -0.18958800 |
| C | 2.61717300 | 0.72567500 | -0.11110300 |
| Si | 4.46120600 | -1.53207000 | 0.16348300 |
| C | 4.14487200 | 1.11630300 | -0.12150200 |
| H | 4.50121700 | 0.85167300 | -1.12744500 |
| C | 4.84819100 | 0.19088800 | 0.89139400 |
| H | 4.44400000 | 0.35622400 | 1.89260200 |
| H | 5.92005700 | 0.40649300 | 0.92140100 |
| C | 4.44336800 | 2.59731000 | 0.04790100 |
| C | 4.64354700 | 3.40254400 | -1.08266100 |
| C | 4.54419700 | 3.21480100 | 1.29956100 |
| C | 4.91008600 | 4.76407600 | -0.96553800 |
| H | 4.59001700 | 2.95529900 | -2.07286900 |
| C | 4.81113400 | 4.58096000 | 1.41535300 |
| H | 4.41490500 | 2.63052300 | 2.20618600 |
| H | 5.05633100 | 5.34346300 | -1.87307600 |
| H | 4.87559500 | 5.00738500 | 2.41048300 |
| C | 4.38196700 | -3.12147300 | 1.24702500 |
| C | 4.19949500 | -4.36422700 | 0.57462700 |
| C | 4.52499700 | -3.13020300 | 2.66561200 |
| C | 4.17823000 | -5.55888000 | 1.30487300 |
| C | 4.49591400 | -4.34897600 | 3.35523100 |
| H | 4.03705800 | -6.50269000 | 0.79015400 |
| H | 4.60190700 | -4.35924900 | 4.43381300 |
| C | 5.87976600 | -1.76128100 | -1.15667200 |
| C | 5.75363900 | -1.64500200 | -2.56949900 |
| C | 7.18168200 | -2.02242100 | -0.62247900 |
| C | 6.87693000 | -1.78281000 | -3.39450700 |
| C | 8.27818700 | -2.15065800 | -1.48677100 |
| H | 6.76842000 | -1.69207800 | -4.46920100 |
| H | 9.26239400 | -2.34661700 | -1.07704400 |
| C | 4.03600000 | -4.43448900 | -0.94026200 |
| H | 3.41881100 | -3.59130900 | -1.27031300 |
| H | 5.01431500 | -4.28580900 | -1.41450500 |
| C | 4.70209400 | -1.84625400 | 3.47060900 |

| | | | |
|---|---|---|---|
| H | 5.56309800 | -1.29126200 | 3.08006700 |
| H | 3.83179700 | -1.20305800 | 3.29674100 |
| C | 7.44053000 | -2.20535600 | 0.87150100 |
| H | 6.84424300 | -1.48244100 | 1.43502500 |
| H | 7.06678900 | -3.18916300 | 1.17988200 |
| C | 4.41003900 | -1.36869100 | -3.22193400 |
| H | 3.68357800 | -2.10108500 | -2.85615900 |
| H | 4.03811000 | -0.39869000 | -2.87179100 |
| C | 8.13443600 | -2.03421600 | -2.86211000 |
| H | 8.99720200 | -2.13805400 | -3.51525400 |
| C | 4.99617000 | 5.39275500 | 0.28787900 |
| C | 4.32848700 | -5.55560900 | 2.68553200 |
| H | 4.30917100 | -6.49007100 | 3.24075800 |
| C | 5.28860100 | 6.90107800 | 0.37574800 |
| C | 5.32062800 | 7.40814000 | 1.83019700 |
| H | 4.36313700 | 7.24815300 | 2.33913700 |
| H | 6.10645200 | 6.91851200 | 2.41689200 |
| H | 5.52422100 | 8.48489700 | 1.84119800 |
| C | 4.18826600 | 7.68212000 | -0.38198700 |
| H | 4.14203400 | 7.39964700 | -1.43895100 |
| H | 3.20179700 | 7.49400400 | 0.05695800 |
| H | 4.38352200 | 8.76046000 | -0.33313200 |
| C | 6.66369600 | 7.19723900 | -0.26945100 |
| H | 6.69358300 | 6.89344700 | -1.32122300 |
| H | 6.88320300 | 8.27113900 | -0.22594700 |
| H | 7.46548700 | 6.66484100 | 0.25508100 |
| C | 4.88450300 | -1.99165300 | 4.98611000 |
| H | 5.76904900 | -2.58675300 | 5.23998300 |
| H | 5.01260200 | -1.00114500 | 5.43630700 |
| H | 4.01436500 | -2.45774000 | 5.46137600 |
| C | 3.40154900 | -5.71140000 | -1.50639700 |
| H | 3.24635300 | -5.59891200 | -2.58502000 |
| H | 4.03936500 | -6.59016700 | -1.36263900 |
| H | 2.42897700 | -5.91582600 | -1.04559500 |
| C | 8.89361100 | -2.06531400 | 1.34655500 |
| H | 8.92617800 | -2.10825500 | 2.44077800 |
| H | 9.53365800 | -2.87231100 | 0.97467700 |
| H | 9.33434100 | -1.11177700 | 1.03399200 |
| C | 4.35165800 | -1.36874800 | -4.75321700 |
| H | 4.98043400 | -0.58544300 | -5.19102100 |
| H | 4.66579800 | -2.33033500 | -5.17462500 |
| H | 3.32237200 | -1.18354800 | -5.07946600 |
| Lu | -0.72307200 | 1.73740100 | -0.33232500 |
| Lu | -2.16148000 | -1.19917400 | -1.62522400 |
| Lu | -2.64842800 | -0.39937800 | 1.79782400 |
